# A Systematic Review of the Effectiveness of Assessing Skin Changes for Chronic Venous Insufficiency in People With Dark Skin Tones and the Impact on the Patient Journey and Clinical Care

**DOI:** 10.1155/ijvm/8034303

**Published:** 2026-06-24

**Authors:** Victoria J. Clemett, Jemell Geraghty, Neesha Oozageer Gunowa, Sue Woodward, Tesfamariam Aklilu Betemariam, Audrey Edomobi, Alice Milsom, Toby Prevost, Riana John, Lukla Biasi

**Affiliations:** ^1^ Florence Nightingale Faculty of Nursing, Midwifery & Palliative Care, King′s College London, London, UK, kcl.ac.uk; ^2^ School of Health Sciences, Faculty of Health & Medical Sciences, University of Surrey, Guildford, UK, surrey.ac.uk; ^3^ Faculty of Life Sciences & Medicine, King′s College London, London, UK, kcl.ac.uk; ^4^ Guy′s and St Thomas′ NHS Foundation Trust, London, UK, guysandstthomas.nhs.uk

**Keywords:** health inequities, healthcare disparities, meta-analysis, skin pigmentation, varicose ulcer, varicose veins, venous insufficiency

## Abstract

**Aims:**

This review is aimed at systematically identifying, evaluating and synthesising the influence skin tone has on patient assessment and patient journey in people with chronic venous insufficiency.

**Background:**

Chronic venous disease of the lower limb typically presents with cutaneous manifestations that differ across skin tones. However, its impact on patient assessment and patient journey is unknown.

**Design:**

This systematic review and evidence synthesis were conducted following a protocol prospectively registered in PROSPERO (CRD42023459310) and reported using the PRISMA 2020 guideline.

**Methods:**

Primary research studies, quality improvement projects or case reports published in English up to February 2025 were included. Screening, data extraction and quality appraisal were undertaken independently by two authors. As skin tone was rarely reported, ethnicity or race was used as a proxy, introducing a misclassification risk. Meta‐analysis compared clinical classifications on CEAP, and other data were discussed narratively.

**Data Sources:**

Data sources include MedLine, Excerpta Medica Database (EMBASE), Cumulated Index of Nursing and Allied Health Literature (CINAHL), British Nursing Index (BNI), Scopus and registries of ongoing studies (ISRCTN registry and ClinicalTrials.gov).

**Results:**

Twenty‐two studies were included involving 111,090 individuals. Findings indicate that people of Black and Thai ethnicities may have pathophysiological changes associated with venous disease without some or all the clinical presentations of venous insufficiency. It is hypothesised that this contributes to people with dark skin tones not receiving timely vascular care and appropriate management in the early stages of venous hypertension. However, insufficient reporting on skin tone limits the interpretation of findings.

Evidence suggests treatment disparities across ethnic groups, with people of Black ethnicity having higher treatment costs, more interventions and the least improvements following interventions. However, the contribution of skin tone and clinical presentation remains unclear due to a lack of qualitative data and the influence of other covariables, such as health‐seeking behaviours and compression use.

**Conclusions:**

Visible varicose veins appear to be unreliable indicators of venous disease, which may contribute to diagnostic challenges and health inequity. However, improved skin tone reporting is needed to enable clinical presentation to be considered independently of the social constructs of ethnicity.

## 1. Introduction

Chronic venous insufficiency (CVI) is a spectrum of conditions caused by the dysfunction of the veins and associated pathophysiological consequences [1]. This can lead to venous leg ulceration [2], which affects about 1.1% of the adult population [3]. Individuals with venous leg ulceration often experience debilitating and distressing adverse physical symptoms and negative emotional reactions [4, 5]. Therefore, recent clinical guidelines highlight the importance of primary prevention and early intervention to reduce venous leg ulcer occurrence and reduce venous symptoms [6]. If these vascular changes and cutaneous manifestations associated with venous disease are recognised early, circulation can be improved before an ulcer develops by referral to a vascular specialist and undergoing treatment such as endovenous thermal ablation and adequate compression therapy [7, 8]. Failure in timely diagnosis and treatment results in delayed healing, increased suffering for the patient, increased nursing workload and increased risk of complications [9].

Chronic venous disease presents as cutaneous manifestations that delineate the severity of chronic venous disease, reported as the clinical (C) classification in CEAP [10]. However, the presentation of skin changes may vary across skin tones [11, 12]. A recent review of the global profile of patients with venous leg ulcers identified that ethnicity was rarely reported, and skin tone was not mentioned [13]. Furthermore, there is a lack of guidance to recognise the cutaneous manifestations of CVI in people with dark skin tones in the venous leg ulcer best practice guidance [9] and vascular guidelines [6]. This, combined with a lack of confidence of clinical educators in undertaking assessments of vascular skin changes of the lower limb in people with dark skin tones [14], may explain why 67% of healthcare professionals in the United Kingdom reported no formal training on assessing lower limb skin conditions or infections in people with dark skin tones [15]. This unconscious bias in clinical guidance and healthcare education is likely to lead to unwanted variation in healthcare professionals′ competence in undertaking, recognising and accurately diagnosing vascular changes. But no previous review has examined how skin tone or ethnicity affects CVI and the impact these have on accurate diagnosis, early intervention and patient outcomes, representing a critical evidence gap addressed in this review.

This review is aimed at systematically identifying, evaluating and synthesising the influence skin tone and ethnicity have on patient assessment and patient journey in people with CVI.

It will determine the following:

1. How effective are cutaneous manifestations in identifying CVI in people with dark skin tones?

This will be examined by considering the following:

a. How do cutaneous manifestations correspond with duplex scan results?
b. What is the comparative accuracy of clinical classifications across different skin tone, racial and ethnic groups?
c. What is the interrater and intrarater reliability of assessing skin changes, and is this consistent across different skin tone, racial and ethnic groups?
d. What are the clinicians′ experiences of assessing cutaneous manifestations in people with dark skin tones?

2. What impact do skin tone, race and ethnicity have on the patient journey and clinical care for people with dark skin tones?

This will be examined by considering the following:

a. How does this affect the initial presentation at specialist services?
b. How does this affect the effectiveness of interventions and treatment costs?
c. What are the experiences of patients with dark skin tones in relation to their patient journey and the clinical care they receive?

## 2. Methods

### 2.1. Study Design

This systematic review and evidence synthesis were conducted following a protocol prospectively registered with the PROSPERO database (CRD42023459310) and have been reported following the PRISMA 2020 guideline (File S1).

### 2.2. Search Methods

#### 2.2.1. Eligibility Criteria

##### 2.2.1.1. Types of Studies

Any primary research studies with a research methodology, quality improvement projects or case reports were included in this review. Systematic and narrative reviews, abstracts and conference papers were excluded. Where conference proceedings and registries of ongoing studies were deemed relevant, efforts were made to obtain the full text of these prior to exclusion.

##### 2.2.1.2. Types of Interventions

Any assessment of skin changes includes but is not limited to inspection, palpation and the use of technology. Skin changes associated with CVI were reported using relevant classifications from validated clinical tools where these were available, such as the CEAP classification (File S2) [10] and venous clinical severity scores (VCSSs) (File S3) [16], recommended by ESVS guidelines to group chronic venous disease of the lower limb into subgroups and provide assessments over time, respectively [6].

##### 2.2.1.3. Types of Participants

This review included people across the spectrum of dark skin tones (from very pale brown to deeply pigmented dark brown) living with CVI. As skin tones are poorly reported in the literature, ethnicity or race was used as a proxy to represent skin tone when skin tone was not reported. For this review, the spectrum of dark skin tones was assumed to be represented in people of any ethnicities and races that are not White, East Asian or North Asian. However, it is acknowledged that data obtained from race or ethnicity proxies cause a misclassification risk attributed to analytical comparisons associated with race or ethnicity rather than skin tones.

Populations with light skin tones were only included as a comparison if they were reported independently of people with dark skin tones but were in the same study and recruited from the same population. Papers where data on individuals from groups with dark skin tones could not be separated from groups with light skin tones were excluded.

#### 2.2.2. Information Sources

A systematic search was conducted up to the end of February 2025 of the following databases: MedLine, Excerpta Medica Database (EMBASE), Cumulated Index of Nursing and Allied Health Literature (CINAHL), British Nursing Index (BNI), Scopus and registries of ongoing studies (ISRCTN registry and ClinicalTrials.gov).

#### 2.2.3. Search Strategy

The search was conducted based on a population/intervention/outcome framework. The population terms examined individuals with CVI who had dark skin tones (which includes cases where ethnicity or race is reported without reporting skin tone specifically). The intervention terms relate to the assessment of skin changes associated with chronic venous disease (e.g. ‘Skin assessment’, ‘Tissue assessment’, ‘Nursing assessment’, ‘Physical assessment’, ‘Vascular assessment’, ‘Lower limb assessment’, ‘ABPI’, ‘Duplex’, ‘CEAP’ and ‘Doppler’). The outcome terms relate to effectiveness and impact (e.g. ‘Accuracy’, ‘Efficacy’, ‘Effectiveness’, ‘Validity’, ‘Reliability’, ‘Differential diagnosis’, ‘Diagnosis’, ‘Timely diagnosis’, ‘Prompt diagnosis’, ‘Early diagnosis’, ‘Delayed diagnosis’, ‘Referral’, ‘Patient care’, ‘Patient journey’, ‘Patient trajectory’, ‘Patient outcomes’ and ‘Clinical outcomes’). These criteria were used across all databases using MeSH terms and keyword searches. The detailed search strategy is reported in File S4.

#### 2.2.4. Selection Process

Two reviewers (VC and RJ or VC and SW) independently screened the titles and abstracts for eligibility within Covidence (Covidence systematic review software, http://www.covidence.org). The reviewers met to discuss any disagreements and agreed on which articles would progress to full‐text screening. Two reviewers (JG and NOG, JG and VC or NOG and VC) then independently examined the full‐text articles within Covidence to determine inclusion against the review question and inclusion/exclusion criteria. Throughout this process, disagreements about eligibility were resolved through discussion and, if required, by bringing in a third reviewer.

#### 2.2.5. Data Abstraction

##### 2.2.5.1. Data Collection Process

Two reviewers (VC and TAB, VC and AM or VC and AE) independently extracted data from the included articles in Covidence and met to resolve discrepancies in data collection. Study data were obtained on study characteristics, population characteristics and findings. Data were stratified by ethnicity or skin tone data (where available). These groups consisted of the following ethnic groups: ‘Asian‐east’, ‘Asian‐south’, ‘Asian‐southeast’, ‘Asian‐western & central’ (e.g. Arab), ‘Black’ (e.g. African, Caribbean, African American and Black British), ‘Hispanic & Latino’ (e.g. Central and South American) and ‘Other ethnic group with dark skin tones’ (which was stated within the data extraction). Where data on patients with the light skin tones were recorded for comparison, this was defined as ‘White’ (e.g. White American and White European), ‘North Asian’ or ‘East‐Asian’ (e.g. Japanese, Chinese and Korean).

##### 2.2.5.2. Data Items

Study data were obtained on study characteristics and population characteristics (Table 1). Participant characteristics were reported by skin tone/ethnicity independently. This included data on age, gender, body mass index and relevant comorbidities or past medical history (history of DVTs, coronary arterial disease, diabetes, hypertension, pregnancy, lower leg trauma and smoking).

**Table 1:** Study characteristics and key findings.

Author (date), country	Study Design	Inclusion	No.		Groups of people across the spectrum of dark skin tones					Other groups included	Follow‐up	Key findings	Reporting quality
					Asian (S)	Asian (SE)	Black	Hispanic/Latino	Other dark skin tone groups				
Alsheekh et al. [17], United States	Cohort study	Underwent iliac vein stent	623	Number ($\begin{array}{c} n \end{array}$)			89	138		White ($\begin{array}{c} n=369 \end{array}$), not defined ($\begin{array}{c} n=23 \end{array}$)	1 year	No differences in presenting signs on C3–C6 CEAP classification between ethnic groups. However, some nonstatistical trends emerged: For C3, $\begin{array}{c} \text{Black}=55.1\% \end{array}$, $\begin{array}{c} \text{Hispanic}=59.4\% \end{array}$ and $\begin{array}{c} \text{White}=50.5\% \end{array}$, and for C4, $\begin{array}{c} \text{Black}=22.5\% \end{array}$, $\begin{array}{c} \text{Hispanic}=28.3\% \end{array}$ and $\begin{array}{c} \text{White}=29.3\%. \end{array}$ Active venous ulcers were observed in: $\begin{array}{c} \text{Black}=1189/ \end{array}$ (10.1%), $\begin{array}{c} \text{Hispanic}=12138/ \end{array}$ (8.7%) and $\begin{array}{c} \text{White}=45369/ \end{array}$ (20.2%). No difference in degree of iliac vein stenosis between the ethnic groups ($\begin{array}{c} \text{Black}=64.9\% \end{array}$ SD 13, $\begin{array}{c} \text{Hispanic}=66.613.6\%\pm \% \end{array}$ and $\begin{array}{c} \text{White}=64.3\% \end{array}$ SD 14.1). Race/ethnicity is not correlated with thrombosis of the iliac vein after stenting.	Poor
				Age ($\begin{array}{c} \text{av}\pm \text{SD} \end{array}$)			$\begin{array}{c} 63.614\pm \end{array}$	$\begin{array}{c} 6314\pm \end{array}$					
				$\begin{array}{c} \%F \end{array}$ ($\begin{array}{c} n \end{array}$, %)			67 (75.3%)	101 (73.2%)					

Balasubramanyam et al. [18], United States	Case report	n/a—case report	1	Number ($\begin{array}{c} n \end{array}$)			1			n/a	1 year	Venous leg symptoms and lipodermatosclerotic changes improved after treatment.	Good
				Age ($\begin{array}{c} \text{av}\pm \text{SD} \end{array}$)			72						
				$\begin{array}{c} \%F \end{array}$ ($\begin{array}{c} n \end{array}$, %)			Female						

Bai et al. [19], United States	Cross‐sectional study	EHR of patients with chronic (PVOO) secondary to NIVLs	840	Number ($\begin{array}{c} n \end{array}$)			44	59		Asian (U) ($\begin{array}{c} n=645 \end{array}$), White ($\begin{array}{c} n=60 \end{array}$)	n/a	There is a higher proportion of people of Black ethnicity with C0–C2 skin changes (Black: 4/44 [9%], Hispanic: 2/59 [3.4%] and White: 3/60 [5%]). But how many have C0 skin changes is unreported. People of White ethnicity have less leg oedema C3 (43.3%) compared to other ethnic groups (Asian $\begin{array}{c} \left[\text{U}\right]=57.8\% \end{array}$, $\begin{array}{c} \text{Black}=47.7\% \end{array}$ and $\begin{array}{c} \text{Hispanic}=64.4\% \end{array}$), and Asian (U) populations have more C4 skin changes (27.1%) compared to Black (15.9%), Hispanic (11.8%) and White (15%). Previous & active venous ulcers (C5 & C6) were reported in Asian $\begin{array}{c} \left(\text{U}\right)=45645/ \end{array}$ (7.0%), $\begin{array}{c} \text{Black}=1244/ \end{array}$ (27.3%), $\begin{array}{c} \text{Hispanic}=1259/ \end{array}$ (20.3%) and $\begin{array}{c} \text{White}=2260/ \end{array}$ (36.7%).	Good
				Age ($\begin{array}{c} \text{av}\pm \text{SD} \end{array}$)			—	—					
				*%* *F* ($\begin{array}{c} n \end{array}$, %)			—	—					

Cho et al. [20], United States	Cohort study	Underwent endovascular placement of an iliofemoral vein stent	827	Number ($\begin{array}{c} n \end{array}$)			47	60		Asian (U) ($\begin{array}{c} n=653 \end{array}$), White ($\begin{array}{c} n=67 \end{array}$)	Up to 7 years (av. ~2 years)	People of Asian (unknown) ethnicity present with significantly more severe varicose veins (VCSS $\begin{array}{c} 1.41.0\pm \end{array}$). However, rates between other ethnic groups are similar (Black $\begin{array}{c} 0.70.9\pm \end{array}$, Hispanic $\begin{array}{c} 1.01.0\pm \end{array}$ and White $\begin{array}{c} 0.81.0\pm \end{array}$). There were no significant differences between ethnic groups in the severity of C3 (oedema) & C4 (pigmentation) on VCSS preintervention. Pain preintervention was significantly different across ethnic groups on VCSS (Asian [U] $\begin{array}{c} 1.70.9\pm \end{array}$, Black $\begin{array}{c} 1.91\pm \end{array}$, Hispanic $\begin{array}{c} 2.2038\pm \end{array}$ and White $\begin{array}{c} 1.80.9\pm \end{array}$, $\begin{array}{c} p<0.001 \end{array}$). Black patients had the most reinterventions and were most likely to undergo a major reintervention, while Asian patients had the fewest (Black $\begin{array}{c} 2.61.9\pm \end{array}$ vs. Asian [U] $\begin{array}{c} 1.71.1\pm \end{array}$, Hispanic $\begin{array}{c} 2.41.9\pm \end{array}$ and White $\begin{array}{c} 2.21.5\pm \end{array}$). No differences in outcomes were observed as defined by changes in VCSS and changes in CAS. At 3 years post op, people of White ethnicity had the greatest change in VCSS $\begin{array}{c} 9.66.5\pm \end{array}$ vs. Asian (U) $\begin{array}{c} 4.13.7\pm \end{array}$, Black $\begin{array}{c} 4.43.0\pm \end{array}$ and Hispanic $\begin{array}{c} 1.44.6\pm \end{array}$ ($\begin{array}{c} p<0.001 \end{array}$).	Good
				Age ($\begin{array}{c} \text{av}\pm \text{SD} \end{array}$)			$\begin{array}{c} 61.713.0\pm \end{array}$	$\begin{array}{c} 62.514.4\pm \end{array}$					
				*%* *F* ($\begin{array}{c} n \end{array}$, %)			37 (78.7%)	41 (68.3%)					

Criqui et al. [21], United States	Cross‐sectional study	Nonclinical population	2211	Number ($\begin{array}{c} n \end{array}$)			318 (14.4%)	338 (15.3%)		Asian (U) ($\begin{array}{c} n=273 \end{array}$), White ($\begin{array}{c} n=1282 \end{array}$)	n/a	Compared with non‐Hispanic Whites, minority groups in general had lower prevalences of both visible and functional disease. Visible and functional disease were closely related. OR of having telangiectasias was significantly lower than the White reference group for Blacks (OR: 0.33, $\begin{array}{c} p<0.05 \end{array}$), Hispanics (OR: 0.66, $\begin{array}{c} p<0.05 \end{array}$) and Asian ($\begin{array}{c} \text{U} \end{array}$) (OR: 0.36, $\begin{array}{c} p<0.05 \end{array}$).	Good
				Age ($\begin{array}{c} \text{av}\pm \text{SD} \end{array}$)			—	—					
				*%* *F* ($\begin{array}{c} n \end{array}$, %)			—	—					

Danielsson et al. [22], United States	Cross‐sectional study	Patient with CVD at the vascular centre clinic and hospital	272 (401 limbs)	Number ($\begin{array}{c} n \end{array}$)		32 limbs			Pacific Islander: 37 legs	Asian (mainly E) (120 limbs), White (172 limbs), mixed or not defined (40 limbs)	n/a	Skin changes (C4) differed across ethnic groups, with the lowest frequency observed among SE Asian (Filipinos) (15.6%) and the highest among Pacific Islanders (56.8%). Asian $\begin{array}{c} \left(\text{mainly}\;\text{E}\right)=32.5\% \end{array}$ and $\begin{array}{c} \text{White}=30.2\% \end{array}$. However, when adjusted for age, sex and BMI, there was no difference across groups.	Good
				Age ($\begin{array}{c} \text{av}\pm \text{SD} \end{array}$)		49			55				
				*%* *F* ($\begin{array}{c} n \end{array}$, %)		—			—				

De Souza et al. [23], Brazil	Cross‐sectional study	Patient seen at the vascular surgery outpatient clinic with clinical signs of CVD & active ulcer (C6)	154	Number ($\begin{array}{c} n \end{array}$)			82 (53.2%)		Brown: 63 (40.9%)	White ($\begin{array}{c} n=9 \end{array}$)	n/a	Venous ulcers occurred in people with low income, ethnically Black or Brown women with a low education level, who work in the informal sector or receive welfare support. Mean age 53 years, indicating the populations in this study experience early onset CVI. There was a predominance in people with dark skin tones seeking help for CVI (Black 53.2% and Brown 40.9%), but the author states this may be explained by the ethnic diversity of the local population.	Good
				Age ($\begin{array}{c} \text{av}\pm \text{SD} \end{array}$)			—		—				
				$\begin{array}{c} \%F \end{array}$ ($\begin{array}{c} n \end{array}$, %)			—		—				

Dua et al. [24], United States	Cross‐sectional study	Patients with diagnosis codes for venous stasis with ulceration (454.0) or complications (454.2) on EHR	20,648	Number ($\begin{array}{c} n \end{array}$)			2554			White ($\begin{array}{c} n=12,500 \end{array}$)	n/a	People who were of Black ethnicity were significantly more likely to present with advanced chronic venous insufficiency with CEAP scores of 5 or 6 at a younger age than their Caucasian counterparts (age by ethnicity of people with C5 & C6: Black $\begin{array}{c} 61.516.7\pm \end{array}$ and White $\begin{array}{c} 68.215.8\pm \end{array}$, $\begin{array}{c} p<0.001 \end{array}$). Caucasian patients were more likely to undergo vein stripping and/or ligation compared with African American patients (Black 6.3% vs. White 11.4%), indicating that perhaps African American patients may not be undergoing aggressive management of their venous disease early. The African American race is associated with increased ulcer debridement (Black: 17.8% vs. White 6.5%, $\begin{array}{c} p<0.001 \end{array}$), DVT rates (Black 7.9% vs. White 6.5%, $\begin{array}{c} p<0.001 \end{array}$) and hospital costs (Black $7116 vs. White $6281, $\begin{array}{c} p<0.001 \end{array}$).	Good
				Age ($\begin{array}{c} \text{av}\pm \text{SD} \end{array}$)			$\begin{array}{c} 61.516.7\pm \end{array}$						
				*%* *F* ($\begin{array}{c} n \end{array}$, %)			1285 (50.3%)						

Fronek et al. [25], United States	Cross‐sectional study	Nonclinical population	2408 (3549 limbs)	Number ($\begin{array}{c} n \end{array}$)			Unreported	Unreported		Asian (U), White N unreported	n/a	The augmentation responses varied across ethic groups. These were generally lower if venous obstruction was accompanied by trophic changes.	Outstanding
				Age ($\begin{array}{c} \text{av}\pm \text{SD} \end{array}$)			—	—					
				*%* *F* ($\begin{array}{c} n \end{array}$, %)			—	—					

Kanchanabat et al. [26], Thailand	Cross‐sectional study	Any patient seen at the vascular surgery outpatient clinic with clinical signs of CVI (CEAP 4–6)		Number ($\begin{array}{c} n \end{array}$)		41 (58 limbs)				n/a	n/a	This study suggested BMI, leg trauma and standing posture as the possible contributing factors to the development of CVI. Varicose veins were evident in 50% (24/48 limbs) of those with known CVI compared to 22% (18/82 limbs) in age‐matched controls ($\begin{array}{c} p=0.01 \end{array}$). Duplex ultrasonography revealed great saphenous reflux in 58% of the legs without visible varicose veins. The prevalence of deep vein reflux in 48 legs with CEAP Classifications 4, 5 and 6 were 95%, 88% and 82%, respectively. Patients tend to be younger and have less severe associated symptoms in this study compared to others. Authors suggest that aetiologies may differ across ethnic groups.	Outstanding
			41 (58 limbs)	Age ($\begin{array}{c} \text{av}\pm \text{SD} \end{array}$)		$\begin{array}{c} 5812\pm \end{array}$							
				*%* *F* ($\begin{array}{c} n \end{array}$, %)		23 (56.1%)							
		Age‐matched control recruited from the same hospital (nonvascular patients)	41	Number ($\begin{array}{c} n \end{array}$)		41							
				Age ($\begin{array}{c} \text{av}\pm \text{SD} \end{array}$)		58 ± 12							
				*%* *F* ($\begin{array}{c} n \end{array}$, %)		21 (51.22%)							

Kanchanabat et al. [27], Thailand	Cross‐sectional study	Patient seen at the vascular surgery outpatient clinic with clinical signs CVI (CEAP 4–6)	78 (102 limbs)	Number ($\begin{array}{c} n \end{array}$)		78 (102 legs)				n/a	n/a	The prevalence of deep vein reflux is high and often with superficial vein reflux. Visible varices are infrequent. Great saphenous reflux was present in 68.8% of the legs without visible varicose veins (VCSS 0). In the legs with superficial vein reflux (SVR), obvious varicosity (VCSSs 2 and 3) was found in only 21 legs (29.2%). Half (50%) of the CVI legs were painless. Forty‐two per cent of the patients spend more than 75% of their working time walking and standing.	Poor
				Age ($\begin{array}{c} \text{av}\pm \text{SD} \end{array}$)		$\begin{array}{c} 59.612.5\pm \end{array}$							
				$\begin{array}{c} \%F \end{array}$ ($\begin{array}{c} n \end{array}$, %)		43 (55.1%)							

Kanchanabat & Stapanavatr [28], Thailand	Cross‐sectional study	Patient seen at the vascular surgery outpatient clinic with clinical signs CVI (CEAP 4–6)	79 (104 limbs)	Number ($\begin{array}{c} n \end{array}$)		79 (104 limbs).				n/a	n/a	Visible varicose is infrequent, but most of those veins are associated with reflux. Prevalence of deep vein reflux in the C4, C5 and C6 legs was 71.0% (22/31), 60.0% (6/10) and 59.2% (29/49), respectively. Calf GSV or SSV reflux was the most common finding among patients with recurrent or nonhealed ulcerations.	Good
				Age ($\begin{array}{c} \text{av}\pm \text{SD} \end{array}$)		$\begin{array}{c} 59.812.5\pm \end{array}$							
				*%* *F* ($\begin{array}{c} n \end{array}$, %)		43 (54.4%)							

Kiguchi et al. [29], United States	Cross‐sectional study	Underwent endovenous closure of their saphenous veins	2346	Number ($\begin{array}{c} n \end{array}$)			196	511		Asian (unknown) ($\begin{array}{c} n=234 \end{array}$), White ($\begin{array}{c} n=1299 \end{array}$) or not defined ($\begin{array}{c} n=106 \end{array}$)	n/a	People of Black ethnicity conferred a higher risk of presenting with an advanced CEAP class compared with other races (active ulcers [C6] in Asian [U] 6.4%, Black 28.4%, Hispanic 12.7% and White 15.6%). Black race, increasing age, male sex and a history of DVT are all predictive of an advanced CEAP class on initial presentation. People from the Black ethnicity had significantly fewer varicose veins (8.8%) compared to Asian (U) (52.4%), Hispanic (34.4%), White (21.9%) and not‐defined ethnic group (22.6%) ($\begin{array}{c} p<0.001 \end{array}$). People of Asian (U) ethnicity had less oedema and C4 skin changes than other ethnicities ($\begin{array}{c} \text{oedema}=21.9\% \end{array}$ vs. Black 37.1%, Hispanic 33.5% and White 40.8%; C4 pigmentation $\begin{array}{c} \text{changes}=16.7\% \end{array}$ vs. Black 17.9%, Hispanic 18.6% and White 18.1%).	Good
				Age ($\begin{array}{c} \text{av}\pm \text{SD} \end{array}$)			—	—					
				$\begin{array}{c} \%F \end{array}$ ($\begin{array}{c} n \end{array}$, %)			—	—					

Murli et al. [30], Malaysia	Cohort study	All patients with venous ulcers (CEAP 5 or 6) and saphenofemoral junction (SFJ) or saphenopopliteal junction (SPJ) reflux who visited the surgical outpatient clinic	123 (145 limbs)	Number ($\begin{array}{c} n \end{array}$)	51 limbs	9 limbs					Asian (East) ($\begin{array}{c} n=84 \end{array}$ limbs), not defined (1 limb)	Ethnic differences in BMI and occupation influenced the outcomes and recurrence rates of venous ulcers. The study underscores the importance of holistic management in treating venous ulcers. Reasons to seek treatment were due to the symptomatology of pain (98.6%), swelling (83.1%), cramps (69.7%), heaviness (66.4%), cellulitis (23.4%), superficial thrombophlebitis (22.8%) or bleeding venous ulcers (19.3%).	Poor
				Age ($\begin{array}{c} \text{av}\pm \text{SD} \end{array}$)	—	—							
				$\begin{array}{c} \%F \end{array}$ ($\begin{array}{c} n \end{array}$, %)	25 (49.0%)	2 (22.2%)							

Langer et al. [31], United States	Cross‐sectional study	Nonclinical population	600	Number ($\begin{array}{c} n \end{array}$)			150	150		Asian (unknown) ($\begin{array}{c} n=150 \end{array}$), White ($\begin{array}{c} n=150 \end{array}$)	n/a	There were only consistent ethnic differences for telangiectasis, which were more common in Caucasians. Significant differences in OR for Black (male OR 0.37, $\begin{array}{c} p=0.01 \end{array}$, and female OR 0.12, $\begin{array}{c} p<0.01 \end{array}$) and Asian (U) females (OR 0.19, $\begin{array}{c} p=0.01 \end{array}$). Varicose veins, SVD (superficial venous disease) and DVD (deep venous disease) did not differ across ethnic groups.	Outstanding
				Age ($\begin{array}{c} \text{av}\pm \text{SD} \end{array}$)			M: $\begin{array}{c} 5710\pm \end{array}$	M: $\begin{array}{c} 5411\pm \end{array}$					
							F: $\begin{array}{c} 5711\pm \end{array}$	F: $\begin{array}{c} 5812\pm \end{array}$					
				$\begin{array}{c} \%F \end{array}$ ($\begin{array}{c} n \end{array}$, %)			75 (50%)	75 (50%)					

Pappas et al. [32], United States	Cohort study	EHR of patients presenting for CVD evaluation at vein restoration centres across 14 states	66,621	Number ($\begin{array}{c} n \end{array}$)			~11,326	~11,992		Asian (unknown) ($\begin{array}{c} n \end{array}$ ~1997), White ($\begin{array}{c} n \end{array}$ ~366412) or other ($\begin{array}{c} n \end{array}$ ~5330)	1 month	The proportion of sign and symptom prevalence was greatest in Whites for all categories. C2 skin changes are less prevalent in African Americans compared to other ethnicities (Black: 12% vs. Asian [U] 25%, Hispanic 30%, White 22% and others 22%). Treatment outcomes as assessed by rVCSS similarly differ by race. African Americans often require more ablations, phlebectomies and ultrasound‐guided foam injections to achieve the same degree of improvement as other races.	Poor
				Age ($\begin{array}{c} \text{av}\pm \text{SD} \end{array}$)			—	—					
				$\begin{array}{c} \%F \end{array}$ ($\begin{array}{c} n \end{array}$, %)			79%	85%					

Paul et al. [33], United States	Cross‐sectional study	From 12 methadone treatment clinics	161	Number ($\begin{array}{c} n \end{array}$)			113 (70.2%)			Not stated ($\begin{array}{c} n=48 \end{array}$)	n/a	A higher proportion of African Americans experienced itchy legs/feet (75.58% of people with itch were African American, but the population made up only 70.19% of the total sample). This was greater than the proportion who experienced wound itch (71.43% of those who had wound itch were African American).	Excellent
				Age ($\begin{array}{c} \text{av}\pm \text{SD} \end{array}$)			—						
				$\begin{array}{c} \%F \end{array}$ ($\begin{array}{c} n \end{array}$, %)			—						

Pinto Rodríguez et al. [34], United States	Cohort study	EHR of patients undergoing vein ablation (VA) for C2 to C4 disease	3544	Number ($\begin{array}{c} n \end{array}$)			$\begin{array}{c} \text{VCSS}=101 \end{array}$	$\begin{array}{c} \text{VCSS}=134 \end{array}$		White ($\begin{array}{c} \text{VCSS}=2065 \end{array}$/$\begin{array}{c} \text{VVSymQ}=2254 \end{array}$), other ($\begin{array}{c} \text{VCSS}=441 \end{array}$/$\begin{array}{c} \text{VVSymQ}=473 \end{array}$)	Between 2 weeks & 6 months	People of Black ethnicity lack clinical improvement in 24.75% compared to Latino 14.18% and White 16.71% ($\begin{array}{c} p=0.001 \end{array}$). Regression analysis of factors independently associated with lack of clinical improvement (LCI) based on venous clinical severity score (VCSS) indicate that race/ethnicity is not an independent risk factor. LCI after vein ablation (VA) is associated with treating patients with a less severe CEAP clinical classification (C2) and the lack of compression therapy before intervention.	Good
							$\begin{array}{c} \text{VVSymQ}=114 \end{array}$	$\begin{array}{c} \text{VVSymQ}=133 \end{array}$					
				Age ($\begin{array}{c} \text{av}\pm \text{SD} \end{array}$)			—	—					
				*%* *F* ($\begin{array}{c} n \end{array}$, %)			—	—					

Taengsakul [35], Thailand	Cross‐sectional study	Diagnosed with CVD	260	Number ($\begin{array}{c} n \end{array}$)		260				n/a	n/a	The major risk factors for severe CVD are a history of DVT, high BMI, diabetes mellitus and hypertension. The most common CEAP classification was C2 (39%). An increasing number of venous systems involved increased the risk of severe CVD. Perforator reflux significantly differed between those with C0–C2 compared to C4–C6 classification (33.6% vs. 74.4%, $\begin{array}{c} p<0.001 \end{array}$). There was a nonsignificant trend for more deep vein reflux (18.6% vs. 10%) and more superficial vein reflux in those with C4–C6 disease compared to C0–C2.	Excellent
				Age (av ± SD)		61.9 ± 12.8							
				*%* *F* ($\begin{array}{c} n \end{array}$, %)		200 (76.9%).							

Taofan et al. [36], Indonesia	Case series	Patients with active VLU undergoing EVLA		Number ($\begin{array}{c} n \end{array}$)		3				n/a	6 months	Ulcer healing: All ulcers showed significant healing by 6 months. VCSS improvement: Reduction by 14–17 points. Complications: No recanalisation or nerve injury on duplex ultrasound (DUS).	Poor
			3	Age ($\begin{array}{c} \text{av}\pm \text{SD} \end{array}$)		$\begin{array}{c} 58.37.6\pm \end{array}$							
				$\begin{array}{c} \%F \end{array}$ ($\begin{array}{c} n \end{array}$, %)		1 (33.3%)							

Verma et al. [37], India	Cross‐sectional study	Patients with symptomatic varicose veins (C2–C6 only)	200	Number ($\begin{array}{c} n \end{array}$)	200					n/a	n/a	Weight and BMI were significantly positively correlated with the diameter of competent perforators ($\begin{array}{c} r=0.500 \end{array}$, $\begin{array}{c} p<0.001 \end{array}$ and $\begin{array}{c} r=-0.339 \end{array}$, $\begin{array}{c} p=0.021 \end{array}$, respectively). Other anatomical changes, such as the diameter of GSV and SSV, were not significantly correlated with age, weight and BMI.	Excellent
				Age ($\begin{array}{c} \text{av}\pm \text{SD} \end{array}$)	$\begin{array}{c} 35.659.91\pm \end{array}$								
				$\begin{array}{c} \%F \end{array}$ ($\begin{array}{c} n \end{array}$, %)	36 (18%)								

Zil‐E‐Ali et al. [38], United States	Cohort study	HER of patients undergoing vein ablation (VA) for CEAP C2–C4 disease	9009	Number ($\begin{array}{c} n \end{array}$)			490	627		White ($\begin{array}{c} n=7892 \end{array}$)	Unclear >1 year on EHR. Follow‐up visit 67.9% within 30 days.	Differences exist in the clinical severity and symptom presentation based on race. People from the Black ethnicity had fewer varicose veins (11%) compared to the Hispanic (25.5%) and White (23.1%) groups. However, C4 skin changes were similar across ethnic groups (Black: 21%, Hispanic: 22.5% and White: 20.2%) Black/African American patients present with more advanced CVI than do their White and Hispanic counterparts. Furthermore, the postprocedural analysis of the Black/African American cohort showed inferior clinical and self‐reported improvement in their CVI. Black: B $\begin{array}{c} \text{coefficient}=0.72 \end{array}$ (−1.10, −0.34 95% CI), $\begin{array}{c} p<0.001 \end{array}$, and Hispanic: B $\begin{array}{c} \text{coefficient}=0.29 \end{array}$ (−0.04, 0.64 95% CI), $\begin{array}{c} p=0.84 \end{array}$ compared to White reference. Although the Hispanic population was younger, the White and Hispanic patients experienced similar responses to treatment.	Good
				Age ($\begin{array}{c} \text{av}\pm \text{SD} \end{array}$)			$\begin{array}{c} 55.9612\pm \end{array}$	$\begin{array}{c} 51.6713\pm \end{array}$					
				$\begin{array}{c} \%F \end{array}$ ($\begin{array}{c} n \end{array}$, %)			319 (65.1%)	448 (71.5%)					

Data were extracted to compare skin changes to imaging data (e.g. duplex ultrasound and venographs), clinical signs or symptoms, interrater reliability and intrarater reliability. Longitudinal outcomes were sought on symptoms and function, treatment provided and costs and skin changes. Where reported data on validated tools, CEAP (clinical classifications) and VCSSs were extracted.

##### 2.2.5.3. Effect Measures

Where available, the number of events on CEAP classification was extracted or calculated from data (e.g. calculated from the percentage [%] of people with this classification if not presented). VCSSs were extracted from both baseline data and outcome data for each component of the VCSS. Other data were extracted as provided in the articles.

#### 2.2.6. Quality Appraisal

Two reviewers (VC and TAB, VC and AM or VC and AE) independently rated the quality of each article using the Joanna Briggs critical appraisal tools relevant to the study type (https://jbi.global/critical-appraisal-tools) and met to resolve discrepancies in quality appraisal. These responses were translated into a quality of reporting rating by calculating the percentage of positive responses (e.g. answering ‘yes’) to the questions on the Jonna Briggs critical appraisal tools. These were classified as outstanding (100%), excellent (74%–99%), good (50%–74%) and poor (less than 50%) reporting standards. No study was excluded based on the quality of the research; however, more weight was given to findings of studies with greater methodological rigour. Furthermore, both a meta‐analysis of all included studies and a sensitivity analysis excluding studies rated as having a poor reporting quality were undertaken to evaluate the robustness of the pooled effect.

#### 2.2.7. Synthesis Methods

Data were synthesised by findings related to the primary outcome (effectiveness of skin changes to determine CVI) and secondary outcomes (impact on patient journey and clinical care).

Several studies reported skin changes as binary data (number or percentage of observations) on the CEAP classification tool for two subgroups of people: individuals with dark skin tones (of Black ethnicity) and individuals with light skin tones (of White ethnicity) at a single time point (either in cross‐sectional studies or reported data at single time point, e.g. on presentation, for cohort studies) recruited from the same population (e.g. patients undergoing the same vascular procedure). This indicated that meta‐analysis could be undertaken. Data were converted to the number of events (people in each group with this clinical classification) and nonevents (total people in this group without this clinical classification) for each group. Subgroup analysis separated the groups into (a) nonclinical groups, (b) those referred to vascular services (e.g. those with and without a defined diagnosis) and (c) those with known venous disease (e.g. those requiring intervention). Meta‐analysis was undertaken in STATA (Version 18) to calculate the risk ratio. This used a random‐effects model (REML method). *I* ^2^ was calculated and interpreted as 25% low, 50% moderate and 75% high between‐study heterogeneity. Data for meta‐analysis was calculated by VC and checked by either AM or AE. Data was analysed by VC in STATA with statistical advice provided by TP.

Other findings were synthesised narratively due to variations between studies, interventions and/or outcome measures.

#### 2.2.8. Reporting Bias Assessment and Certainty Assessment

Reporting bias determined whether any findings of interest were stated in the method but then not reported in the findings or only partially reported in the findings. The GRADE reporting system [39] pooled the results to determine the overall quality of evidence.

## 3. Results

### 3.1. Study Selection and Study Characteristics

**Figure 1:**
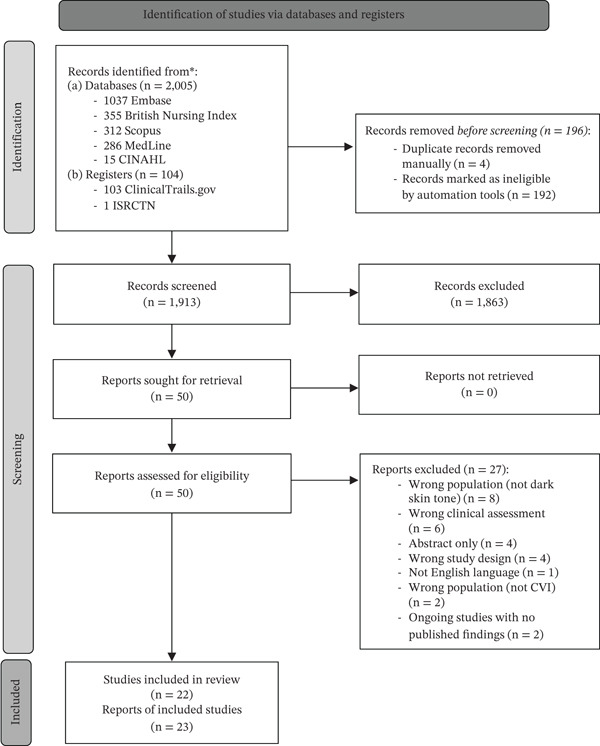
PRISMA flow chart.

Initial search obtained 1911 studies after removing duplicates (Figure 1). After screening titles, abstracts and full texts for eligibility, 22 articles were included in the review (Figure 1). This consisted of 5219 individuals across three studies recruited from nonclinical populations [21, 25, 31] and 39,048 of individuals across 17 studies with venous disease [17–20, 22–24, 26–30, 32, 34–38], of which one study also matched their clinical population to an age‐matched control group of 41 people recruited from the same hospital [26]. Additional studies recruited 66,621 people referred to the specialist vascular team, where 20,644 (31.0%) were treated for CVI of the lower limb [32], and 161 people from a methadone clinic, where only 1.9% of participants did not show any signs of chronic venous disease (Table 1) [33]. Furthermore, there were two studies that may produce relevant results for our review (NCT06798766: ‘Reducing Skin Tone Inequities in Chronic Venous Insufficiency’ and NCT06683586: ‘Brazilian Registry of Chronic Venous Disease ‐ Risk Factors, Comorbidities, Clinical and Surgical Treatment’). However, these were classed as ongoing with no published research data available at the time of writing.

Fourteen studies were conducted in the United States [17–22, 24, 25, 29, 31–34, 38], four in Thailand [26–28, 35] and one each from Brazil [23], India [37], Indonesia [36] and Maylasia [30] (Table 1).

Included studies involved people from a range of ethnic groups, representing people across a spectrum of dark skin tones (Table 1). This consisted of 14 studies involving people of Black ethnicity [17–21, 23–25, 29, 31–34, 38], 10 studies involving Hispanic people [17, 21, 25, 29, 31, 32, 34, 38] and seven studies involving Southeast Asian populations. Pacific Islanders were involved in one study [22], and South Asian populations were involved in two studies [30, 37] (Table 1).

People with light skin tones were considered alongside other populations with dark skin tones in two studies involving East Asian populations [22, 30] and 13 involving people from ethnically White populations [17, 20–25, 29, 31, 32]. Populations expected to include a spectrum of light and dark skin tones were also mentioned in several studies, including seven studies reporting an unspecified Asian population [20, 21, 25, 29, 31, 32] and another eight studies reporting an unspecified other or mixed ethnicity population (Table 1).

Only one study set in Brazil directly reported skin tone characterising people as black, brown and white [23]. There was no reference to any validated tool to classify skin tone in any study.

### 3.2. Risk of Bias in Studies

The overall risk of bias varied, but most studies lacked clarity in the reproducibility of measuring the cutaneous manifestations of CVI by the researchers and clinicians. However, both chronic venous disease and outcomes are generally measured using standardised tools such as VCSS for condition severity and CEAP to delineate presentation of lower limb chronic venous disease. Additionally, researchers often made attempts to minimise the risk of bias in measuring the condition, especially within the cross‐sectional studies of nonclinical populations and some patient groups with known venous disease, by using duplex USS to define CVI (File S5).

### 3.3. Results on Primary Outcome: Effectiveness of Skin Changes to Determine CVI

No eligible studies used a validated tool to measure skin tone, meaning the effectiveness of skin changes for people with dark skin tones to identify CVI could not be directly evaluated. Consequently, any assessment of the diagnostic value of skin changes was limited by findings that relied on ethnic groups.

#### 3.3.1. Triangulation of Data to Scan Results

Triangulation of data from duplex ultrasound results against skin changes occurred in five studies involving participants from South Asia and Southeast Asia [26–28, 35, 37]. Findings indicate that, in these populations, cutaneous manifestations of venous disease are associated with anatomical (A) and pathophysiological (P) classifications, evidenced by a lower incidence of vein reflux in those with C0–C3 compared to C4–C6 clinical classifications [35] and a high proportion of deep vein reflux in those with C4 skin changes [26, 28]. There is also an indication that overall VCSSs are statistically significantly correlated with incompetence in the veins within South Asian populations [37]. However, skin changes are only part of the composite VCSS (File S3). Therefore, it is unclear what contribution skin changes have to this finding. Conversely, in patients with a C4–C6 clinical classification of venous disease, there is a high proportion without visible varicose veins when great saphenous reflux is present [26, 27] and when superficial vein reflux is present (70.8%) [27].

#### 3.3.2. Comparison of Clinical Classification Across Ethnicities and Racial Groups

Further evaluation of the effectiveness of skin changes to identify chronic venous disease relied on inferring diagnostic accuracy by making comparisons in clinical classification between ethnic and racial groups obtained from the same population.

**Figure 2:**
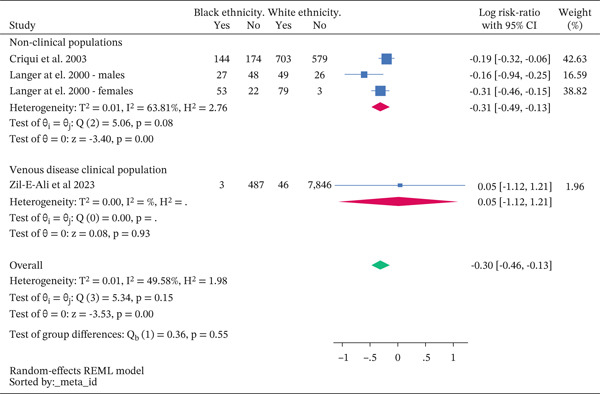
Meta‐analysis of the proportion of people with C1 between dark (Black ethnicity) and light (White ethnicity) skin tones. Notes: (1) This was reported as Telangiectasis (spider veins) by Criqui et al. [21] and Langer et al. [31]. This was reported as CEAP C1 by Zil‐E‐Ali et al. [38] (where the highest classification on CEAP is reported). (2) Data from articles with poor reporting quality [32] have been removed. All data available in Supporting Information S6.

Analysis supports that some clinical (C) classifications of venous disease may be less readily observed in some ethnic groups. Studies in nonclinical populations found that ethnicity was a predictor of telangiectasis (C1), with populations of Black ethnicity being 26.6% less likely to have telangiectasia than their counterparts of White ethnicity (RR = 0.73 [0.61, 0.88], *z* = −3.4, *p* < 0.01) (Figure 2). These studies were both of outstanding or excellent reporting quality (File S5). However, even though all studies had the same trend, there is a moderate between‐study heterogeneity (*I* ^2^ = 63.8*%*) and a wide 95% prediction interval, demonstrating variation in the proportions of skin changes observed across studies.

However, within clinical groups, the proportion of those with C1 skin changes was similar between White ethnicity and Black ethnicity [32, 38]. When combined with nonpatient populations, the true effect size in 95% of comparable populations falls in a very wide prediction interval of RR = 0.30 and 2.26. But this was dominated by Pappas et al.′s [32] study due to their large sample size. This study retrospectively analysed clinical data from all patients seen by specialist centres with no specific diagnosis or treatment defined. Additionally, it was considered ‘poor’ reporting quality (File S5). When data from this study of poor reporting quality were excluded, between‐study heterogeneity improved (49.58%), and the prediction interval narrowed to [0.41, 1.35] indicating moderate confidence in the precision of the estimate that people of Black ethnicity are 25.9% less likely to have telangiectasia than their counterparts of White ethnicity (RR = 0.74 [0.63, 0.88], *z* = −3.53, *p* < 0.01) (Figure 2, File S6). Furthermore, another study of ‘good’ reporting quality recognised that there was a higher proportion of patients of Black ethnicity with C0 (no) skin changes before their procedure for vein ablation (17.1% vs. 1.6% in White populations and 7.3% in Hispanic populations) [38], which is not captured in the meta‐analysis.

**Figure 3:**
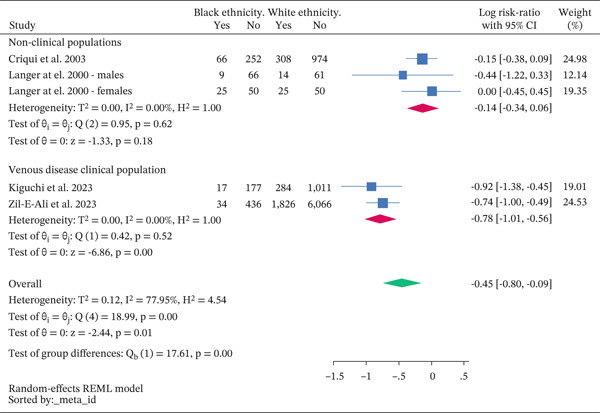
Meta‐analysis of the proportion of people with C2 between dark (Black ethnicity) and light (White ethnicity) skin tones. Note: (1) This was reported as Varicose veins by Criqui et al. [21] and Langer et al. [31]. This was reported as CEAP C2 by Pappas et al. [32], Kiguchi et al. [29] and Zil‐E‐Ali et al. [38] (where the highest classification on CEAP is reported). (2) Data from articles with poor reporting quality [32] have been removed. All data available in Supporting Information S6.

Other findings from known clinical populations found that people who need treatment for venous disease and are Black are 54.2% less likely to have varicose vein classification (C2) on their admission to vascular services than people who need treatment for venous disease who are White (RR = 0.46 [0.36, 0.59], *z* = −6.86, *p* < 0.001) (Figure 3). Insufficient data were available to calculate 95% prediction intervals for C2 skin changes in venous disease populations independently. However, when combined with nonclinical groups and those referred to specialist vascular services, people of Black ethnic backgrounds remained 38.1% less likely to have varicose veins recorded compared to people of White ethnicity (RR = 0.61 [0.46, 0.81], *z* = −3.38, *p* < 0.01) (File S6). However, between‐study heterogeneity was high (*I* ^2^ = 84.66*%*) and prediction intervals were wide (PI = 0.29, 1.53). When studies of poor reporting quality were removed, there remained uncertainty in the data due to a wide prediction interval (PI = 0.18, 2.22) and high heterogeneity (*I* ^2^ = 77.95*%*). However, data indicated that people of Black ethnicity had fewer varicose veins, which remained statistically significant (RR = 0.63 [0.45, 0.91], *z* = −2.44, *p* = 0.01) (Figure 3).

This may be influenced by more people of Black ethnicity having higher classifications on their CEAP score prior to seeing specialists. However, it is also evident that people of Black ethnicity have fewer symptoms of varicose veins than other ethnic groups (3% in African American vs. 5% in White) [32] and have lower severity of varicose veins as captured on VCSS scoring [20, 38]. Nevertheless, a mean VCSS of 0.7 ± 0.9 [20] and 0.72 ± 0.98 [38] in Black populations indicates that some mild signs of varicose are also visible in people of this ethnic group.

This visibility of varicose veins across ethnic groups is inconsistent across the spectrum of dark skin tones, as populations with people of Hispanic ethnicity have more C2 classifications than their counterparts of White ethnicity (Table 1) [20, 29, 32] and people from Southeast Asia with known C4–C6 venous disease also present with significantly more varicose veins than age‐matched controls (Table 1) [26]. This indicated that variation in clinical (C) classification is evident across different ethnic groups, rather than being restricted solely in comparison with White ethnicity.

**Figure 4:**
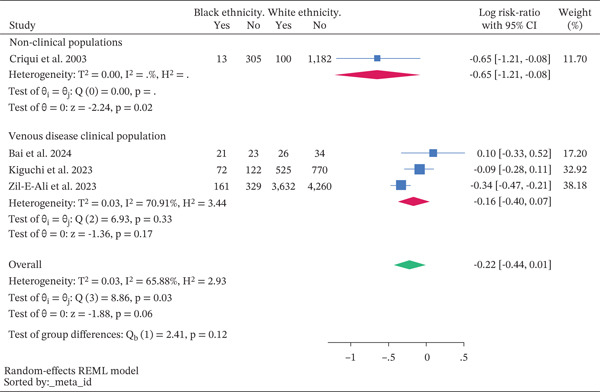
Meta‐analysis of the proportion of people with C3 between dark (Black ethnicity) and light (White ethnicity) skin tones. Note: (1) This was reported as oedema by Criqui et al. [21]. This was reported as CEAP C3 by Bai et al. [19], Kiguchi et al. [29] and Zil‐E‐Ali et al. [38] (where the highest classification on CEAP is reported). (2) Data from articles with poor reporting quality [17, 32] have been removed. All data available in Supporting Information S6.

**Figure 5:**
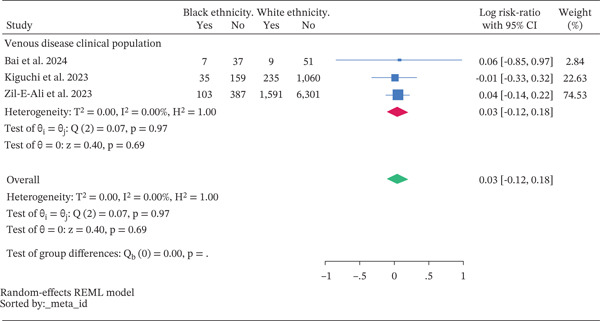
Meta‐analysis of the proportion of people with C4 between dark (Black ethnicity) and light (White ethnicity) skin tones. Note: (1) This was reported as CEAP C4 by Pappas et al. [32], Alsheekh et al. [17], Bai et al. [19], Kiguchi et al. [29] and Zil‐E‐Ali et al. [38] (where the highest classification on CEAP is reported). (2) Data from articles with poor reporting quality [17, 32] have been removed. All data available in Supporting Information S6.

However, as venous disease progresses, the variation between ethnic groups reduces; for example, there is no significant difference between people of White and Black ethnicities on the prevalence of oedema in known venous disease populations (Figure 4) and severity of oedema based on VCSSs [20]. The proportion of people with C4 skin changes is also similar between people of White and Black ethnicities (Table 1 and Figure 5). In some dark‐skinned tone populations, such as Pacific Islanders in Danielsson et al.′s [22] study, these individuals presented with more C4 skin changes than other ethnic groups, indicating that skin changes were clearly visible in this population. This high prevalence of C4 skin changes was attributed by the authors to be related to variation in obesity between ethnic groups, resulting in some ethnic groups having more advanced chronic venous disease [22].

This comparative analysis of clinical presentations is, however, limited by the influence of variations in underlying characteristics and risk factors between ethnic groups, which is acknowledged in several studies. Some studies demonstrated significant differences in patient risk factors across ethnic groups for obesity [20, 22], incidence of DVT′s [20, 24], prevalence of smoking [20], occupation [30], comorbid coronary arterial disease [20], chronic heart failure [24], diabetes [20] and hypertension [20, 24]. Therefore, these limit the accuracy of comparisons across groups without duplex scanning. However, this typically resulted in people of unspecified Asian ethnicity having fewer risk factors than people of White and Black ethnicities, although they tend to have similar or more risk factors than people of White ethnicity.

#### 3.3.3. Reliability

Interrater reliability and intrareliability (test–retest reliability) of assessing skin changes in the lower leg were rarely assessed and poorly reported, with only one study examining interrater reliability of CEAP scores in 25/104 participants. This was 0.97 [33]. However, this was from the whole study population, of which 70.19% were African American. Therefore, it is unknown what proportion of these individuals had a dark skin tone. Additionally, another study demonstrated some inconsistencies in reporting on electronic health records (EHRs) across all skin tones, as presenting signs of spider veins and varicose veins were substantially lower than those classified as C1 and C2 on the CEAP system [32]. Although it is expected that all those classified as C2 would have varicose veins, varicose veins are also present in many individuals with C3â€“C6 skin changes, indicating underreporting.

#### 3.3.4. Clinician Experience

None of the studies in this review reported on healthcare professionals′ experiences of assessing cutaneous manifestations of CVI in people with dark skin tones.

### 3.4. Results on Secondary Outcomes: Impact on Patient Journey and Clinical Care

No eligible studies used a validated tool to measure skin tone, with only one study characterising people based on black, brown and white skin tones [23]. Therefore, the impact on patient journey and clinical care is frequently inferred from ethnic categories due to a lack of standardised assessments to quantify skin tone.

#### 3.4.1. Initial Presentation to Specialist Services

**Figure 6:**
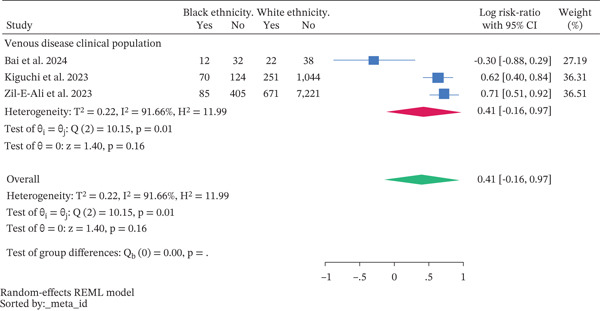
Meta‐analysis of the proportion of people with either active (C6) or healed (C5) venous leg ulcers in dark (Black ethnicity) and light (White ethnicity) skin tones. Note: (1) This was the cumulative value of CEAP C5 and CEAP C6 (where the highest classification on CEAP is reported). (2) Data from articles with poor reporting quality [17, 32] have been removed. All data available in Supporting Information S6.

There are some inconsistencies in the severity of venous disease across skin tones and ethnicities at presentation at specialist centres. However, comparison between White and Black ethnicities indicated that populations of Black ethnicity had a 39% higher chance of presenting to specialists at clinics or receiving surgical interventions for either an active or healed venous leg ulcer than people of White ethnicity (RR = 1.39 [1.01, 1.91], *z* = 2.04, *p* = 0.04) (File S6). However, heterogeneity was high (*I* ^2^ = 89.39*%*), and 95% prediction intervals were very wide (PI = 0.44, 4.45). Furthermore, when poor‐quality studies were removed, the data suggested that there were no differences between people of Black and White ethnicities (Figure 6). But there was a predominance among people with dark skin tones in Brazil seeking help for venous disease [23]. However, some studies suggested that people of White ethnicity were more likely to have healed or active ulcers with inflammation (52.3% vs. 47.9%, *p* < 0.05) compared to people of Black ethnicity [24] and were more likely to have a higher venous severity score [20, 32] compared to their counterparts of Black and Hispanic ethnicities.

Furthermore, one study across 82 centres in the United States identified that the most common symptoms for referral to specialist centres were pain (24%), swelling (14%) and heaviness (12%) in the legs [32]. This indicates that recognition of skin changes across skin tones may have a limited effect on specialist referrals.

#### 3.4.2. Effectiveness of Interventions

There were some notable disparities in treatment outcomes. Four studies evaluated longitudinal outcomes using VCSSs across ethnic groups when the same treatment was provided (Table 1).

However, comparison results between studies were limited by the range of interventions used and different durations of follow‐up. Nevertheless, people of Black ethnicity who underwent ablation alone appeared less likely to have clinical improvements in their VCSSs after intervention [34] and less improvement in the VCSSs overall [32, 38] compared to individuals of White and Hispanic/Latino ethnicity. However, the study by Pappas et al. [32] indicated that undergoing ablation alone for both White and Black ethnicities was less effective than in Hispanic and Asian groups. Furthermore, when adjusting for participant characteristics, there was inconsistency in the findings [34][38], although adjustments differed on the variables they included in this analysis (e.g. insurance status, other past medical history and use of compression therapy), which may have influenced results and could be clinically relevant. For those who underwent Iliac vein stenting, there were no significant differences in VCSSs across ethnic groups until patients reached 3 years of follow‐up [20]. Evaluation of patient‐reported symptoms across ethnic groups was limited, as it was reported only in a small number of studies. Mean improvements in pain scores were significantly lower in people of Black ethnicity compared to other groups at follow‐up on the VCSS scale (0.74 ± 0.99 vs. 1.07 ± 1.0 in White and 1.22 ± 0.94 in Hispanic, *p* < 0.01) [38]. However, this was only considered immediately after the procedure in other studies where ethnic groups had no impact on pain [17, 30]. Other patient‐reported symptoms at follow‐up were only independently reported by ethnicity in the study by Zil‐E‐Ali et al. [38], who concluded that people of Black ethnicity had inferior improvements in their symptoms after their procedures. The author attributed these differences to be associated with socioeconomic differences, healthcare access, lifestyle choices and genetic factors.

#### 3.4.3. Reinterventions and Treatment Costs

People of Black ethnicity have higher treatment costs and significantly more debridements compared to their White counterparts ($7116 vs. $6281, *p* < 0.001, and 17.8% vs. 6.5%, *p* < 0.001, respectively) [24]. People of Black ethnicity also required more reinterventions and were more likely to require a major reintervention compared to other ethnic groups [20].

#### 3.4.4. Impact of Compression Use on Patient Outcomes and Journey

People of Black ethnicity improved their compliance with compression stockings after their intervention, but these improvements were significantly lower than those of people from other ethnic groups (*p* < 0.001) [38]. Additionally, when adding the use of compression in regression models, ethnicity did not have an independently significant effect on clinical improvement [34], indicating that between‐group variation in postoperative behaviours may also have an impact on longitudinal outcomes.

#### 3.4.5. Patient Experience

None of the studies included in this review reported on patient experience.

### 3.5. Certainty of Evidence

Findings indicate low certainty that people with dark skin tones may have venous disease without truncal varicose veins (C2) and telangiectasias (C1), indicating that these may be less readily observed in people with dark skin tones. This was largely attributed to consistent findings across observational studies and well‐reported studies from the independent living population (Table 2), thus indicating that these skin changes may be unreliable in people from dark skin tone groups, specifically people of Black and Thai ethnicities.

**Table 2:** Certainty of evidence.

Outcome	CEAP C1 (telangiectasias) and absence of skin changes—nonclinical populations	CEAP C1 (telangiectasias) and absence of skin changes—clinical populations	Varicose veins (CEAP C2) and the severity of varicose veins—nonclinical populations	Varicose veins (CEAP C2) and the severity of varicose veins—clinical populations	CEAP C3: Oedema	C4 skin changes	People at initial presentation to specialist services or requiring treatment	Severity of venous disease	Treatment outcomes
Finding summary	‐People of Black ethnicity have a lower likelihood of telangiectasias (CEAP C1) compared with people of White ethnicity.	‐People of Black ethnicity undergoing procedures for venous disease may be more likely to have no skin changes (C0) compared with people of White ethnicity.	‐People of Black ethnicity report fewer varicose veins. Findings from other non‐White populations are inconsistent.	‐People of Black and Thai ethnicities are less likely to have visible varicose veins compared with people of White ethnicity. People of Black ethnicity have fewer and less severe varicose veins compared with people of White ethnicity.	‐Oedema (CEAP C3) associated with venous disease appears to present across skin tone and ethnic groups, with no consistent differences between groups.	‐C4 skin changes associated with venous disease appear to be present across skin tones, ethnic and racial groups, with no consistent differences between groups.	‐People of Black ethnicity are more likely to present with venous leg ulcer than people of White ethnicity. However, findings are inconsistent, highly heterogeneous and sensitive to sensitivity analysis.	‐Severity of venous disease is dependent on occupation, lifestyle choices and past medical history.	‐People of Black ethnicity have lower clinical improvement after interventions compared with people of White ethnicity. ‐ However, there was between‐group variation in the type of interventions provided and postprocedure patient behaviour.

Number of studies	2	2	2	8	8	10	7	7	8

Number with dark skin tones by population (number of studies)	Black: 468	Black: 11,816	Black: 468	Black: 12,059	Black: 12,510 (7 studies)	Black: 12,192 (6 studies)	Black: 14,746 (7 studies)	Black: 3369 (5 studies)	Black: 14,621 (7 studies)
	Hispanic: 488	Hispanic: 12,619	Hispanic: 488	Hispanic: 13,190	Hispanic: 13,725 (7 studies)	Hispanic: 13,387 (6 studies)	Hispanic: 13,387 (6 studies)	Hispanic: 1198 (3 studies)	Hispanic: 12,951 (5 studies)
				Asian (SE) 458	Asian (SE): 260 (1 study)	Asian (SE): 380 + 32 limbs (4 studies)		Asian (SE): 119 (2 studies)	Asian (SE): 3 (1 study)
						Pacific Islanders: 37 (limbs) (1 study)		Brown: 63 (1 study)	

Relative effect (from meta‐analysis)	‐Overall estimate of all studies: RR 0.73 [0.61, 0.88], *p* < 0.001 ‐No poor‐quality studies included in this analysis	‐Meta‐analysis not possible as different patient locations (1 study referred to a specialist, 1 study diagnosed with venous disease)	‐Overall estimate of all studies: RR 0.87 [0.71, 1.06] ‐No poor‐quality studies included in this analysis	‐Referral to specialist services: Not pooled (1 study) Overall estimate of those diagnosed with venous disease: RR 0.46 [0.36, 0.57]; *p* < 0.001 ‐No poor‐quality studies included in this analysis	‐Overall estimate of all studies: RR 0.90 [0.76, 1.07], *p* = 0.26 Sensitivity analysis removing poor‐quality studies: RR = 0.80 [0.64–1.01], *p* = 0.06	‐Overall estimate of all studies: RR 1 [0.96, 1.04], *p* = 0.99 ‐ Sensitivity analysis removing poor‐quality studies: RR = 1.03 [0.89, 1.20], *p* = 0.69	‐Overall estimate of all studies: RR 1.39 [1.01, 1.92], *p* < 0.04 ‐ Sensitivity analysis removing poor‐quality studies: 1.51 [0.85, 2.64], *p* = 0.16	Meta‐analysis not possible due to heterogeneity in measurement and outcomes reported	Meta‐analysis not possible due to heterogeneity in intervention type and follow‐up

Trend of other data	‐Results for Hispanic populations were inconsistent (more, less and similar) compared to White populations.	‐A higher proportion of people of Black ethnicity required treatment for venous disease despite an absence of visible C1 skin changes compared to people of White and Hispanic ethnicities.	‐Results for Hispanic populations were inconsistent (more, less and similar) compared to White populations.	‐A large proportion of venous disease identified on duplex ultrasound occurred without visible varicose veins (data only obtained in Thai populations). ‐People of Black ethnicity generally had fewer and less severe visible varicose veins compared to those of White ethnicity. ‐Results for Hispanic populations were inconsistent.	‐Results were inconsistent, with no consistent pattern observed across groups.	‐There was a trend for fewer skin changes in populations with dark skin tones (Black, Hispanic and South Asian) than in White populations. ‐Inconsistency across groups, as Pacific Islanders had the most skin changes.	‐Some inconsistency in which ethnic groups have worse severity of venous disease at presentation to specialists. ‐There was a trend for people with dark skin tones to have a lower composite VCSS (less severe symptoms). ‐Other studies suggested that people of Black ethnicity are less likely to have inflammation of their venous leg ulcers compared to White populations.	‐Results are inconsistent on the relationship between the severity of venous disease and ethnicity. ‐Some suggest that people with dark skin tones may have an earlier onset. ‐Analysis of covariants indicates that between‐group variation is dependent upon occupation, lifestyle choices and past medical history.	‐There were lower clinical improvements when a single surgical intervention was provided to people of Black ethnicity compared to those of White ethnicity receiving the same intervention. ‐However, across‐group variations existed in the treatment provided and compression use after interventions.

Study design overview	All observational studies (starting level: Low)	All observational studies (starting level: Low)	All observational studies (starting level: Low)	All observational studies (starting level: Low)	All observational studies (starting level: Low)	All observational studies (starting level: Low)	All observational studies (starting level: Low)	All observational studies (starting level: Low)	All observational studies (starting level: Low)

Risk of bias	High reporting quality: Outstanding x1 ‐Good x1	Varied reporting quality: Good x1 Poor x1	Varied reporting quality: Outstanding x1 Good x1	Varied reporting quality: Outstanding x1 Excellent x1 Good x4 Poor x2	Varied reporting quality: Excellent x1 Good x5 Poor x2	Varied reporting quality: Outstanding x1 Excellent x1 Good x6 Poor x2	Some concerns in reporting quality: Good x5 Poor x2	Varied reporting quality: Outstanding x1 Good x5 Poor x1	Some concerns in reporting quality: Good x5 Poor x3

Inconsistency	‐Findings were consistent for Black populations, showing fewer telangiectasias. ‐Findings were inconsistent for Hispanic populations.	‐Prevalence estimates were similar across ethnic groups. ‐No formal methods were used to assess agreement or equivalence.	‐Findings were consistent for the Black population, showing fewer varicose veins. ‐Findings were inconsistent for Hispanic populations.	‐Findings were consistent for Black, showing fewer and less severe varicose veins. ‐Duplex‐confirmed venous disease without visible C2 signs was consistently reported where assessed. ‐Findings were inconsistent across other ethnic groups, particularly Hispanic populations.	‐Findings showed variability across studies. ‐Differences between groups were generally nonsignificant. ‐No formal methods were used to assess agreement or equivalence.	‐Findings showed variability across studies. ‐Differences between groups generally were nonsignificant. ‐No formal methods were used to assess agreement or equivalence.	‐Data was inconsistent on the relative proportion of individuals with C5/C6. Most supported the trend and direction of the pooled effect that people of Black ethnicity had more severe venous disease.	‐Covariants impacting the severity of venous disease were not reported consistently across studies.	‐Findings were consistent for studies reporting a single intervention but inconsistent where multiple surgical interventions were combined (i.e. ablation, phlebectomies and UGFS).

Indirectness	‐Outcomes were directly aligned with study objectives. ‐Ethnicity was used as a proxy for skin tone, limiting direct applicability to skin tone–related visibility.	‐Outcomes were indirect as they were not primary outcomes. ‐Ethnicity was used as a proxy for skin tone, limiting direct applicability to skin tone–related visibility.	‐Outcomes were directly aligned with study objectives in nonclinical groups and indirect for patient populations, as outcomes were unrelated to the primary research question. ‐Ethnicity was used as a proxy for skin tone, limiting direct applicability to skin tone–related visibility.	‐Outcomes were indirect as varicose veins were not the primary outcome and were reported as baseline or descriptive data. ‐Ethnicity was used as a proxy for skin tone, limiting direct applicability to skin tone–related visibility.	‐Outcomes were indirect as oedema was not the primary outcome and reported as baseline or descriptive data. ‐Ethnicity was used as a proxy for skin tone, limiting direct applicability to skin tone–related visibility.	‐Outcomes were indirect as C4 skin changes were not the primary outcome and were reported as baseline or descriptive data. ‐Ethnicity was used as a proxy for skin tone, limiting direct applicability to skin tone–related visibility.	‐Outcomes were sometimes directly related to the research question, but they were frequently collected for descriptive purposes rather than to address the primary research question. ‐Ethnicity was used as a proxy for skin tone, limiting direct applicability to skin tone–related visibility.	‐Outcomes were indirect as covariants were not the primary outcome and reported as baseline or descriptive data. ‐Outcomes were not adjusted by key confounders, limiting the independent effect of skin tone or ethnicity on severity. ‐Ethnicity was used as a proxy for skin tone, limiting direct applicability to skin tone–related visibility.	‐Outcomes were directly aligned with study objectives and study endpoints. ‐Ethnicity was used as a proxy for skin tone, limiting direct applicability to skin tone–related visibility.

Imprecision	‐Confidence intervals were narrow, suggesting acceptable precision.	‐One study had wide confidence intervals, reducing precision.	‐The confidence intervals were wide in the nonpatient population but much lower in patient cohorts, suggesting imprecision overall.	‐Small sample sizes were in duplex studies, limiting the precision of estimates. ‐Confidence intervals were moderately narrow, suggesting acceptable precision.	‐Confidence intervals were narrow in patient population groups but wide in nonclinical groups, suggesting imprecision overall.	‐Confidence intervals were fairly narrow but focused around the midpoint.	‐Confidence intervals were fairly wide, particularly after excluding poor‐quality studies.	‐No meta‐analysis was possible for this data. ‐Narrative synthesis suggests consistency in findings.	‐No meta‐analysis was possible for this data. Narrative synthesis suggests consistency in findings.

Publication bias	Unlikely, as outcomes in the included studies	Unclear, as not related to the primary outcome and may not have been consistently reported	Unlikely in nonclinical studies because it was a focus (central outcome)	Unclear, as not related to the primary outcome and may not have been consistently reported	Unclear, as not related to the primary outcome and may not have been consistently reported.	Unclear, as not related to the primary outcome and may not have been consistently reported.	Unlikely, as disease severity at presentation is fundamental in describing populations, and, therefore, was always reported when collected	Unclear, as not related to the primary outcome and may not have been consistently reported.	Unlikely, as treatment outcomes were one of the study endpoints or objectives for studies monitoring and, therefore, were always reported when collected

GRADE confidence	Low certainty	Very low certainty	Low certainty	Low certainty	Overall: Very low	Overall: Very low	Overall: Very low	Overall: Very low	Overall: Low

Justification of GRADE score	‐Observational study design (starts low) ‐Consistent findings for Black ethnicity, directly to outcomes, low publication bias and acceptable precision ‐No large effect present to justify an upgrade	‐Observational study design (starts low) ‐Concerns due to inconsistent indirect outcome reporting, variable reporting quality and unclear publication bias ‐Overall certainty was, therefore, downgraded to very low	‐Observational study design (starts low) ‐Consistent findings in nonclinical populations of Black ethnicity, with results directly aligned with research outcomes and low publication bias. No large effect present to upgrade	‐Observational study design (starts low) ‐Certainty was maintained due to generally consistent findings for populations with dark skin tones, particularly among people of Black ethnicity and during Duplex analysis in Thai populations. ‐Some concerns about this inconsistency and mix of direct/indirect data reported but not enough concerns to downgrade ‐No large effect present to upgrade	‐Observational study design (starts low) ‐Concerns due to inconsistent, indirect outcome reporting, imprecision and unclear reporting bias ‐Overall certainty was, therefore, downgraded to very low	‐Observational study design (starts low) ‐Concerns due to inconsistent, indirect outcome reporting, imprecision and unclear reporting bias ‐Overall certainty was, therefore, downgraded to very low	‐Observational study design (starts low) ‐Concerns due to the inconsistency and sensitivity of findings to study quality ‐Overall certainty was, therefore, downgraded to very low	‐Observational study design (starts low) ‐Concerns due to inconsistent reporting of covariants and bias in the selection of participants ‐Overall certainty was, therefore, downgraded to very low	‐Observational study design (starts low) ‐Grade maintained due to outcomes, direct study endpoints and consistent findings for single interventions ‐No meta‐analysis present to determine a large effect

However, findings indicate that the impact this has on condition severity is unclear due to severity being predominantly influenced by lifestyle choices, health‐seeking behaviours and medical history. Nevertheless, we have very low certainty that people with dark skin tones, specifically those of Black ethnicity, present to specialist services later and low certainty that they have lower clinical improvements after treatment compared to their counterparts with light skin tones (Table 2). This may be attributed to delays in referral and treatment. However, other lifestyle factors, delays in seeking healthcare services and behaviour choices such as compression therapy compliance may also influence patient outcomes.

## 4. Discussion

This systematic review provides a fresh perspective into CVI through the lens of health inequity and offers new insights into how ethnicity influences the assessment, recognition and treatment of venous disease, specifically the disparities affecting people of Black ethnicity. However, it can only infer the influence skin tone has on these outcomes due to a lack of standardised reporting of skin tone. As a result, the diagnostic value of cutaneous manifestations in people with dark skin could not be directly evaluated. Instead, findings relied heavily on racial and ethnic categories as imperfect proxies for skin tone. This, however, highlights the need for routine reporting of skin tone in research and EHR to create the conditions for clinical presentation to be analysed distinctly from the cultural and social constructs of ethnicity.

Despite the lack of skin tone reporting restricting direct evaluation, the evidence from this review indicates some clinically relevant findings. Firstly, the cutaneous manifestations of CVI, notably venous oedema (C3) and skin changes (C4) (e.g. hemosiderin pigmentation and venous eczema), can be identified and stratified according to CEAP classification by appropriately trained clinicians across ethnic groups (Table 1). This is supported by findings elsewhere, where studies in African populations have demonstrated a correlation between CEAP (C) classifications with VCSS in patients with venous disease in Cameroon [40]. Similarly, of those diagnosed with CVI undergoing surgical interventions in Tanzania, 95.9% presented with oedema, and 80.4% presented with hemosiderin pigmentation or eczema [41]; in Côte d′Ivoire, 86% with CVI diagnosed on duplex USS had oedema [42].

Secondly, a central theme from the results highlights that there is variability in the early clinical signs of venous disease across ethics groups, which may have implications for when individuals with dark skin tones should be referred to specialists for duplex scanning and specialist input. NICE guidelines recommend conservative management of asymptomatic varicose veins with no skin changes [43]. However, 22% may develop a venous leg ulcer within 6 years; hence, intervention is recommended for symptomatic truncal varicosities (CEAP C2s and C3s) or with early presentation of skin changes (CEAP C4) [6] [43]. However, evidence from studies in this review reporting the prevalence and severity of varicose veins in people with known venous disease shows a consistent pattern. Individuals of Black and Southeast Asian ethnicities who either had confirmed venous disease on their duplex scan or required treatment for venous disease frequently had this in the absence of visible varicose veins. Additionally, pooled results of the meta‐analysis of included studies demonstrate that people of Black ethnicity had a lower prevalence of varicose veins and C2 skin changes than people of White ethnicity (Figure 3), supporting that early clinical signs may be subtle or nonvisible in some people with dark skin tones. This is reinforced by an analysis of patients with venous insufficiency seen in vascular outpatients′ clinics in Africa, where visible varicose veins were not observed in 60% of patients in Cameroon [44] and 37.5% of individuals in Côte d′Ivoire [42]. Furthermore, 59.6% of those in Uganda with diagnosed CVI obtained a clinical classification of C0 (no visible or palpable signs of venous disease) on CEAP [45], suggesting difficulty in identifying these skin changes. These findings, alongside global prevalence data showing substantially lower pooled prevalence of varicose veins in Africa (5.5%) compared to Europe (21%) [46], highlight the heterogeneity in the visual presentation of the early stages of venous disease across populations. Recent interviews of UK healthcare professionals further highlight that subtle cutaneous manifestations and ambiguous skin changes cause barriers to lower limb assessment in people with dark skin tones, necessitating a detailed patient and symptom history to obtain an accurate diagnosis [47]. Therefore, based on this collective evidence, it is suggested that relying on visible varicose veins may disadvantage people with dark skin tones, delaying diagnosis. Therefore, this review hypothesises that the development of venous leg ulcers could be reduced for people with dark skin tones if referrals to specialist vascular teams were based on symptoms consistent with symptomatic varicose veins, such as pain, itching, throbbing and aching, in the absence of visible varicose veins. This suggestion may benefit people with dark skin tones, improving equity in access to timely vascular assessment and patient outcomes [48]. Thus, it may help bring parity to treatment access and ensure equitable care to all individuals, regardless of the complexity of visually identifying varicose veins during physical examination.

However, this proposal may challenge some locally integrated care boards (former clinical commissioning groups) as only 21.5% are fully compliant with existing referral criteria for varicose vein treatment [48], which do not include lower limb pain, swelling and itching when varicose veins cannot be visualised within the funding treatment on the NHS [49]. Due to the increased burden additional referrals and duplex ultrasound scanning will have on healthcare resources, further investigation is required to test this hypothesis, specifically into the specificity and sensitivity of CVI symptoms in the absence of visible varicose veins in people with dark skin tones, to determine CVI pathophysiology. If this is implemented, these changes will challenge existing referral and commissioning criteria that rely heavily on visible varicose veins. However, they also have the potential to establish a symptom‐based pathway that ensures equitable access to specialist vascular assessment.

This review also highlights important implications for patient pathways and outcomes. The findings demonstrate that the prevalence of venous leg ulcers was higher in people of Black ethnicity, although this finding was sensitive to study quality. This disparity may reflect the previously described subtle skin changes combined with the limited use of symptom‐based referral pathways. However, ineffective referral pathways and a lack of established pathways to specialist services transcend all ethnicities and skin tones, often as a result of inconsistent application of referral guidance and a reliance on private healthcare, offsetting these gaps for people with sufficient financial resources [50]. Differences between groups may also contribute to variations in disease severity across ethnic groups. For example, risk factors and health‐related behaviours, which are associated with venous severity, differed across ethnicities [20, 24]. Additionally, as seen in other lower limb ulcers, ethnicity may be associated with barriers to accessing healthcare, including difficulty attending and travelling to clinics, financial costs and language or communication barriers [51], thereby highlighting a complex interplay that this data on ethnicity may have on delaying access to specialist services and treatments.

The collective evidence from studies in this systematic review also showed disparities in long‐term outcomes and reintervention rates for people with dark skin tones, specifically those of Black ethnicity. These individuals have higher treatment costs [24] and require more reinterventions [20]. These disparities may be associated with the severity of venous disease at presentation at vascular services; however, poorer outcomes are also likely to be influenced by a combination of socioeconomic factors, healthcare access, health‐seeking behaviours and the prevalence of comorbid conditions such as obesity. Therefore, the correlation between skin tones and health outcomes in people with CVI warrants further investigation. These cross‐ethnic variations in comorbidities and lifestyle have been reported in several studies, specifically occupation [30], obesity [20, 22] and the number of comorbidities [20, 24], which are all likely to have an impact on health outcomes. This may be further exacerbated by ethnic variations in access to healthcare and health‐seeking behaviours, which have been reported in previous studies [52]. Additionally, health behaviours after interventions may vary across groups, such as concordance with compression therapy [38]. This may be clinically relevant as pooled results from a recent systematic review demonstrate that compression improves pain and supports return to normal activities [53], and current clinical guidance recommends that postprocedural compression should be considered and is widely advised by the majority of surgeons [6]. This highlights the importance of collecting both data on compression concordance in research studies (e.g. on the compression usage component of the VCSS scale) and whether compression was advised by the surgeon or not. Such information is essential to determine where differences exist and whether future interventions target clinician practice, patient behaviours or both.

In summary, this review establishes actionable insights and highlights important equity issues in CVI influenced not only by biological factors but also by diagnostic visibility, socioeconomic barriers and health‐seeking behaviours. This system‐level perspective advances current understanding and highlights the need for equity‐focused clinical pathways and the importance of enhanced clinical training on recognising CVI across skin tones and in undertaking a holistic lower limb assessment, particularly where cutaneous manifestations of CVI may be subtle in people with dark skin tones.

Throughout this review, findings should be treated with caution due to skin tone being poorly reported and ethnicity being used as a surrogate. This has been noticeable within the undefined Asian population by Danielsson et al. [22], who reported that their unspecified Asian population consisted of predominantly Japanese and Chinese participants, highlighting that a generic Asian population cannot separate light and dark skin tones. This variation across Asian populations is also noted by Murli et al. [30], where there was a variation in occupations of people across ethnic groups, which would have an impact on standing long hours, a risk factor for CVI. Furthermore other studies considering a generic Asian population found fewer comorbidities and healthier BMIs than their counterparts with light skin tones [20], whereas South Asian groups in the United Kingdom experience higher levels of cardiovascular disease and similar levels of obesity to White British people, and East Asian (Chinese) populations have significantly less obesity [52], further highlighting that the analysis of a generic Asian population may not be indicative of skin tone. These variations across ethnic groups prevented the Asian subgroup from being used in the analysis due to not knowing which skin tones this represented. However, this may be equally important across all non‐White ethnicities, as people in America, where the majority of studies originate, use self‐identification to reflect an individual′s racial and ethnic identities. This is often constructed from family ancestry and social affiliations, which may not be directly associated with skin colour [54]. This is exemplified in a large American study where people identifying as ethnically Black were often described by interviewers as medium brown skin (30%), dark brown skin (30%), black skin (30%) or light brown skin (11%) [54]. The review, therefore, identifies a need for this documentation and sets out a clear agenda for future research to include routine documentation of skin tone, reported using a validated tool such as the skin tone chart [55], in both research and in EHRs, independently of ethnicity, to improve diagnostic equity and better understand the contribution of skin tone independently of the social constructs of ethnicity. Furthermore, clearly documenting skin tone in all studies will enable findings from countries with predominantly dark skin tones to be better utilised internationally.

### 4.1. Limitations

With the absence of studies reporting skin tone, reviewers used ethnicity as a surrogate. It is, therefore, unclear how much of the results obtained are due to skin tone or whether ethnic variations influenced healthcare. This search terminology, however, enabled articles to be obtained for the review which would not have been possible without using this surrogate population. Additionally, studies in which ethnic groups or skin tones were not used to describe populations or were not indexed in the research could not be obtained through the database searching. This was apparent in studies from sub‐Saharan Africa (used in the discussion), which do not report information on skin tone, ethnic or racial groups. However, they are likely to include a large proportion of individuals with dark skin tones, further highlighting the importance of reporting skin tone across all studies, so learning can be applied in other contexts.

In the absence of triangulation of vascular interventions with underlying pathophysiology, duplex scans and clear CEAP classification, it was assumed that interventions for venous disease were undertaken when clinically warranted based on best available evidence. However, it is unclear whether this occurred and if other factors may have influenced the treatment provided.

Furthermore, several articles that have supported findings within this review are only preliminary reports and, therefore, have not undergone a peer review or were not available in English and required translation. These were, therefore, not obtained through database searching and indexing but have been included in the discussion. This may highlight challenges and bias to publishing for some, but it may also raise questions regarding the quality assurance of these articles, as these were not subjected to the scrutiny of peer review by world‐leading experts.

## 5. Conclusion

This systematic review provides the first comprehensive synthesis of evidence on the impact skin tone has on CVI. Findings suggest that reliance on visible varicose veins and cutaneous manifestations of CVI may disadvantage people with dark skin tones by contributing to delayed diagnosis and subsequently could influence clinical outcomes. Current guidelines and commissioning criteria do not adequately address that varicose veins may be visible, subtle or nonvisible in people with dark skin tones, which may exacerbate health inequity. Based on these findings, the authors propose that clinicians adopt a comprehensive, holistic lower leg assessment and prompt referral to specialist vascular services for duplex ultrasound of symptomatic varicose veins, even in the absence of visible varicose veins in people with dark skin tones. This review, therefore, advances the understanding of CVI as an equity issue and supports the development of more inclusive diagnostic and referral guidance, which may have a positive impact on patient care and equitable longitudinal outcomes.

However, future research should consider the influence that wider determinants of health and healthcare behaviours have on patient outcomes and the specificity and sensitivity of CVI symptoms in the absence of visible varicose veins in determining CVI pathophysiology.

A lack of documentation of skin tone in research and within EHRs and published research has resulted in ethnicity being used as a proxy in reported studies to determine skin tone. The authors recognise this as a limitation to the review and advocate for the reporting of skin tone in clinical research and EHR. This has the potential to clarify the influence of the variation in clinical presentation across skin tones independently of the sociocultural and behavioural influences of ethnicity. Addressing these gaps in the reporting of data is critical to understanding equity in healthcare, where cutaneous manifestations differ across skin tones.

## Author Contributions

Victoria J Clemett: conceptualisation, methodology, investigation, validation, formal analysis, data curation, writing (original draft), visualisation and project administration. Jemell Geraghty: conceptualisation, methodology, investigation and writing—review and editing. Neesha Oozageer Gunowa: conceptualisation, methodology, investigation and writing—review and editing. Sue Woodward: conceptualisation, methodology, investigation, formal analysis, writing (review and editing) and supervision. Tesfamariam Aklilu Betemariam: investigation, validation and writing—review and editing. Audrey Edomobi: investigation, validation and writing—review and editing. Alice Milsom: investigation, validation and writing—review and editing. Toby Prevost: statistical advice. Riana John: resources and investigation. Lukla Biasi: conceptualisation, writing (review and editing) and supervision.

## Funding

This work was supported by department funding at King′s College London for research assistant time. No other specific funding was received, but the review was performed as part of the employment of the authors at either King′s College London or Guy′s and St Thomas′ NHS Foundation Trust.

## Conflicts of Interest

One author is a Trustee of the Leg Club Foundation and a coalition member of the Legs Matter Campaign. No other conflicts of interest are declared.

## Supporting Information

Additional supporting information can be found online in the Supporting Information section.

## Supporting information


**Supporting Information 1** File S1: PRISMA checklist.

**Supporting Information 2** File S2: Clinical etiological anatomical pathophysiological (CEAP) classification.

**Supporting Information 3** File S3: Venous clinical severity score (VCSS).

**Supporting Information 4** File S4: Search strategy (4a) and search overview (4b) by database.

**Supporting Information 5** File S5: Risk of bias assessment for included studies by study type: (5a) cross‐sectional studies, (5b) cohort studies, (5c) case series and (5d) case report.

**Supporting Information 6** File S6: Sensitivity analysis showing both inclusion and exclusion of studies rated poor reporting quality.

## Data Availability

The authors confirm that the data supporting the findings of this study are either available within the article and its Supporting Information or at King′s College London research data repository, KORDS. Data and calculations for meta‐analysis are openly available at the King′s College London research data repository, KORDS (10.18742/30271972).

## References

[1] Eberhardt R. T. and Raffetto J. D. , Chronic Venous Insufficiency, Circulation. (2014) 130, no. 4, 333–346, 10.1161/CIRCULATIONAHA.113.006898. 25047584

[2] Atkin L. , Bućko Z. , Montero E. C. , Cutting K. , Moffatt C. , Probst A. , Romanelli M. , Schultz G. S. , and Tettelbach W. , Implementing TIMERS: The Race Against Hard-to-Heal Wounds, Journal of Wound Care. (2019) 23, no. Supplement 3a, S1–S50, 10.12968/jowc.2019.28.Sup3a.S1. 30835604

[3] Guest J. F. , Fuller G. W. , and Vowden P. , Cohort Study Evaluating the Burden of Wounds to the UK′s National Health Service in 2017/2018: Update From 2012/2013, BMJ Open. (2020) 10, no. 12, e045253, 10.1136/bmjopen-2020-045253. PMC7757484 33371051

[4] Herber O. R. , Schnepp W. , and Rieger M. A. , A Systematic Review On The Impact Of Leg Ulceration On Patients′ Quality Of Life, Health and Quality of Life Outcomes. (2007) 5, 10.1186/1477-7525-5-44. PMC1947954 17651490

[5] Phillips P. , Lumley E. , Duncan R. , Aber A. , Woods H. B. , Jones G. L. , and Michaels J. , A Systematic Review of Qualitative Research Into People′s Experiences of Living With Venous Leg Ulcers, Journal of Advanced Nursing. (2018) 74, no. 3, 550–563, 10.1111/jan.13465. 28960514

[6] De Maeseneer M. G. , Kakkos S. K. , Aherne T. , Baekgaard N. , Black S. , Blomgren L. , Giannoukas A. , Gohel M. , de Graaf R. , Hamel-Desnos C. , Jawien A. , Jaworucka-Kaczorowska A. , Lattimer C. R. , Mosti G. , Noppeney T. , van Rijn M. J. , Stansby G. , Esvs Guidelines Committee , Kolh P. , Bastos Goncalves F. , Chakfé N. , Coscas R. , de Borst G. J. , Dias N. V. , Hinchliffe R. J. , Koncar I. B. , Lindholt J. S. , Trimarchi S. , Tulamo R. , Twine C. P. , Vermassen F. , Wanhainen A. , Document Reviewers , Björck M. , Labropoulos N. , Lurie F. , Mansilha A. , Nyamekye I. K. , Ramirez Ortega M. , Ulloa J. H. , Urbanek T. , van Rij A. M. , and Vuylsteke M. E. , Editor′s Choice - European Society for Vascular Surgery (ESVS) 2022 Clinical Practice Guidelines on the Management of Chronic Venous Disease of the Lower Limbs, European Journal of Vascular and Endovascular Surgery. (2022) 63, no. 2, 184–267, 10.1016/j.ejvs.2021.12.024. 35027279

[7] Gohel M. S. , Heatley F. , Liu X. , Bradbury A. , Bulbulia R. , Cullum N. , Epstein D. M. , Nyamekye I. , Poskitt K. R. , Renton S. , Warwick J. , and Davies A. H. , A Randomized Trial of Early Endovenous Ablation in Venous Ulceration, New England Journal of Medicine. (2018) 378, no. 22, 2105–2114, 10.1056/NEJMoa1801214. 29688123

[8] Gohel M. S. , Mora J. , Szigeti M. , Epstein D. M. , Heatley F. , Bradbury A. , Bulbulia R. , Cullum N. , Nyamekye I. , Poskitt K. R. , Renton S. , Warwick J. , and Davies A. H. , Long-Term Clinical and Cost-Effectiveness of Early Endovenous Ablation in Venous Ulceration: A Randomized Clinical Trial, JAMA Surgery. (2020) 155, no. 12, 1113–1121, 10.1001/jamasurg.2020.3845. 32965493 PMC7512122

[9] Wounds UK , Best Practice Statement: Holistic Management of Venous Leg Ulceration, 2022, 2nd edition, Wounds UK, https://wounds-uk.com/resources/details/holistic-management-venous-leg-ulceration-second-edition.

[10] Lurie F. , Passman M. , Meisner M. , Dalsing M. , Masuda E. , Welch H. , Bush R. L. , Blebea J. , Carpentier P. H. , De Maeseneer M. , Gasparis A. , Labropoulos N. , Marston W. A. , Rafetto J. , Santiago F. , Shortell C. , Uhl J. F. , Urbanek T. , van Rij A. , Eklof B. , Gloviczki P. , Kistner R. , Lawrence P. , Moneta G. , Padberg F. , Perrin M. , and Wakefield T. , The 2020 Update of the CEAP Classification System and Reporting Standards, Journal of Vascular Surgery. Venous and Lymphatic Disorders. (2020) 8, no. 3, 342–352, 10.1016/j.jvsv.2019.12.075. 32113854

[11] Dhoonmoon L. , Nair H. K. R. , Abbas Z. , Andrews E. , McConnie S. , Pearson J. , Waheed M. , and Wijeyaratne M. , International Consensus Document: Wound Care and Skin Tone Signs, Symptoms and Terminology for All Skin Tones, 2023, Wounds International.

[12] Wounds UK , Best Practice Statement: Addressing Skin Tone Bias in Wound Care: Assessing Signs and Symptoms in People With Dark Skin Tones, 2021, Wounds UK, https://wounds-uk.com/resources/details/addressing-skin-tone-bias-wound-care-assessing-signs-and-symptoms-people-dark-skin-tones.

[13] Gethin G. , Vellinga A. , Tawfick W. , O′Loughlin A. , Mcintosh C. , Mac Gilchrist C. , Murphy L. , Ejiugwo M. , O′Regan M. , Cameron A. , and Ivory J. D. , The Profile of Patients With Venous Leg Ulcers: A Systematic Review and Global Perspective, Journal of Tissue Viability. (2021) 30, no. 1, 78–88, 10.1016/j.jtv.2020.08.003. 32839066

[14] Clemett V. , Oozageer Gunowa N. , Geraghty J. , and Woodward S. , What Influences the Inclusion of Skin Tone Diversity When Teaching Skin Assessment? Findings From a Survey, British Journal of Nursing. (2024) 33, no. 4, 176–186, 10.12968/bjon.2024.33.4.176. 38386525

[15] Dhoonmoon L. , Skin Tone in Lower-Limb Assessment: Results of a UK Survey, Wounds. (2025) 21, no. 1, 37–41, https://wounds-uk.com/journal-articles/.

[16] Vasquez M. A. , Rabe E. , RB M. L. , Shortell C. K. , Marston W. A. , Gillespie D. , Meissner M. H. , Rutherford R. B. , and American Venous Forum Ad Hoc Outcomes Working Group , Revision Of The Venous Clinical Severity Score: Venous Outcomes Consensus Statement: Special Communication Of The American Venous Forum Ad Hoc Outcomes Working Group, Journal of Vascular Surgery. (2010) 52, no. 5, 1387–1396, 10.1016/j.jvs.2010.06.161. 20875713

[17] Alsheekh A. , Hingorani A. , Ferm S. , Kibrik P. , Aurshina A. , Marks N. , and Ascher E. , Is There an Effect of Race/Ethnicity on Early Complications of Iliac Vein Stenting?, Vascular. (2017) 25, no. 5, 549–552, 10.1177/1708538117699335, 28330434. 28330434

[18] Balasubramanyam S. , Migden M. R. , and Silapunt S. , Venous Treatment of Lipodermatosclerosis to Improve Ambulatory Function, Dermatologic Surgery. (2018) 44, no. 5, 749–752, 10.1097/DSS.0000000000001314. 28858931

[19] Bai H. , Storch J. B. , Gokani V. , Kibrik P. , Chen J. , and Ting W. , Identifying Venous Clinical Severity Score Thresholds for Clinical-Etiology-Anatomy-Pathophysiology Classifications of Venous Edema and Higher, Vascular. (2024) 32, no. 6, 1322–1329, 10.1177/17085381231193510. 37541989

[20] Cho L. D. , Bai H. , Collins L. C. , Chen J. , Cooke P. V. , Kang Y. , Vasan V. , Kim J. , Gonzalez C. , Dionne E. , Kim S. Y. , and Ting W. , Race Differences in Iliofemoral Vein Stenting for Chronic Venous Insufficiency, Vascular. (2022) 32, no. 2, 17085381221140612, 10.1177/17085381221140612. 36395482

[21] Criqui M. H. , Jamosmos M. , Fronek A. , Denenberg J. O. , Langer R. D. , Bergan J. , and Golomb B. A. , Chronic Venous Disease in an Ethnically Diverse Population: The San Diego Population Study, American Journal of Epidemiology. (2003) 158, no. 5, 448–456, 10.1093/aje/kwg166. 12936900 PMC4285442

[22] Danielsson G. , Eklof B. , Grandinetti A. , and Kistner R. L. , The Influence of Obesity on Chronic Venous Disease, Vascular and Endovascular Surgery. (2002) 36, no. 4, 271–276, 10.1177/153857440203600404. 15599477

[23] de Souza E. M. , Yoshida W. B. , de Melo V. A. , Aragão J. A. , and de Oliveira L. A. , Ulcer Due To Chronic Venous Disease: A Sociodemographic Study In Northeastern Brazil, Annals of Vascular Surgery. (2013) 27, no. 5, 571–576, 10.1016/j.avsg.2012.07.021. 23540674

[24] Dua A. , Desai S. S. , and Heller J. A. , The Impact of Race on Advanced Chronic Venous Insufficiency, Annals of Vascular Surgery. (2016) 34, 152–156, 10.1016/j.avsg.2016.01.020. 27179983

[25] Fronek A. , Denenberg J. O. , Criqui M. H. , and Langer R. D. , Quantified Duplex Augmentation in Healthy Subjects and Patients With Venous Disease: San Diego Population Study, Journal of Vascular Surgery. (2003) 37, no. 5, 1054–1058, 10.1067/mva.2003.173. 12756354

[26] Kanchanabat B. , Wongmahisorn Y. , Stapanavatr W. , Kanchanasuttirak P. , and Manomaiphiboon A. , Clinical Presentation and Patterns of Venous Reflux in Thai Patients With Chronic Venous Insufficiency (CVI), European Journal of Vascular and Endovascular Surgery. (2010) 40, no. 3, 399–402, 10.1016/j.ejvs.2010.04.017. 20561800

[27] Kanchanabat B. , Ruangsetakit C. , and Stapanavatr W. , Clinical Characteristics of Thai Chronic Venous Insufficiency (CVI) Patients, Journal of the Medical Association of Thailand. (2017) 100, no. 1, 17–23. 29911374

[28] Kanchanabat B. and Stapanavatr W. , Venous Ultrasonography Findings and Clinical Correlations in 104 Thai Patients With Chronic Venous Insufficiency of the Legs, Singapore Medical Journal/Singapore Medical Journal. (2018) 59, no. 3, 155–158, 10.11622/smedj.2017043. 28503700 PMC5861339

[29] Kiguchi M. M. , Fallentine J. , Oh J. H. , Cutler B. , Yan Y. , Patel H. R. , Shao M. Y. , Agrawal N. , Carmona E. , Hager E. S. , Ali A. , Kochubey M. , and O′Banion L. A. , Race, Sex, and Socioeconomic Disparities Affect the Clinical Stage of Patients Presenting for Treatment of Superficial Venous Disease, Journal of Vascular Surgery. Venous and Lymphatic Disorders. (2023) 11, no. 5, 897–903, 10.1016/j.jvsv.2023.06.001. 37343787 PMC13368408

[30] Murli N. L. , Lee T. C. , and Beh M. L. , Holistic Management of Venous Ulcers Especially With Endovenous Laser Treatment Using 980nm Laser in an Ethnically Diverse Society, Medical Journal of Malaysia. (2013) 68, no. 6, 453–458, https://ncbi.nlm.nih.gov/pubmed/24632912. 24632912

[31] Langer R. D. , Criqui M. H. , Denenberg J. , and Fronek A. , The Prevalence of Venous Disease by Gender and Ethnicity in a Balanced Sample of Four Ethnic Groups in Southern California, Phlebology: The Journal of Venous Disease. (2000) 15, no. 3-4, 99–105, 10.1177/026835550001500303.

[32] Pappas P. J. , Pappas S. F. , Nguyen K. Q. , and Lakhanpal S. , Racial Disparities in the Outcomes of Superficial Vein Treatments for Chronic Venous Insufficiency, Journal of Vascular Surgery. Venous and Lymphatic Disorders. (2020) 8, no. 5, 789–798.e3, 10.1016/j.jvsv.2019.12.076. 32205126

[33] Paul J. C. , Pieper B. , and Templin T. N. , Itch: Association With Chronic Venous Disease, Pain, and Quality of Life, Journal of Wound, Ostomy, and Continence Nursing. (2011) 38, no. 1, 46–54, 10.1097/WON.0b013e318202c47a. PMC3086353 21287771

[34] Pinto Rodríguez P. , Fassler M. , Obi A. , Osborne N. H. , Robinson S. T. , Jacobs B. N. , Aziz F. , Nguyen K. P. , Gwozdz A. M. , Rodriguez L. E. , Fukaya E. , Sachdev U. , Iyad Ochoa Chaar C. , and Research Committee of the American Venous Forum , Factors Associated With Lack of Clinical Improvement After Vein Ablation in the Vascular Quality Initiative, Journal of Vascular Surgery. Venous and Lymphatic Disorders. (2024) 12, no. 4, 101884, 10.1016/j.jvsv.2024.101884. 38552954 PMC11523342

[35] Taengsakul N. , Association of Duplex Ultrasonography Findings With the Severity of Chronic Venous Disease in Thai Patients, Asian Journal of Surgery. (2023) 46, no. 6, 2304–2309, 10.1016/j.asjsur.2022.09.161. 36283874

[36] Taofan T. , Dakota I. , Kartamihardja A. H. A. , Afandy J. E. , Indriani S. , and Adiarto S. , Satisfactory Result of Great Saphenous Vein Endovenous Laser Ablation Until Below the Knee on Active Venous Leg Ulcer: A Case Series, F1000 Research. (2023) 12, 10.12688/f1000research.131695.4. PMC11377932 39246584

[37] Verma S. K. , Kushwaha J. , and Srivastav S. , Morphometric Study of Superficial Veins of Leg in the Chronic Venous Disorders Patients on Duplex Ultrasonography and Its Correlation With Anthropometry and Venous Clinical Severity Score of Patients in the Indian Population: Multicentre Cross Sectional Study, European Journal of Cardiovascular Medicine. (2023) 13, no. 10, 369–377, 10.5083/ejcm/2023.

[38] Zil-E-Ali A. , Dehaven C. , Alamarie B. , Paracha A. W. , and Aziz F. , Black or African American Patients Undergo Great Saphenous Vein Ablation Procedures for Advanced Venous Disease and Have the Least Improvement in Their Symptoms After These Procedures, Journal of Vascular Surgery. Venous and Lymphatic Disorders. (2023) 11, no. 5, 904–912.e1, 10.1016/j.jvsv.2023.06.002. 37343786

[39] Guyatt G. H. , Oxman A. D. , Vist G. E. , Kunz R. , Falck-Ytter Y. , Alonso-Coello P. , Schünemann H. J. , and GRADE Working Group , GRADE: An Emerging Consensus on Rating Quality of Evidence and Strength of Recommendations, British Medical Journal. (2008) 336, no. 7650, 924–926, 10.1136/bmj.39489.470347.AD. 18436948 PMC2335261

[40] Ngatchou W. , Barche B. , Temgoua M. , Metouguena S. E. , Jutcha I. , Mvondo C. M. , Kamdem F. , Dzudie A. , Ndjoh S. , Johne M. , Metogo J. , Ndom M. S. , Sango J. , Ngo Yon C. , Moulium S. , Lade V. , Kuaté L. M. , Menanga A. P. , Sobngwi E. , Njock R. , Blazquez S. B. , and Ngowe Ngowe M. , Prevalence, Clinical Presentation, and Risk Factors of Chronic Venous Disease in Cameroon: A General Population-Based Study, Phlebology. (2024) 39, no. 4, 259–266, 10.1177/02683555231224111. 38158837

[41] Paul C. H. , Shedura V. J. , Nyawawa E. , Mwanga A. , and Byomuganyizi M. , Surgical Treatment in Patients With Chronic Venous Insufficiency Related to Primary Varicose Veins: Findings From Jakaya Kikwete Cardiac Institute, Tanzania, 2025, preprint, reseach square, 09 Feb, 2025 10.21203/rs.3.rs-5976111/v1.

[42] Konin C. , Soya E. , Essoubo M. , Monney E. , N′Loo A. S. E. , Anzouan-Kacou J. B. , and Adoh M. , Chronic Venous Disease in Sub-Saharan African Population: An Overview of Clinical and Epidemiological Aspects, Determining Factors, Angéiologie. (2016) 67, no. 4, https://proquest.com/docview/1901683711.

[43] National Institute for Health and Care Excellence , Varicose Veins Diagnosis and Management. Clinical Guideline [CG168], 2013, https://nice.org.uk/guidance/cg168. 39960993

[44] Fokou M. , Moifo B. , Fongang E. , Teyang A. , and Muna W. , Characteristics of Patients and Patterns of Chronic Venous Disease of the Lower Limbs in a Referral Hospital in Cameroon, Journal of Vascular Surgery. Venous and Lymphatic Disorders. (2018) 6, no. 1, 90–95, 10.1016/j.jvsv.2017.08.012. 29097175

[45] Komurembe E. , Kabweru W. , Majeme P. , Odongo R. , and Magala J. P. , Clinical Presentation and Associated Factors of Lower Limb Chronic Venous Insufficiency at a Tertiary Hospital in Uganda: A Cross-Sectional Study, 2022, preprint, research square August 8th, 2022 10.21203/rs.3.rs-1935797/v1.

[46] Salim S. , Machin M. , Patterson B. O. , Onida S. , and Davies A. H. , Global Epidemiology of Chronic Venous Disease: A Systematic Review With Pooled Prevalence Analysis, Annals of Surgery. (2021) 274, no. 6, 971–976, 10.1097/SLA.0000000000004631. 33214466

[47] Clemett V. , Oozageer Gunowa N. , Geraghty J. , Shahid M. , John R. , Biasi L. , and Woodward S. , The Barriers and Facilitators Healthcare Professionals′ Experience When Assessing the Cutaneous Manifestations of Chronic Venous Insufficiency and Peripheral Arterial Disease in People With Dark Skin Tones: A Qualitative Descriptive Study, International Wound Journal. (2026) 23, no. 3, e70805, 10.1111/iwj.70805. 41771584 PMC12952981

[48] Hitchman L. H. , Mohamed A. , Smith G. E. , Pymer S. , Chetter I. C. , Forsyth J. , and Carradice D. , Provision of NICE-Recommended Varicose Vein Treatment in the NHS, British Journal of Surgery. (2023) 110, no. 2, 225–232, 10.1093/bjs/znac392. 36448204 PMC10364503

[49] Royal College of Surgeons of England , Varicose Veins Commissioning Guide, 2013, Royal College of Surgeons of England, https://www.rcseng.ac.uk/-/media/Files/RCS/Library-and-publications/Non-journal-publications/Varicose-Veins--Commissioning-Guide.pdf.

[50] Pagnamenta F. , Lhussier M. , and Rapley T. , The (Dis)organization of Leg Ulcer Care: A Realist Synthesis, Journal of Advanced Nursing. (2024) 80, no. 12, 4777–4804, 10.1111/jan.16210. 38747461

[51] Highton P. , Jeffers S. , Butt A. , O′Mahoney L. , Jenkins S. , Abdala R. , Haddon L. , Gillies C. , Curtis F. , Hadjiconstantinou M. , and Khunti K. , Patient-Reported Outcomes in Diabetes-Related Foot Conditions: Is Patient Experience Influenced by Ethnicity? A Mixed-Methods Systematic Review, Diabetic Medicine. (2024) 41, no. 10, e15420, 10.1111/dme.15420. 39102339

[52] Raleigh V. , The Health of People From Ethnic Minority Groups in England, 2023, https:/kingsfund.org.uk/insight-and-analysis/long-reads/health-people-ethnic-minority-groups-england.

[53] Ma F. , Xu H. , Zhang J. , Premaratne S. , Gao H. , Guo X. , and Yang T. , Compression Therapy Following Endovenous Thermal Ablation of Varicose Veins: A Systematic Review and Meta-Analysis, Annals of Vascular Surgery. (2022) 80, 302–312, 10.1016/j.avsg.2021.09.035. 34774690

[54] Perreira K. M. , Wassink J. , and Harris K. M. , Beyond Race/Ethnicity: Skin Color, Gender, and the Health of Young Adults in the United States, Population Research and Policy Review. (2019) 38, no. 2, 271–299, 10.1007/s11113-018-9503-3. 31595099 PMC6781627

[55] Ho B. K. and Robinson J. K. , Color Bar Tool for Skin Type Self-Identification: A Cross-Sectional Study, Journal of the American Academy of Dermatology. (2015) 73, no. 2, 312–313.e1, 10.1016/j.jaad.2015.05.024. 26183973 PMC4506490

