# Supplementary material for: A Systematic Review of the Effectiveness of Assessing Skin Changes for Chronic Venous Insufficiency in People With Dark Skin Tones and the Impact on the Patient Journey and Clinical Care
Source: Int J Vasc Med. 2026 Jun 24;2026:8034303. doi: 10.1155/ijvm/8034303 (PMC13291890; doi:10.1155/ijvm/8034303)
Supplement: Supplementary file 2 — Supporting Information 2 File S2: Clinical etiological anatomical pathophysiological (CEAP) classification. [file IJVM-2026-8034303-s002.docx]

# Supplementary file 2: CEAP (Clinical Etiological Anatomical Pathophysiological) Classification (Source: Lurie et al., 2020)

| Clinical (C) class | | |
| --- | --- | --- |
|  | C0 | No visible or palpable signs of venous disease |
|  | C1 | Telangiectasia or reticular veins |
|  | C2 | Varicose veins |
|  | C2r | Recurrent varicose veins |
|  | C3 | Oedema |
|  | C4 | Changes in skin and subcutaneous tissue secondary to CVD |
|  | C4a | Pigmentation of eczema |
|  | C4b | Lipodermatosclerosis or atrophie blanche |
|  | C4c | Corona phlebectatica |
|  | C5 | Healed ulcer |
|  | C6 | Active venous ulcer |
|  | C6r | Recurrent venous ulcer |
|  |  | Symptomatic (S): Including ache, pain, tightness, skin irritation, heaviness, muscle cramps and other complaints attributed to venous disease |
|  |  | Asymotomatic (A) |
| Etiological (E) class | | |
|  | Ep | Primary |
|  | Es | Secondary |
|  | Esi | Secondary – intravenous |
|  | Ese | Secondary – extravenous |
|  | Ec | Congenital |
|  | En | None identified |
| Anatomical (A) class | | |
|  | As | Superficial |
|  | Ad | Deep |
|  | Ap | Perforators |
|  | An | No identifiable venous location |
| Pathophysiological (P) class | | |
|  | Pr | Reflux |
|  | Po | Obstruction |
|  | Pr,o | Reflux and obstruction |
|  | Pn | No pathophysiology identified |
