# Supplementary material for: A Systematic Review of the Effectiveness of Assessing Skin Changes for Chronic Venous Insufficiency in People With Dark Skin Tones and the Impact on the Patient Journey and Clinical Care
Source: Int J Vasc Med. 2026 Jun 24;2026:8034303. doi: 10.1155/ijvm/8034303 (PMC13291890; doi:10.1155/ijvm/8034303)
Supplement: Supplementary file 3 — Supporting Information 3 File S3: Venous clinical severity score (VCSS). [file IJVM-2026-8034303-s003.docx]

# Supplementary file 3: Venous Clinical Severity Score (revised) (Source: Vasquez et al., 2010)

|  | None (0) | Mild (1) | Moderate (2) | Severe (3) |
| --- | --- | --- | --- | --- |
| Pain or other discomfort (ie, aching, heaviness, fatigue, soreness, burning). |  | Occasional pain or other discomfort (ie, not restricting regular daily activities) | Daily pain or other discomfort (ie, interfering with but not preventing regular daily activities) | Daily pain or discomfort (ie, limits most regular daily activities) |
| Varicose veins (≥3 mm in diameter to qualify in the standing position) |  | Few: scattered (ie, isolated branch varicosities or clusters).  Also includes corona phlebectatica (ankle flare) | Confined to calf or thigh | Involves calf and thigh |
| Venous oedema |  | Limited to foot and ankle area | Extends above ankle but below knee | Extends to knee and above |
| Skin pigmentation (Does not include focal pigmentation over varicose veins or pigmentation due to other chronic diseases) | None or focal | Limited to perimalleolar area | Diffuse over lower third of calf | Wider distribution above lower third of calf |
| Inflammation (ie, erythema, cellulitis, venous eczema, dermatitis) |  | Limited to perimalleolar area | Diffuse over lower third of calf | Wider distribution above lower third of calf |
| Induration (Presumes venous origin of secondary skin and subcutaneous changes (ie, chronic edema with fibrosis, hypodermitis). Includes white atrophy and lipodermatosclerosis) |  | Limited to perimalleolar area | Diffuse over lower third of calf | Wider distribution above lower third of calf |
| Active ulcer number | 0 | 1 | 2 | >3 |
| Active ulcer duration (longest active) | n/a | <3 months | >3 months but <1 y | Not healed for >1 y |
| Active ulcer size (largest active) |  | Diameter <2 cm | Diameter 2-6 cm | Diameter >6 cm |
| Use of compression therapy | Not used | Intermittent use of stockings | Wears stockings most days | Full compliance: stockings |
