# Supplementary material for: A Systematic Review of the Effectiveness of Assessing Skin Changes for Chronic Venous Insufficiency in People With Dark Skin Tones and the Impact on the Patient Journey and Clinical Care
Source: Int J Vasc Med. 2026 Jun 24;2026:8034303. doi: 10.1155/ijvm/8034303 (PMC13291890; doi:10.1155/ijvm/8034303)
Supplement: Supplementary file 4 — Supporting Information 4 File S4: Search strategy (4a) and search overview (4b) by database. [file IJVM-2026-8034303-s004.docx]

# Supplementary file 4: Search strategy

4a) Overview of search

| Population(1): | Individuals with chronic venous insufficiency (CVI). | venous disease, venous insufficiency, leg ulcer, venous ulcer, varicose ulcer, venous eczema, lipodermatosclerosis, atrophie blanche, h(a)emosiderin staining, h(a)emosiderin pigmentation, corona phlebectatica, venous staining, cellulitis, erysipelas, varicose veins, truncal reflux, telangiectasia, reticular veins, thread veins, truncal veins, ankle flare, CEAP, leg *[close to*]* (o)edema , limb *[close to*]* (o)edema, leg *[close to*]* hyperpigmentation, limb *[close to*]* hyperpigmentation, leg *[close to*]* erythema, limb *[close to*]* erythema, leg *[close to*]* redness, limb *[close to*]* redness, leg *[close to*]* discolouration, limb *[close to*]* discolouration, leg *[close to*]* staining, limb *[close to*]* staining |
| --- | --- | --- |
| Population (2): | People with dark skin tones (which includes where ethnicity/race is reported without reporting skin tone specifically) | dark skin, darker skin, ethnic skin, skin of colo(u)r, people of colo(u)r, skin pigmentation, black skin, brown skin, olive skin, Asian people, Arab people, Black people, African people, African American people, Hispanic people, Latino people, Hawaiian people,  Pacific Islanders, Aboriginal, Oceanic ancestry, Native American people, Afro Caribbean people, Indigenous people, Indian people. Fitzpatrick skin phototype, skin tones chart, colo(u)r bar chart, munsell chart |
| Intervention | Assessment of skin changes associated with chronic venous | Skin assessment, Tissue assessment, Nursing assessment, Physical assessment, Vascular assessment, Lower limb assessment, Doppler assessment, ABPI, Duplex, Risk assessment, CEAP Classification, Doppler |
| Outcome | The effectiveness of assessment methods and impact this has on patient journey and clinical care. | Accuracy, Efficacy, Effectiveness, Validity, Reliability, Differential diagnosis, Diagnosis, Timely diagnosis, Prompt diagnosis, Early diagnosis, Delayed diagnosis, Referral, Patient care, Patient journey, Patient trajectory, Patient outcomes, Clinical outcomes |

4b) Searches performed by database.

All searches were last performed in each database and registry on 4^th^ March 2025.

| ClinicalTrails.Gov | Conditions or disease : "venous disease" OR "venous insufficiency" OR "leg ulcer" OR "venous ulcer" OR "varicose ulcer" OR "venous eczema" OR "lipodermatosclerosis" OR "atrophie blanche" OR hemosiderin staining OR hemosiderin pigmentation OR "corona phlebectatica" OR "venous staining" OR "cellulitis" OR "erysipelas" OR "varicose veins" OR "truncal reflux" OR "telangiectasia" OR "reticular veins" OR "thread veins" OR "truncal veins" OR "ankle flare" OR "CEAP" OR leg edema OR limb edema leg N/3 hyperpigmentation OR limb N/3 hyperpigmentation OR leg erythema OR limb erythema OR leg redness OR limb redness OR leg discoloration OR limb discoloration OR leg staining OR limb staining  With terms: ("dark skin" OR "darker skin" OR "ethnic skin" OR “skin of color” OR “people of color” OR "skin pigmentation" OR "black skin" OR "brown skin" OR "olive skin" OR "Asian people" OR "Asians" OR "Arab people" OR "Arabs" OR "Black people" OR "Blacks" OR "African people" OR "Africans" OR "African American people" OR "African Americans" OR "Hispanic people" OR "Hispanics" OR "Latino people" OR "Latinos" OR "Hawaiian people" OR "Hawaiians" OR "Pacific islanders" OR "Aboriginal" OR "Oceanic ancestry" OR "Native American people" OR "Native Americans" OR “Afro-Caribbean people” OR “Afro-Caribbeans” OR "Indigenous people" OR "Indian people" OR "Indians" OR "Fitzpatrick skin phototype" OR "Fitzpatrick skin type" OR "skin tones chart" OR “color bar chart” OR “munsell color chart”)  AND ( "skin assessment" OR "tissue assessment" OR "nursing assessment" OR "physical assessment" OR "vascular assessment" OR "lower limb assessment" OR "doppler assessment" OR "ABPI" OR "duplex" OR "risk assessment" OR "CEAP classification" OR "doppler" ) |
| --- | --- |
| ISRCTN registry | (("dark skin") OR ("darker skin") OR ("ethnic skin") OR (skin of color) OR (people of color) OR ("skin pigmentation") OR ("black skin") OR ("brown skin") OR ("olive skin") OR (Fitzpatrick skin)  OR ("skin tones chart") OR (color bar chart) OR (munsell color chart) OR (Asian*) OR (Arab*) OR (Black people) OR (Blacks) OR (African*) OR (African American*) OR (Hispanic*) OR (Latino*) OR (Hawaiian*) OR OR (Pacific islanders) OR (Aboriginal*) OR (Oceanic ancestry) OR (Native American*) OR (Afro Caribbean*) OR (Indigenous people) OR (Indian*)) AND ((venous disease) OR ("venous insufficiency") OR ("leg ulcer") OR ("venous ulcer") OR ("varicose ulcer") OR ("venous eczema") OR ("lipodermatosclerosis"))   OR  (("dark skin") OR ("darker skin") OR ("ethnic skin") OR (skin of color) OR (people of color) OR ("skin pigmentation") OR ("black skin") OR ("brown skin") OR ("olive skin") OR (Fitzpatrick skin)  OR ("skin tones chart") OR (color bar chart) OR (munsell color chart) OR (Asian*) OR (Arab*) OR (Black people) OR (Blacks) OR (African*) OR (African American*) OR (Hispanic*) OR (Latino*) OR (Hawaiian*) OR OR (Pacific islanders) OR (Aboriginal*) OR (Oceanic ancestry) OR (Native American*) OR (Afro Caribbean*) OR (Indigenous people) OR (Indian*)) AND (("atrophie blanche") OR (haemosiderin) OR (hemosiderin) OR ("corona phlebectatica") OR ("venous staining") OR (cellulitis) OR (erysipelas) OR ("varicose veins"))   OR  (("dark skin") OR ("darker skin") OR ("ethnic skin") OR (skin of color) OR (people of color) OR ("skin pigmentation") OR ("black skin") OR ("brown skin") OR ("olive skin") OR (Fitzpatrick skin)  OR ("skin tones chart") OR (color bar chart) OR (munsell color chart) OR (Asian*) OR (Arab*) OR (Black people) OR (Blacks) OR (African*) OR (African American*) OR (Hispanic*) OR (Latino*) OR (Hawaiian*) OR OR (Pacific islanders) OR (Aboriginal*) OR (Oceanic ancestry) OR (Native American*) OR (Afro Caribbean*) OR (Indigenous people) OR (Indian*)) AND (("truncal reflux") OR (telangiectasia) OR ("reticular veins") OR ("thread veins") OR ("truncal veins") OR ("ankle flare") OR (CEAP) OR (leg edema) OR (limb edema))   OR  (("dark skin") OR ("darker skin") OR ("ethnic skin") OR (skin of color) OR (people of color) OR ("skin pigmentation") OR ("black skin") OR ("brown skin") OR ("olive skin") OR (Fitzpatrick skin)  OR ("skin tones chart") OR (color bar chart) OR (munsell color chart) OR (Asian*) OR (Arab*) OR (Black people) OR (Blacks) OR (African*) OR (African American*) OR (Hispanic*) OR (Latino*) OR (Hawaiian*) OR OR (Pacific islanders) OR (Aboriginal*) OR (Oceanic ancestry) OR (Native American*) OR (Afro Caribbean*) OR (Indigenous people) OR (Indian*)) AND ((leg oedema) OR (limb oedema) OR (leg hyper-pigmentation) OR (limb hyper-pigmentation) OR (leg erythema) OR (limb erythema) OR (leg redness) OR (limb redness))   OR  (("dark skin") OR ("darker skin") OR ("ethnic skin") OR (skin of color) OR (people of color) OR ("skin pigmentation") OR ("black skin") OR ("brown skin") OR ("olive skin") OR (Fitzpatrick skin)  OR ("skin tones chart") OR (color bar chart) OR (munsell color chart) OR (Asian*) OR (Arab*) OR (Black people) OR (Blacks) OR (African*) OR (African American*) OR (Hispanic*) OR (Latino*) OR (Hawaiian*) OR OR (Pacific islanders) OR (Aboriginal*) OR (Oceanic ancestry) OR (Native American*) OR (Afro Caribbean*) OR (Indigenous people) OR (Indian*)) AND ((leg discoloration) OR (limb discoloration) OR (leg discolouration) OR (limb discolouration) OR (leg staining) OR (limb staining)) |
| British Nursing Index | "dark skin" OR "darker skin" OR "ethnic skin" OR skin of colo?r OR people of colo?r OR "skin pigmentation" OR "black skin" OR "brown skin" OR "olive skin" OR "Asian people" OR "Asians" OR "Arab people" OR "Arabs" OR "Black people" OR "Blacks" OR "African people" OR "Africans" OR "African American people" OR "African Americans" OR "Hispanic people" OR "Hispanics" OR "Latino people" OR "Latinos" OR "Hawaiian people" OR "Hawaiians" OR "Pacific islanders" OR "Aboriginal" OR "Oceanic ancestry" OR "Native American people" OR "Native Americans" OR Afro?Caribbean people OR Afro?Caribbeans OR "Indigenous people" OR "Indian people" OR "Indians" OR "Fitzpatrick skin phototype" OR "Fitzpatrick skin type" OR "skin tones chart" OR colo?r bar chart OR munsell colo?r chart OR MAINSUBJECT.EXACT("Hmong people") OR MAINSUBJECT.EXACT("Arab people") OR MAINSUBJECT.EXACT("Black people") OR MAINSUBJECT.EXACT("Asian people") OR MAINSUBJECT.EXACT("Native peoples") OR MAINSUBJECT.EXACT("Rohingya people") OR MAINSUBJECT.EXACT("Maroons (People)") OR MAINSUBJECT.EXACT("Black British people") OR MAINSUBJECT.EXACT("Pacific Islander people") OR MAINSUBJECT.EXACT("Palestinian people") OR MAINSUBJECT.EXACT("Multiracial people") OR MAINSUBJECT.EXACT("British Asian people") OR MAINSUBJECT.EXACT("Hispanic people") OR MAINSUBJECT.EXACT("Uyghur people")  AND  "venous disease" OR "venous insufficiency" OR "leg ulcer" OR "venous ulcer" OR "varicose ulcer" OR "venous eczema" OR "lipodermatosclerosis" OR "atrophie blanche" OR h?emosiderin staining OR h?emosiderin pigmentation OR "corona phlebectatica" OR "venous staining" OR "cellulitis" OR "erysipelas" OR "varicose veins" OR "truncal reflux" OR "telangiectasia" OR "reticular veins" OR "thread veins" OR "truncal veins" OR "ankle flare" OR "CEAP" OR leg N/3 edema OR limb N/3 edema OR leg N/3 oedema OR limb N/3 oedema OR leg N/3 hyper?pigmentation OR limb N/3 hyper?pigmentation OR leg N/3 erythema OR limb N/3 erythema OR leg N/3 redness OR limb N/3 redness OR leg N/3 discolo?ration OR limb N/3 discolo?ration OR leg N/3 staining OR limb N/3 staining OR MAINSUBJECT.EXACT("Leg ulcers") OR MAINSUBJECT.EXACT("Cellulitis") OR MAINSUBJECT.EXACT("Varicose veins")  AND  “skin assessment” OR “tissue assessment” OR “nursing assessment” OR “physical assessment” OR “vascular assessment” OR “lower limb assessment” OR “doppler assessment” OR “ABPI” OR “duplex” OR “risk assessment” OR “CEAP classification” OR “doppler” OR MAINSUBJECT.EXACT("Patient assessment") OR MAINSUBJECT.EXACT("Risk assessment")  AND  “accuracy” OR “efficacy” OR “effectiveness” OR “reliability” OR “validity” OR “differential diagnosis” OR “diagnosis” OR “timely diagnosis” OR “prompt diagnosis” OR “early diagnosis” OR “delayed diagnosis” OR “referral” OR “patient care” OR “patient journey” OR “patient trajectory” OR “patient outcomes” OR “clinical outcomes” OR MAINSUBJECT.EXACT("Accuracy") OR MAINSUBJECT.EXACT("Efficiency") OR MAINSUBJECT.EXACT("Effectiveness studies") OR MAINSUBJECT.EXACT("Effectiveness") OR MAINSUBJECT.EXACT("Reliability") OR MAINSUBJECT.EXACT("Validity") OR MAINSUBJECT.EXACT("Diagnostic tests") OR MAINSUBJECT.EXACT("Physical examinations") OR MAINSUBJECT.EXACT("Medical diagnosis") OR MAINSUBJECT.EXACT("Medical referrals") OR MAINSUBJECT.EXACT("Referrals") OR MAINSUBJECT.EXACT("Recovery (Medical)") OR MAINSUBJECT.EXACT("Health informatics") OR MAINSUBJECT.EXACT("Clinical outcomes") |
| Scopus | (TITLE-ABS-KEY ( "dark skin" OR "darker skin" OR "ethnic skin" OR "skin of colo?r" OR "skin of colour" OR "people of color" OR "people of colour" OR "skin pigmentation" OR "black skin" OR "brown skin" OR "olive skin" OR "Asian people" OR "Asians" OR "Arab people" OR "Arabs" OR "Black people" OR "Blacks" OR "African people" OR "Africans" OR "African American people" OR "African Americans" OR "Hispanic people" OR "Hispanics" OR "Latino people" OR "Latinos" OR "Hawaiian people" OR "Hawaiians" OR "Pacific islanders" OR "Aboriginal" OR "Oceanic ancestry" OR "Native American people" OR "Native Americans" OR "Afro?Caribbean people" OR "Afro?Caribbeans" OR "Indigenous people" OR "Indian people" OR "Indians" OR "Fitzpatrick skin phototype" OR "Fitzpatrick skin type" OR "skin tones chart" OR "colo?r bar chart" OR "munsell colo?r chart" ) AND TITLE-ABS-KEY ( "venous disease" OR "venous insufficiency" OR "leg ulcer" OR "venous ulcer" OR "varicose ulcer" OR "venous eczema" OR "lipodermatosclerosis" OR "atrophie blanche" OR "h?emosiderin staining" OR "h?emosiderin pigmentation" OR "corona phlebectatica" OR "venous staining" OR "cellulitis" OR "erysipelas" OR "varicose veins" OR "truncal reflux" OR "telangiectasia" OR "reticular veins" OR "thread veins" OR "truncal veins" OR "ankle flare" OR "CEAP" OR "leg W/3 ?edema" OR "limb W/3 ?edema" OR "leg W/3 hyper?pigmentation" OR "limb W/3 hyper?pigmentation" OR "leg W/3 erythema" OR "limb W/3 erythema" OR "leg W/3 redness" OR "limb W/3 redness" OR "leg W/3 discolo?ration" OR "limb W/3 discolo?ration" OR "leg W/3 staining" OR "limb W/3 staining" ) AND ALL ( "skin assessment" OR "tissue assessment" OR "nursing assessment" OR "physical assessment" OR "vascular assessment" OR "lower limb assessment" OR "doppler assessment" OR "ABPI" OR "duplex" OR "risk assessment" OR "CEAP classification" OR "doppler" ) AND ALL ( "accuracy" OR "efficacy" OR "effectiveness" OR "reliability" OR "validity" OR "differential diagnosis" OR "diagnosis" OR "timely diagnosis" OR "prompt diagnosis" OR "early diagnosis" OR "delayed diagnosis" OR "referral" OR "patient care" OR "patient journey" OR "patient trajectory" OR "patient outcomes" OR "clinical outcomes" ) ) |
| CINHAL | \| S97 \| S37 AND S66 AND S80 AND S96 \| \| --- \| --- \| \| S96 \| S81 OR S82 OR S83 OR S84 OR S85 OR S86 OR S87 OR S88 OR S89 OR S90 OR S91 OR S92 OR S93 OR S94 OR S95 \| \| S95 \| (MH "Outcome Assessment") OR "Clinical outcomes" \| \| S94 \| (MH "Patient-Reported Outcomes") OR "Patient outcomes" \| \| S93 \| "Patient trajectory" \| \| S92 \| "Patient journey" \| \| S91 \| (MH "Patient Care") OR "Patient care" \| \| S90 \| (MH "Referral and Consultation") OR "Referral" \| \| S89 \| (MH "Diagnosis, Delayed") OR "Delayed diagnosis" \| \| S88 \| (MH "Early Diagnosis") OR "Early diagnosis" \| \| S87 \| "Prompt diagnosis" \| \| S86 \| "Timely diagnosis" OR (MH "Failure to Diagnose") \| \| S85 \| (MH "Diagnosis") OR "Diagnosis" \| \| S84 \| (MH "Diagnosis, Differential") OR "Differential diagnosis" \| \| S83 \| "Effectiveness" \| \| S82 \| "Efficacy" \| \| S81 \| (MH "Reliability") OR "Accuracy" \| \| S80 \| S67 OR S68 OR S69 OR S70 OR S71 OR S72 OR S73 OR S74 OR S75 OR S76 OR S77 OR S78 OR S79 \| \| S79 \| (MH "Ankle Brachial Index") \| \| S78 \| (MH "Ultrasonography, Doppler") OR "Doppler" \| \| S77 \| "CEAP Classification" \| \| S76 \| (MH "Risk Assessment") OR "Risk assessment" \| \| S75 \| (MH "Ultrasonography, Doppler, Duplex") OR "Duplex" \| \| S74 \| "ABPI" \| \| S73 \| "Doppler assessment" \| \| S72 \| "Lower limb assessment" \| \| S71 \| "Vascular assessment" \| \| S70 \| (MH "Physical Examination") OR "physical assessment" \| \| S69 \| (MH "Nursing Assessment") OR "Nursing assessment" OR (MH "Patient Assessment") \| \| S68 \| "Tissue assessment" \| \| S67 \| (MH "Clinical Assessment Tools") OR (MH "Wound Assessment") OR "Skin assessment" OR (MH "Physical Examination") \| \| S66 \| S38 OR S39 OR S40 OR S41 OR S42 OR S43 OR S44 OR S45 OR S46 OR S47 OR S48 OR S49 OR S50 OR S51 OR S52 OR S53 OR S54 OR S55 OR S56 OR S57 OR S58 OR S59 OR S60 OR S61 OR S62 OR S63 OR S64 OR S65 \| \| S65 \| "munsell colour chart" \| \| S64 \| "colour bar chart" \| \| S63 \| "skin tones chart" \| \| S62 \| "Fitzpatrick skin phototype" OR (MH "Skin Pigmentation") \| \| S61 \| "Indian people" OR (MH "Southeast Asians") OR (MH "East Asians") OR (MH "Middle Eastern Persons") OR (MH "East Africans") \| \| S60 \| (MH "Indigenous Peoples") OR "Indigenous people" \| \| S59 \| (MH "Caribbean Persons") OR "Afro Caribbean people" \| \| S58 \| (MH "Native Americans") OR "Native American people" \| \| S57 \| "Oceanic ancestry" \| \| S56 \| (MH "Aboriginal Australians") OR (MH "Aboriginal Canadians") OR (MH "First Nations of Australia") OR (MH "Native Americans") OR (MH "Indigenous Peoples") OR "Aboriginal" \| \| S55 \| (MH "Pacific Islanders") OR "Pacific Islanders" \| \| S54 \| (MH "Pacific Islanders") OR "Hawaiian people" OR (MH "First Nations of Australia") \| \| S53 \| "Latino people" \| \| S52 \| "Hispanic people" OR (MH "Hispanic Americans") \| \| S51 \| (MH "African Americans") OR "African American people" OR (MH "Native Americans") \| \| S50 \| (MH "West Africans") OR (MH "Central Africans") OR (MH "East Africans") OR (MH "Southern Africans") OR (MH "Africans") OR "African people" \| \| S49 \| (MH "Black Persons") OR (MH "Indigenous Peoples") OR "Black people" \| \| S48 \| (MH "Arabs") OR (MH "Arctic Peoples") OR (MH "Indigenous Peoples") OR (MH "Pacific Islanders") OR (MH "Aboriginal Canadians") OR (MH "First Nations of Australia") OR "Arab people" OR (MH "Navajo Persons") OR (MH "Pima Persons") OR (MH "West Africans") OR (MH "Southeast Asians") OR (MH "South Asians") OR (MH "North Korea") OR (MH "Middle Eastern Persons") OR (MH "East Asians") OR (MH "East Africans") OR (MH "Central Africans") OR (MH "Southern Africans") OR (MH "Maori") OR (MH "Caribbean Persons") OR (MH "Central Americans") OR (MH "Central Asians") OR (MH "Laotians") OR (MH "Hmong") OR (MH "Aboriginal Australians") OR (MH "Africans") \| \| S47 \| (MH "Central Asians") OR (MH "Middle Eastern Persons") OR (MH "Southeast Asians") OR (MH "East Asians") OR (MH "South Asians") OR (MH "Asians") OR (MH "Arctic Peoples") OR "Asian people" \| \| S46 \| "olive skin" \| \| S45 \| "brown skin" \| \| S44 \| (MH "Black Persons") OR "black skin" \| \| S43 \| (MH "Skin Pigmentation") OR "skin pigmentation" \| \| S42 \| people of color \| \| S41 \| skin of colo#r \| \| S40 \| "ethnic skin" \| \| S39 \| (MH "Skin Pigmentation") OR "darker skin" \| \| S38 \| "dark skin" \| \| S37 \| S1 OR S2 OR S3 OR S4 OR S5 OR S6 OR S7 OR S8 OR S9 OR S10 OR S11 OR S12 OR S13 OR S14 OR S15 OR S16 OR S17 OR S18 OR S19 OR S20 OR S21 OR S22 OR S23 OR S24 OR S25 OR S26 OR S27 OR S28 OR S29 OR S30 OR S31 OR S32 OR S33 OR S34 OR S35 OR S36 \| \| S36 \| h#emosiderin pigmentation \| \| S35 \| h#emosiderin staining \| \| S34 \| limb N3 staining \| \| S33 \| leg N3 staining \| \| S32 \| limb N3 discolouration \| \| S31 \| leg N3 discolouration \| \| S30 \| leg N3 redness \| \| S29 \| limb N3 redness \| \| S28 \| limb N3 erythema \| \| S27 \| leg N3 erythema \| \| S26 \| limb N3 hyperpigmentation \| \| S25 \| leg N3 hyperpigmentation \| \| S24 \| limb N3 edema \| \| S23 \| limb N3 oedema \| \| S22 \| leg N3 edema \| \| S21 \| leg N3 oedema \| \| S20 \| "CEAP" \| \| S19 \| "ankle flare" \| \| S18 \| "truncal veins" \| \| S17 \| "thread veins" \| \| S16 \| "reticular veins" \| \| S15 \| "telangiectasia" \| \| S14 \| "truncal reflux" \| \| S13 \| (MH "Varicose Veins") OR "varicose veins" \| \| S12 \| "erysipelas" \| \| S11 \| (MH "Cellulitis") OR "cellulitis" \| \| S10 \| "venous staining" \| \| S9 \| "corona phlebectatica" \| \| S8 \| "atrophie blanche" \| \| S7 \| "lipodermatosclerosis" \| \| S6 \| "venous eczema" \| \| S5 \| "varicose ulcer*" \| \| S4 \| (MH "Venous Ulcer") OR "venous ulcer*" \| \| S3 \| (MH "Leg Ulcer") OR (MH "Venous Ulcer") OR "leg ulcer*" \| \| S2 \| (MH "Venous Insufficiency") OR "venous insufficiency" \| \| S1 \| "venous disease" \| |
| MedLine | \| 1. \| exp Skin Pigmentation/ \| \| --- \| --- \| \| 2. \| skin pigmentation.mp. \| \| 3. \| Dark skin.mp. \| \| 4. \| Darker skin.mp. [mp=title, book title, abstract, original title, name of substance word, subject heading word, floating sub-heading word, keyword heading word, organism supplementary concept word, protocol supplementary concept word, rare disease supplementary concept word, unique identifier, synonyms, population supplementary concept word, anatomy supplementary concept word] \| \| 5. \| Skin of colo?r.mp. [mp=title, book title, abstract, original title, name of substance word, subject heading word, floating sub-heading word, keyword heading word, organism supplementary concept word, protocol supplementary concept word, rare disease supplementary concept word, unique identifier, synonyms, population supplementary concept word, anatomy supplementary concept word] \| \| 6. \| people of colo?r.mp. [mp=title, book title, abstract, original title, name of substance word, subject heading word, floating sub-heading word, keyword heading word, organism supplementary concept word, protocol supplementary concept word, rare disease supplementary concept word, unique identifier, synonyms, population supplementary concept word, anatomy supplementary concept word] \| \| 7. \| Brown skin.mp. [mp=title, book title, abstract, original title, name of substance word, subject heading word, floating sub-heading word, keyword heading word, organism supplementary concept word, protocol supplementary concept word, rare disease supplementary concept word, unique identifier, synonyms, population supplementary concept word, anatomy supplementary concept word] \| \| 8. \| Olive skin.mp. [mp=title, book title, abstract, original title, name of substance word, subject heading word, floating sub-heading word, keyword heading word, organism supplementary concept word, protocol supplementary concept word, rare disease supplementary concept word, unique identifier, synonyms, population supplementary concept word, anatomy supplementary concept word] \| \| 9. \| exp Ethnicity/ \| \| 10. \| exp Black People/ \| \| 11. \| exp "Black or African American"/ \| \| 12. \| exp Caribbean People/ \| \| 13. \| exp North African People/ \| \| 14. \| exp Middle Eastern People/ \| \| 15. \| exp Indians, South American/ or exp South American People/ \| \| 16. \| exp Asian People/ \| \| 17. \| (black or african american).mp. [mp=title, book title, abstract, original title, name of substance word, subject heading word, floating sub-heading word, keyword heading word, organism supplementary concept word, protocol supplementary concept word, rare disease supplementary concept word, unique identifier, synonyms, population supplementary concept word, anatomy supplementary concept word] \| \| 18. \| arabs.mp. [mp=title, book title, abstract, original title, name of substance word, subject heading word, floating sub-heading word, keyword heading word, organism supplementary concept word, protocol supplementary concept word, rare disease supplementary concept word, unique identifier, synonyms, population supplementary concept word, anatomy supplementary concept word] \| \| 19. \| asian american.mp. [mp=title, book title, abstract, original title, name of substance word, subject heading word, floating sub-heading word, keyword heading word, organism supplementary concept word, protocol supplementary concept word, rare disease supplementary concept word, unique identifier, synonyms, population supplementary concept word, anatomy supplementary concept word] \| \| 20. \| native hawaiian.mp. [mp=title, book title, abstract, original title, name of substance word, subject heading word, floating sub-heading word, keyword heading word, organism supplementary concept word, protocol supplementary concept word, rare disease supplementary concept word, unique identifier, synonyms, population supplementary concept word, anatomy supplementary concept word] \| \| 21. \| pacific islander.mp. [mp=title, book title, abstract, original title, name of substance word, subject heading word, floating sub-heading word, keyword heading word, organism supplementary concept word, protocol supplementary concept word, rare disease supplementary concept word, unique identifier, synonyms, population supplementary concept word, anatomy supplementary concept word] \| \| 22. \| hispanic.mp. [mp=title, book title, abstract, original title, name of substance word, subject heading word, floating sub-heading word, keyword heading word, organism supplementary concept word, protocol supplementary concept word, rare disease supplementary concept word, unique identifier, synonyms, population supplementary concept word, anatomy supplementary concept word] \| \| 23. \| latino.mp. [mp=title, book title, abstract, original title, name of substance word, subject heading word, floating sub-heading word, keyword heading word, organism supplementary concept word, protocol supplementary concept word, rare disease supplementary concept word, unique identifier, synonyms, population supplementary concept word, anatomy supplementary concept word] \| \| 24. \| indigenous people.mp. [mp=title, book title, abstract, original title, name of substance word, subject heading word, floating sub-heading word, keyword heading word, organism supplementary concept word, protocol supplementary concept word, rare disease supplementary concept word, unique identifier, synonyms, population supplementary concept word, anatomy supplementary concept word] \| \| 25. \| exp African People/ \| \| 26. \| exp "Hispanic or Latino"/ \| \| 27. \| exp Arabs/ \| \| 28. \| Hawaiian people.mp. [mp=title, book title, abstract, original title, name of substance word, subject heading word, floating sub-heading word, keyword heading word, organism supplementary concept word, protocol supplementary concept word, rare disease supplementary concept word, unique identifier, synonyms, population supplementary concept word, anatomy supplementary concept word] \| \| 29. \| exp "Native Hawaiian or Other Pacific Islander"/ \| \| 30. \| exp "Australian Aboriginal and Torres Strait Islander Peoples"/ \| \| 31. \| exp Indians, North American/ \| \| 32. \| Aboriginal.mp. \| \| 33. \| Oceanic ancestry.mp. \| \| 34. \| Native American people.mp. \| \| 35. \| exp "American Indian or Alaska Native"/ \| \| 36. \| Afro Caribbean people.mp. [mp=title, book title, abstract, original title, name of substance word, subject heading word, floating sub-heading word, keyword heading word, organism supplementary concept word, protocol supplementary concept word, rare disease supplementary concept word, unique identifier, synonyms, population supplementary concept word, anatomy supplementary concept word] \| \| 37. \| Indian people.mp. [mp=title, book title, abstract, original title, name of substance word, subject heading word, floating sub-heading word, keyword heading word, organism supplementary concept word, protocol supplementary concept word, rare disease supplementary concept word, unique identifier, synonyms, population supplementary concept word, anatomy supplementary concept word] \| \| 38. \| Fitzpatrick skin phototype.mp. [mp=title, book title, abstract, original title, name of substance word, subject heading word, floating sub-heading word, keyword heading word, organism supplementary concept word, protocol supplementary concept word, rare disease supplementary concept word, unique identifier, synonyms, population supplementary concept word, anatomy supplementary concept word] \| \| 39. \| Skin tones chart.mp. [mp=title, book title, abstract, original title, name of substance word, subject heading word, floating sub-heading word, keyword heading word, organism supplementary concept word, protocol supplementary concept word, rare disease supplementary concept word, unique identifier, synonyms, population supplementary concept word, anatomy supplementary concept word] \| \| 40. \| Colo?r bar chart.mp. [mp=title, book title, abstract, original title, name of substance word, subject heading word, floating sub-heading word, keyword heading word, organism supplementary concept word, protocol supplementary concept word, rare disease supplementary concept word, unique identifier, synonyms, population supplementary concept word, anatomy supplementary concept word] \| \| 41. \| Munsell colo?r chart.mp. [mp=title, book title, abstract, original title, name of substance word, subject heading word, floating sub-heading word, keyword heading word, organism supplementary concept word, protocol supplementary concept word, rare disease supplementary concept word, unique identifier, synonyms, population supplementary concept word, anatomy supplementary concept word] \| \| 42. \| 1 or 2 or 3 or 4 or 5 or 6 or 7 or 8 or 9 or 10 or 11 or 12 or 13 or 14 or 15 or 16 or 17 or 18 or 19 or 20 or 21 or 22 or 23 or 24 or 25 or 26 or 27 or 28 or 29 or 30 or 31 or 32 or 33 or 34 or 35 or 36 or 37 or 38 or 39 or 40 or 41 \| \| 43. \| exp Venous Insufficiency/ \| \| 44. \| Venous Insufficiency.mp. [mp=title, book title, abstract, original title, name of substance word, subject heading word, floating sub-heading word, keyword heading word, organism supplementary concept word, protocol supplementary concept word, rare disease supplementary concept word, unique identifier, synonyms, population supplementary concept word, anatomy supplementary concept word] \| \| 45. \| Venous Disease.mp. [mp=title, book title, abstract, original title, name of substance word, subject heading word, floating sub-heading word, keyword heading word, organism supplementary concept word, protocol supplementary concept word, rare disease supplementary concept word, unique identifier, synonyms, population supplementary concept word, anatomy supplementary concept word] \| \| 46. \| exp Varicose Veins/ \| \| 47. \| Varicose Veins.mp. [mp=title, book title, abstract, original title, name of substance word, subject heading word, floating sub-heading word, keyword heading word, organism supplementary concept word, protocol supplementary concept word, rare disease supplementary concept word, unique identifier, synonyms, population supplementary concept word, anatomy supplementary concept word] \| \| 48. \| exp Leg Ulcer/ \| \| 49. \| exp Varicose Ulcer/ \| \| 50. \| Venous Ulcer.mp. [mp=title, book title, abstract, original title, name of substance word, subject heading word, floating sub-heading word, keyword heading word, organism supplementary concept word, protocol supplementary concept word, rare disease supplementary concept word, unique identifier, synonyms, population supplementary concept word, anatomy supplementary concept word] \| \| 51. \| Varicose ulcer.mp. [mp=title, book title, abstract, original title, name of substance word, subject heading word, floating sub-heading word, keyword heading word, organism supplementary concept word, protocol supplementary concept word, rare disease supplementary concept word, unique identifier, synonyms, population supplementary concept word, anatomy supplementary concept word] \| \| 52. \| Venous eczema.mp. [mp=title, book title, abstract, original title, name of substance word, subject heading word, floating sub-heading word, keyword heading word, organism supplementary concept word, protocol supplementary concept word, rare disease supplementary concept word, unique identifier, synonyms, population supplementary concept word, anatomy supplementary concept word] \| \| 53. \| Lipodermatosclerosis.mp. [mp=title, book title, abstract, original title, name of substance word, subject heading word, floating sub-heading word, keyword heading word, organism supplementary concept word, protocol supplementary concept word, rare disease supplementary concept word, unique identifier, synonyms, population supplementary concept word, anatomy supplementary concept word] \| \| 54. \| Atrophie blanche.mp. [mp=title, book title, abstract, original title, name of substance word, subject heading word, floating sub-heading word, keyword heading word, organism supplementary concept word, protocol supplementary concept word, rare disease supplementary concept word, unique identifier, synonyms, population supplementary concept word, anatomy supplementary concept word] \| \| 55. \| H?emosiderin staining.mp. [mp=title, book title, abstract, original title, name of substance word, subject heading word, floating sub-heading word, keyword heading word, organism supplementary concept word, protocol supplementary concept word, rare disease supplementary concept word, unique identifier, synonyms, population supplementary concept word, anatomy supplementary concept word] \| \| 56. \| Corona phlebectatica.mp. [mp=title, book title, abstract, original title, name of substance word, subject heading word, floating sub-heading word, keyword heading word, organism supplementary concept word, protocol supplementary concept word, rare disease supplementary concept word, unique identifier, synonyms, population supplementary concept word, anatomy supplementary concept word] \| \| 57. \| Venous staining.mp. [mp=title, book title, abstract, original title, name of substance word, subject heading word, floating sub-heading word, keyword heading word, organism supplementary concept word, protocol supplementary concept word, rare disease supplementary concept word, unique identifier, synonyms, population supplementary concept word, anatomy supplementary concept word] \| \| 58. \| (leg adj3 ?edema).mp. [mp=title, book title, abstract, original title, name of substance word, subject heading word, floating sub-heading word, keyword heading word, organism supplementary concept word, protocol supplementary concept word, rare disease supplementary concept word, unique identifier, synonyms, population supplementary concept word, anatomy supplementary concept word] \| \| 59. \| (limb adj3 ?edema).mp. [mp=title, book title, abstract, original title, name of substance word, subject heading word, floating sub-heading word, keyword heading word, organism supplementary concept word, protocol supplementary concept word, rare disease supplementary concept word, unique identifier, synonyms, population supplementary concept word, anatomy supplementary concept word] \| \| 60. \| (leg adj3 hyperpigmentation).mp. [mp=title, book title, abstract, original title, name of substance word, subject heading word, floating sub-heading word, keyword heading word, organism supplementary concept word, protocol supplementary concept word, rare disease supplementary concept word, unique identifier, synonyms, population supplementary concept word, anatomy supplementary concept word] \| \| 61. \| (limb adj3 Hyperpigmentation).mp. [mp=title, book title, abstract, original title, name of substance word, subject heading word, floating sub-heading word, keyword heading word, organism supplementary concept word, protocol supplementary concept word, rare disease supplementary concept word, unique identifier, synonyms, population supplementary concept word, anatomy supplementary concept word] \| \| 62. \| (leg adj3 erythema).mp. [mp=title, book title, abstract, original title, name of substance word, subject heading word, floating sub-heading word, keyword heading word, organism supplementary concept word, protocol supplementary concept word, rare disease supplementary concept word, unique identifier, synonyms, population supplementary concept word, anatomy supplementary concept word] \| \| 63. \| (limb adj3 erythema).mp. [mp=title, book title, abstract, original title, name of substance word, subject heading word, floating sub-heading word, keyword heading word, organism supplementary concept word, protocol supplementary concept word, rare disease supplementary concept word, unique identifier, synonyms, population supplementary concept word, anatomy supplementary concept word] \| \| 64. \| (leg adj3 redness).mp. [mp=title, book title, abstract, original title, name of substance word, subject heading word, floating sub-heading word, keyword heading word, organism supplementary concept word, protocol supplementary concept word, rare disease supplementary concept word, unique identifier, synonyms, population supplementary concept word, anatomy supplementary concept word] \| \| 65. \| (limb adj3 redness).mp. [mp=title, book title, abstract, original title, name of substance word, subject heading word, floating sub-heading word, keyword heading word, organism supplementary concept word, protocol supplementary concept word, rare disease supplementary concept word, unique identifier, synonyms, population supplementary concept word, anatomy supplementary concept word] \| \| 66. \| (leg adj3 discolo?ration).mp. [mp=title, book title, abstract, original title, name of substance word, subject heading word, floating sub-heading word, keyword heading word, organism supplementary concept word, protocol supplementary concept word, rare disease supplementary concept word, unique identifier, synonyms, population supplementary concept word, anatomy supplementary concept word] \| \| 67. \| (limb adj3 discolo?ration).mp. [mp=title, book title, abstract, original title, name of substance word, subject heading word, floating sub-heading word, keyword heading word, organism supplementary concept word, protocol supplementary concept word, rare disease supplementary concept word, unique identifier, synonyms, population supplementary concept word, anatomy supplementary concept word] \| \| 68. \| (leg adj3 staining).mp. [mp=title, book title, abstract, original title, name of substance word, subject heading word, floating sub-heading word, keyword heading word, organism supplementary concept word, protocol supplementary concept word, rare disease supplementary concept word, unique identifier, synonyms, population supplementary concept word, anatomy supplementary concept word] \| \| 69. \| (limb adj3 staining).mp. [mp=title, book title, abstract, original title, name of substance word, subject heading word, floating sub-heading word, keyword heading word, organism supplementary concept word, protocol supplementary concept word, rare disease supplementary concept word, unique identifier, synonyms, population supplementary concept word, anatomy supplementary concept word] \| \| 70. \| h?emosiderin pigmentation.mp. [mp=title, book title, abstract, original title, name of substance word, subject heading word, floating sub-heading word, keyword heading word, organism supplementary concept word, protocol supplementary concept word, rare disease supplementary concept word, unique identifier, synonyms, population supplementary concept word, anatomy supplementary concept word] \| \| 71. \| exp Hemosiderin/ \| \| 72. \| (leg adj3 erysipelas).mp. [mp=title, book title, abstract, original title, name of substance word, subject heading word, floating sub-heading word, keyword heading word, organism supplementary concept word, protocol supplementary concept word, rare disease supplementary concept word, unique identifier, synonyms, population supplementary concept word, anatomy supplementary concept word] \| \| 73. \| (limb adj3 erysipelas).mp. [mp=title, book title, abstract, original title, name of substance word, subject heading word, floating sub-heading word, keyword heading word, organism supplementary concept word, protocol supplementary concept word, rare disease supplementary concept word, unique identifier, synonyms, population supplementary concept word, anatomy supplementary concept word] \| \| 74. \| (leg adj3 Cellulitis).mp. [mp=title, book title, abstract, original title, name of substance word, subject heading word, floating sub-heading word, keyword heading word, organism supplementary concept word, protocol supplementary concept word, rare disease supplementary concept word, unique identifier, synonyms, population supplementary concept word, anatomy supplementary concept word] \| \| 75. \| (limb adj3 Cellulitis).mp. [mp=title, book title, abstract, original title, name of substance word, subject heading word, floating sub-heading word, keyword heading word, organism supplementary concept word, protocol supplementary concept word, rare disease supplementary concept word, unique identifier, synonyms, population supplementary concept word, anatomy supplementary concept word] \| \| 76. \| Telangiectasia.mp. [mp=title, book title, abstract, original title, name of substance word, subject heading word, floating sub-heading word, keyword heading word, organism supplementary concept word, protocol supplementary concept word, rare disease supplementary concept word, unique identifier, synonyms, population supplementary concept word, anatomy supplementary concept word] \| \| 77. \| exp Telangiectasis/ \| \| 78. \| Reticular veins.mp. [mp=title, book title, abstract, original title, name of substance word, subject heading word, floating sub-heading word, keyword heading word, organism supplementary concept word, protocol supplementary concept word, rare disease supplementary concept word, unique identifier, synonyms, population supplementary concept word, anatomy supplementary concept word] \| \| 79. \| Thread veins.mp. [mp=title, book title, abstract, original title, name of substance word, subject heading word, floating sub-heading word, keyword heading word, organism supplementary concept word, protocol supplementary concept word, rare disease supplementary concept word, unique identifier, synonyms, population supplementary concept word, anatomy supplementary concept word] \| \| 80. \| Truncal veins.mp. [mp=title, book title, abstract, original title, name of substance word, subject heading word, floating sub-heading word, keyword heading word, organism supplementary concept word, protocol supplementary concept word, rare disease supplementary concept word, unique identifier, synonyms, population supplementary concept word, anatomy supplementary concept word] \| \| 81. \| Ankle flare.mp. [mp=title, book title, abstract, original title, name of substance word, subject heading word, floating sub-heading word, keyword heading word, organism supplementary concept word, protocol supplementary concept word, rare disease supplementary concept word, unique identifier, synonyms, population supplementary concept word, anatomy supplementary concept word] \| \| 82. \| CEAP.mp. [mp=title, book title, abstract, original title, name of substance word, subject heading word, floating sub-heading word, keyword heading word, organism supplementary concept word, protocol supplementary concept word, rare disease supplementary concept word, unique identifier, synonyms, population supplementary concept word, anatomy supplementary concept word] \| \| 83. \| CEAP classification.mp. [mp=title, book title, abstract, original title, name of substance word, subject heading word, floating sub-heading word, keyword heading word, organism supplementary concept word, protocol supplementary concept word, rare disease supplementary concept word, unique identifier, synonyms, population supplementary concept word, anatomy supplementary concept word] \| \| 84. \| 43 or 44 or 45 or 46 or 47 or 48 or 49 or 50 or 51 or 52 or 53 or 54 or 55 or 56 or 57 or 58 or 59 or 60 or 61 or 62 or 63 or 64 or 65 or 66 or 67 or 68 or 69 or 70 or 71 or 72 or 73 or 74 or 75 or 76 or 77 or 78 or 79 or 80 or 81 or 82 or 83 \| \| 85. \| Skin assessment.mp. \| \| 86. \| exp Nursing Assessment/ \| \| 87. \| tissue assessment.mp. \| \| 88. \| nursing assessment.mp. [mp=title, book title, abstract, original title, name of substance word, subject heading word, floating sub-heading word, keyword heading word, organism supplementary concept word, protocol supplementary concept word, rare disease supplementary concept word, unique identifier, synonyms, population supplementary concept word, anatomy supplementary concept word] \| \| 89. \| exp Physical Examination/ \| \| 90. \| exp Clinical Competence/ \| \| 91. \| Vascular assessment.mp. \| \| 92. \| Lower limb assessment.mp. \| \| 93. \| Doppler assessment.mp. \| \| 94. \| exp Ultrasonography, Doppler/ \| \| 95. \| Ankle Brachial Index.mp. \| \| 96. \| exp Ankle Brachial Index/ \| \| 97. \| ABPI.mp. [mp=title, book title, abstract, original title, name of substance word, subject heading word, floating sub-heading word, keyword heading word, organism supplementary concept word, protocol supplementary concept word, rare disease supplementary concept word, unique identifier, synonyms, population supplementary concept word, anatomy supplementary concept word] \| \| 98. \| exp Ultrasonography, Doppler, Duplex/ \| \| 99. \| exp Risk Assessment/ \| \| 100. \| risk assessment.mp. [mp=title, book title, abstract, original title, name of substance word, subject heading word, floating sub-heading word, keyword heading word, organism supplementary concept word, protocol supplementary concept word, rare disease supplementary concept word, unique identifier, synonyms, population supplementary concept word, anatomy supplementary concept word] \| \| 101. \| CEAP Classification.mp. [mp=title, book title, abstract, original title, name of substance word, subject heading word, floating sub-heading word, keyword heading word, organism supplementary concept word, protocol supplementary concept word, rare disease supplementary concept word, unique identifier, synonyms, population supplementary concept word, anatomy supplementary concept word] \| \| 102. \| Physical assessment.mp. [mp=title, book title, abstract, original title, name of substance word, subject heading word, floating sub-heading word, keyword heading word, organism supplementary concept word, protocol supplementary concept word, rare disease supplementary concept word, unique identifier, synonyms, population supplementary concept word, anatomy supplementary concept word] \| \| 103. \| 85 or 86 or 87 or 88 or 89 or 90 or 91 or 92 or 93 or 94 or 95 or 96 or 97 or 98 or 99 or 100 or 101 or 102 \| \| 104. \| Accuracy.mp. [mp=title, book title, abstract, original title, name of substance word, subject heading word, floating sub-heading word, keyword heading word, organism supplementary concept word, protocol supplementary concept word, rare disease supplementary concept word, unique identifier, synonyms, population supplementary concept word, anatomy supplementary concept word] \| \| 105. \| Efficacy.mp. [mp=title, book title, abstract, original title, name of substance word, subject heading word, floating sub-heading word, keyword heading word, organism supplementary concept word, protocol supplementary concept word, rare disease supplementary concept word, unique identifier, synonyms, population supplementary concept word, anatomy supplementary concept word] \| \| 106. \| Effectiveness.mp. [mp=title, book title, abstract, original title, name of substance word, subject heading word, floating sub-heading word, keyword heading word, organism supplementary concept word, protocol supplementary concept word, rare disease supplementary concept word, unique identifier, synonyms, population supplementary concept word, anatomy supplementary concept word] \| \| 107. \| Differential diagnosis.mp. [mp=title, book title, abstract, original title, name of substance word, subject heading word, floating sub-heading word, keyword heading word, organism supplementary concept word, protocol supplementary concept word, rare disease supplementary concept word, unique identifier, synonyms, population supplementary concept word, anatomy supplementary concept word] \| \| 108. \| exp Diagnosis, Differential/ \| \| 109. \| Timely Diagnosis.mp. [mp=title, book title, abstract, original title, name of substance word, subject heading word, floating sub-heading word, keyword heading word, organism supplementary concept word, protocol supplementary concept word, rare disease supplementary concept word, unique identifier, synonyms, population supplementary concept word, anatomy supplementary concept word] \| \| 110. \| Prompt diagnosis.mp. [mp=title, book title, abstract, original title, name of substance word, subject heading word, floating sub-heading word, keyword heading word, organism supplementary concept word, protocol supplementary concept word, rare disease supplementary concept word, unique identifier, synonyms, population supplementary concept word, anatomy supplementary concept word] \| \| 111. \| Early diagnosis.mp. [mp=title, book title, abstract, original title, name of substance word, subject heading word, floating sub-heading word, keyword heading word, organism supplementary concept word, protocol supplementary concept word, rare disease supplementary concept word, unique identifier, synonyms, population supplementary concept word, anatomy supplementary concept word] \| \| 112. \| exp Diagnosis/ \| \| 113. \| exp Delayed Diagnosis/ \| \| 114. \| exp Early Diagnosis/ \| \| 115. \| exp Missed Diagnosis/ \| \| 116. \| exp Nursing Diagnosis/ \| \| 117. \| Delayed diagnosis.mp. [mp=title, book title, abstract, original title, name of substance word, subject heading word, floating sub-heading word, keyword heading word, organism supplementary concept word, protocol supplementary concept word, rare disease supplementary concept word, unique identifier, synonyms, population supplementary concept word, anatomy supplementary concept word] \| \| 118. \| Referral.mp. [mp=title, book title, abstract, original title, name of substance word, subject heading word, floating sub-heading word, keyword heading word, organism supplementary concept word, protocol supplementary concept word, rare disease supplementary concept word, unique identifier, synonyms, population supplementary concept word, anatomy supplementary concept word] \| \| 119. \| exp "Referral and Consultation"/ \| \| 120. \| exp Patient Care/ \| \| 121. \| Patient care.mp. [mp=title, book title, abstract, original title, name of substance word, subject heading word, floating sub-heading word, keyword heading word, organism supplementary concept word, protocol supplementary concept word, rare disease supplementary concept word, unique identifier, synonyms, population supplementary concept word, anatomy supplementary concept word] \| \| 122. \| exp "Delivery of Health Care"/ \| \| 123. \| Patient journey.mp. \| \| 124. \| Patient trajectory.mp. \| \| 125. \| Patient outcomes.mp. \| \| 126. \| Clinical outcomes.mp. \| \| 127. \| 104 or 105 or 106 or 107 or 108 or 109 or 110 or 111 or 112 or 113 or 114 or 115 or 116 or 117 or 118 or 119 or 120 or 121 or 122 or 123 or 124 or 125 or 126 \| \| 128. \| 42 and 84 and 103 and 127 \| |
| EMBASE | \| 1. \| exp Skin Pigmentation/ \|  \|  \| \|  \|  \| \| --- \| --- \| --- \| --- \| --- \| --- \| --- \| \| 2. \| skin pigmentation.mp. \|  \|  \| \|  \|  \| \| 3. \| Dark skin.mp. \|  \|  \| \|  \|  \| \| 4. \| Darker skin.mp. [mp=title, abstract, heading word, drug trade name, original title, device manufacturer, drug manufacturer, device trade name, keyword heading word, floating subheading word, candidate term word] \|  \|  \| \|  \|  \| \| 5. \| Skin of colo?r.mp. [mp=title, abstract, heading word, drug trade name, original title, device manufacturer, drug manufacturer, device trade name, keyword heading word, floating subheading word, candidate term word] \|  \|  \| \|  \|  \| \| 6. \| people of colo?r.mp. [mp=title, abstract, heading word, drug trade name, original title, device manufacturer, drug manufacturer, device trade name, keyword heading word, floating subheading word, candidate term word] \|  \|  \| \|  \|  \| \| 7. \| Brown skin.mp. [mp=title, abstract, heading word, drug trade name, original title, device manufacturer, drug manufacturer, device trade name, keyword heading word, floating subheading word, candidate term word] \|  \|  \| \|  \|  \| \| 8. \| Olive skin.mp. [mp=title, abstract, heading word, drug trade name, original title, device manufacturer, drug manufacturer, device trade name, keyword heading word, floating subheading word, candidate term word] \|  \|  \| \|  \|  \| \| 9. \| exp Ethnicity/ \|  \|  \| \|  \|  \| \| 10. \| exp Black People/ \|  \|  \| \|  \|  \| \| 11. \| exp "Black or African American"/ \|  \|  \| \|  \|  \| \| 12. \| exp Caribbean People/ \|  \|  \| \|  \|  \| \| 13. \| exp North African People/ \|  \|  \| \|  \|  \| \| 14. \| exp Middle Eastern People/ \|  \|  \| \|  \|  \| \| 15. \| exp Indians, South American/ or exp South American People/ \|  \|  \| \|  \|  \| \| 16. \| exp Asian People/ \|  \|  \| \|  \|  \| \| 17. \| (black or african american).mp. [mp=title, abstract, heading word, drug trade name, original title, device manufacturer, drug manufacturer, device trade name, keyword heading word, floating subheading word, candidate term word] \|  \|  \| \|  \|  \| \| 18. \| arabs.mp. [mp=title, abstract, heading word, drug trade name, original title, device manufacturer, drug manufacturer, device trade name, keyword heading word, floating subheading word, candidate term word] \|  \|  \| \|  \|  \| \| 19. \| asian american.mp. [mp=title, abstract, heading word, drug trade name, original title, device manufacturer, drug manufacturer, device trade name, keyword heading word, floating subheading word, candidate term word] \|  \|  \| \|  \|  \| \| 20. \| native hawaiian.mp. [mp=title, abstract, heading word, drug trade name, original title, device manufacturer, drug manufacturer, device trade name, keyword heading word, floating subheading word, candidate term word] \|  \|  \| \|  \|  \| \| 21. \| pacific islander.mp. [mp=title, abstract, heading word, drug trade name, original title, device manufacturer, drug manufacturer, device trade name, keyword heading word, floating subheading word, candidate term word] \|  \|  \| \|  \|  \| \| 22. \| hispanic.mp. [mp=title, abstract, heading word, drug trade name, original title, device manufacturer, drug manufacturer, device trade name, keyword heading word, floating subheading word, candidate term word] \|  \|  \| \|  \|  \| \| 23. \| latino.mp. [mp=title, abstract, heading word, drug trade name, original title, device manufacturer, drug manufacturer, device trade name, keyword heading word, floating subheading word, candidate term word] \|  \|  \| \|  \|  \| \| 24. \| indigenous people.mp. [mp=title, abstract, heading word, drug trade name, original title, device manufacturer, drug manufacturer, device trade name, keyword heading word, floating subheading word, candidate term word] \|  \|  \| \|  \|  \| \| 25. \| exp African People/ \|  \|  \| \|  \|  \| \| 26. \| exp "Hispanic or Latino"/ \|  \|  \| \|  \|  \| \| 27. \| exp Arabs/ \|  \|  \| \|  \|  \| \| 28. \| Hawaiian people.mp. [mp=title, abstract, heading word, drug trade name, original title, device manufacturer, drug manufacturer, device trade name, keyword heading word, floating subheading word, candidate term word] \|  \|  \| \|  \|  \| \| 29. \| exp "Native Hawaiian or Other Pacific Islander"/ \|  \|  \| \|  \|  \| \| 30. \| exp "Australian Aboriginal and Torres Strait Islander Peoples"/ \|  \|  \| \|  \|  \| \| 31. \| exp Indians, North American/ \|  \|  \| \|  \|  \| \| 32. \| Aboriginal.mp. \|  \|  \| \|  \|  \| \| 33. \| Oceanic ancestry.mp. \|  \|  \| \|  \|  \| \| 34. \| Native American people.mp. \|  \|  \| \|  \|  \| \| 35. \| exp "American Indian or Alaska Native"/ \|  \|  \| \|  \|  \| \| 36. \| Afro Caribbean people.mp. [mp=title, abstract, heading word, drug trade name, original title, device manufacturer, drug manufacturer, device trade name, keyword heading word, floating subheading word, candidate term word] \|  \|  \| \|  \|  \| \| 37. \| Indian people.mp. [mp=title, abstract, heading word, drug trade name, original title, device manufacturer, drug manufacturer, device trade name, keyword heading word, floating subheading word, candidate term word] \|  \|  \| \|  \|  \| \| 38. \| Fitzpatrick skin phototype.mp. [mp=title, abstract, heading word, drug trade name, original title, device manufacturer, drug manufacturer, device trade name, keyword heading word, floating subheading word, candidate term word] \|  \|  \| \|  \|  \| \| 39. \| Skin tones chart.mp. [mp=title, abstract, heading word, drug trade name, original title, device manufacturer, drug manufacturer, device trade name, keyword heading word, floating subheading word, candidate term word] \|  \|  \| \|  \|  \| \| 40. \| Colo?r bar chart.mp. [mp=title, abstract, heading word, drug trade name, original title, device manufacturer, drug manufacturer, device trade name, keyword heading word, floating subheading word, candidate term word] \|  \|  \| \|  \|  \| \| 41. \| Munsell colo?r chart.mp. [mp=title, abstract, heading word, drug trade name, original title, device manufacturer, drug manufacturer, device trade name, keyword heading word, floating subheading word, candidate term word] \|  \|  \| \|  \|  \| \| 42. \| 1 or 2 or 3 or 4 or 5 or 6 or 7 or 8 or 9 or 10 or 11 or 12 or 13 or 14 or 15 or 16 or 17 or 18 or 19 or 20 or 21 or 22 or 23 or 24 or 25 or 26 or 27 or 28 or 29 or 30 or 31 or 32 or 33 or 34 or 35 or 36 or 37 or 38 or 39 or 40 or 41 \|  \|  \| \|  \|  \| \| 43. \| exp Venous Insufficiency/ \|  \|  \| \|  \|  \| \| 44. \| Venous Insufficiency.mp. [mp=title, abstract, heading word, drug trade name, original title, device manufacturer, drug manufacturer, device trade name, keyword heading word, floating subheading word, candidate term word] \|  \|  \| \|  \|  \| \| 45. \| Venous Disease.mp. [mp=title, abstract, heading word, drug trade name, original title, device manufacturer, drug manufacturer, device trade name, keyword heading word, floating subheading word, candidate term word] \|  \|  \| \|  \|  \| \| 46. \| exp Varicose Veins/ \|  \|  \| \|  \|  \| \| 47. \| Varicose Veins.mp. [mp=title, abstract, heading word, drug trade name, original title, device manufacturer, drug manufacturer, device trade name, keyword heading word, floating subheading word, candidate term word] \|  \|  \| \|  \|  \| \| 48. \| exp Leg Ulcer/ \|  \|  \| \|  \|  \| \| 49. \| exp Varicose Ulcer/ \|  \|  \| \|  \|  \| \| 50. \| Venous Ulcer.mp. [mp=title, abstract, heading word, drug trade name, original title, device manufacturer, drug manufacturer, device trade name, keyword heading word, floating subheading word, candidate term word] \|  \|  \| \|  \|  \| \| 51. \| Varicose ulcer.mp. [mp=title, abstract, heading word, drug trade name, original title, device manufacturer, drug manufacturer, device trade name, keyword heading word, floating subheading word, candidate term word] \|  \|  \| \|  \|  \| \| 52. \| Venous eczema.mp. [mp=title, abstract, heading word, drug trade name, original title, device manufacturer, drug manufacturer, device trade name, keyword heading word, floating subheading word, candidate term word] \|  \|  \| \|  \|  \| \| 53. \| Lipodermatosclerosis.mp. [mp=title, abstract, heading word, drug trade name, original title, device manufacturer, drug manufacturer, device trade name, keyword heading word, floating subheading word, candidate term word] \|  \|  \| \|  \|  \| \| 54. \| Atrophie blanche.mp. [mp=title, abstract, heading word, drug trade name, original title, device manufacturer, drug manufacturer, device trade name, keyword heading word, floating subheading word, candidate term word] \|  \|  \| \|  \|  \| \| 55. \| H?emosiderin staining.mp. [mp=title, abstract, heading word, drug trade name, original title, device manufacturer, drug manufacturer, device trade name, keyword heading word, floating subheading word, candidate term word] \|  \|  \| \|  \|  \| \| 56. \| Corona phlebectatica.mp. [mp=title, abstract, heading word, drug trade name, original title, device manufacturer, drug manufacturer, device trade name, keyword heading word, floating subheading word, candidate term word] \|  \|  \| \|  \|  \| \| 57. \| Venous staining.mp. [mp=title, abstract, heading word, drug trade name, original title, device manufacturer, drug manufacturer, device trade name, keyword heading word, floating subheading word, candidate term word] \|  \|  \| \|  \|  \| \| 58. \| (leg adj3 ?edema).mp. [mp=title, abstract, heading word, drug trade name, original title, device manufacturer, drug manufacturer, device trade name, keyword heading word, floating subheading word, candidate term word] \|  \|  \| \|  \|  \| \| 59. \| (limb adj3 ?edema).mp. [mp=title, abstract, heading word, drug trade name, original title, device manufacturer, drug manufacturer, device trade name, keyword heading word, floating subheading word, candidate term word] \|  \|  \| \|  \|  \| \| 60. \| (leg adj3 hyperpigmentation).mp. [mp=title, abstract, heading word, drug trade name, original title, device manufacturer, drug manufacturer, device trade name, keyword heading word, floating subheading word, candidate term word] \|  \|  \| \|  \|  \| \| 61. \| (limb adj3 Hyperpigmentation).mp. [mp=title, abstract, heading word, drug trade name, original title, device manufacturer, drug manufacturer, device trade name, keyword heading word, floating subheading word, candidate term word] \|  \|  \| \|  \|  \| \| 62. \| (leg adj3 erythema).mp. [mp=title, abstract, heading word, drug trade name, original title, device manufacturer, drug manufacturer, device trade name, keyword heading word, floating subheading word, candidate term word] \|  \|  \| \|  \|  \| \| 63. \| (limb adj3 erythema).mp. [mp=title, abstract, heading word, drug trade name, original title, device manufacturer, drug manufacturer, device trade name, keyword heading word, floating subheading word, candidate term word] \|  \|  \| \|  \|  \| \| 64. \| (leg adj3 redness).mp. [mp=title, abstract, heading word, drug trade name, original title, device manufacturer, drug manufacturer, device trade name, keyword heading word, floating subheading word, candidate term word] \|  \|  \| \|  \|  \| \| 65. \| (limb adj3 redness).mp. [mp=title, abstract, heading word, drug trade name, original title, device manufacturer, drug manufacturer, device trade name, keyword heading word, floating subheading word, candidate term word] \|  \|  \| \|  \|  \| \| 66. \| (leg adj3 discolo?ration).mp. [mp=title, abstract, heading word, drug trade name, original title, device manufacturer, drug manufacturer, device trade name, keyword heading word, floating subheading word, candidate term word] \|  \|  \| \|  \|  \| \| 67. \| (limb adj3 discolo?ration).mp. [mp=title, abstract, heading word, drug trade name, original title, device manufacturer, drug manufacturer, device trade name, keyword heading word, floating subheading word, candidate term word] \|  \|  \| \|  \|  \| \| 68. \| (leg adj3 staining).mp. [mp=title, abstract, heading word, drug trade name, original title, device manufacturer, drug manufacturer, device trade name, keyword heading word, floating subheading word, candidate term word] \|  \|  \| \|  \|  \| \| 69. \| (limb adj3 staining).mp. [mp=title, abstract, heading word, drug trade name, original title, device manufacturer, drug manufacturer, device trade name, keyword heading word, floating subheading word, candidate term word] \|  \|  \| \|  \|  \| \| 70. \| h?emosiderin pigmentation.mp. [mp=title, abstract, heading word, drug trade name, original title, device manufacturer, drug manufacturer, device trade name, keyword heading word, floating subheading word, candidate term word] \|  \|  \| \|  \|  \| \| 71. \| exp Hemosiderin/ \|  \|  \| \|  \|  \| \| 72. \| (leg adj3 erysipelas).mp. [mp=title, abstract, heading word, drug trade name, original title, device manufacturer, drug manufacturer, device trade name, keyword heading word, floating subheading word, candidate term word] \|  \|  \| \|  \|  \| \| 73. \| (limb adj3 erysipelas).mp. [mp=title, abstract, heading word, drug trade name, original title, device manufacturer, drug manufacturer, device trade name, keyword heading word, floating subheading word, candidate term word] \|  \|  \| \|  \|  \| \| 74. \| (leg adj3 Cellulitis).mp. [mp=title, abstract, heading word, drug trade name, original title, device manufacturer, drug manufacturer, device trade name, keyword heading word, floating subheading word, candidate term word] \|  \|  \| \|  \|  \| \| 75. \| (limb adj3 Cellulitis).mp. [mp=title, abstract, heading word, drug trade name, original title, device manufacturer, drug manufacturer, device trade name, keyword heading word, floating subheading word, candidate term word] \|  \|  \| \|  \|  \| \| 76. \| Telangiectasia.mp. [mp=title, abstract, heading word, drug trade name, original title, device manufacturer, drug manufacturer, device trade name, keyword heading word, floating subheading word, candidate term word] \|  \|  \| \|  \|  \| \| 77. \| exp Telangiectasis/ \|  \|  \| \|  \|  \| \| 78. \| Reticular veins.mp. [mp=title, abstract, heading word, drug trade name, original title, device manufacturer, drug manufacturer, device trade name, keyword heading word, floating subheading word, candidate term word] \|  \|  \| \|  \|  \| \| 79. \| Thread veins.mp. [mp=title, abstract, heading word, drug trade name, original title, device manufacturer, drug manufacturer, device trade name, keyword heading word, floating subheading word, candidate term word] \|  \|  \| \|  \|  \| \| 80. \| Truncal veins.mp. [mp=title, abstract, heading word, drug trade name, original title, device manufacturer, drug manufacturer, device trade name, keyword heading word, floating subheading word, candidate term word] \|  \|  \| \|  \|  \| \| 81. \| Ankle flare.mp. [mp=title, abstract, heading word, drug trade name, original title, device manufacturer, drug manufacturer, device trade name, keyword heading word, floating subheading word, candidate term word] \|  \|  \| \|  \|  \| \| 82. \| CEAP.mp. [mp=title, abstract, heading word, drug trade name, original title, device manufacturer, drug manufacturer, device trade name, keyword heading word, floating subheading word, candidate term word] \|  \|  \| \|  \|  \| \| 83. \| CEAP classification.mp. [mp=title, abstract, heading word, drug trade name, original title, device manufacturer, drug manufacturer, device trade name, keyword heading word, floating subheading word, candidate term word] \|  \|  \| \|  \|  \| \| 84. \| 43 or 44 or 45 or 46 or 47 or 48 or 49 or 50 or 51 or 52 or 53 or 54 or 55 or 56 or 57 or 58 or 59 or 60 or 61 or 62 or 63 or 64 or 65 or 66 or 67 or 68 or 69 or 70 or 71 or 72 or 73 or 74 or 75 or 76 or 77 or 78 or 79 or 80 or 81 or 82 or 83 \|  \|  \| \|  \|  \| \| 85. \| Skin assessment.mp. \|  \|  \| \|  \|  \| \| 86. \| exp Nursing Assessment/ \|  \|  \| \|  \|  \| \| 87. \| tissue assessment.mp. \|  \|  \| \|  \|  \| \| 88. \| nursing assessment.mp. [mp=title, abstract, heading word, drug trade name, original title, device manufacturer, drug manufacturer, device trade name, keyword heading word, floating subheading word, candidate term word] \|  \|  \| \|  \|  \| \| 89. \| exp Physical Examination/ \|  \|  \| \|  \|  \| \| 90. \| exp Clinical Competence/ \|  \|  \| \|  \|  \| \| 91. \| Vascular assessment.mp. \|  \|  \| \|  \|  \| \| 92. \| Lower limb assessment.mp. \|  \|  \| \|  \|  \| \| 93. \| Doppler assessment.mp. \|  \|  \| \|  \|  \| \| 94. \| exp Ultrasonography, Doppler/ \|  \|  \| \|  \|  \| \| 95. \| Ankle Brachial Index.mp. \|  \|  \| \|  \|  \| \| 96. \| exp Ankle Brachial Index/ \|  \|  \| \|  \|  \| \| 97. \| ABPI.mp. [mp=title, abstract, heading word, drug trade name, original title, device manufacturer, drug manufacturer, device trade name, keyword heading word, floating subheading word, candidate term word] \|  \|  \| \|  \|  \| \| 98. \| exp Ultrasonography, Doppler, Duplex/ \|  \|  \| \|  \|  \| \| 99. \| exp Risk Assessment/ \|  \|  \| \|  \|  \| \| 100. \| risk assessment.mp. [mp=title, abstract, heading word, drug trade name, original title, device manufacturer, drug manufacturer, device trade name, keyword heading word, floating subheading word, candidate term word] \|  \|  \| \|  \|  \| \| 101. \| CEAP Classification.mp. [mp=title, abstract, heading word, drug trade name, original title, device manufacturer, drug manufacturer, device trade name, keyword heading word, floating subheading word, candidate term word] \|  \|  \| \|  \|  \| \| 102. \| Physical assessment.mp. [mp=title, abstract, heading word, drug trade name, original title, device manufacturer, drug manufacturer, device trade name, keyword heading word, floating subheading word, candidate term word] \|  \|  \| \|  \|  \| \| 103. \| 85 or 86 or 87 or 88 or 89 or 90 or 91 or 92 or 93 or 94 or 95 or 96 or 97 or 98 or 99 or 100 or 101 or 102 \|  \|  \| \|  \|  \| \| 104. \| Accuracy.mp. [mp=title, abstract, heading word, drug trade name, original title, device manufacturer, drug manufacturer, device trade name, keyword heading word, floating subheading word, candidate term word] \|  \|  \| \|  \|  \| \| 105. \| Efficacy.mp. [mp=title, abstract, heading word, drug trade name, original title, device manufacturer, drug manufacturer, device trade name, keyword heading word, floating subheading word, candidate term word] \|  \|  \| \|  \|  \| \| 106. \| Effectiveness.mp. [mp=title, abstract, heading word, drug trade name, original title, device manufacturer, drug manufacturer, device trade name, keyword heading word, floating subheading word, candidate term word] \|  \|  \| \|  \|  \| \| 107. \| Differential diagnosis.mp. [mp=title, abstract, heading word, drug trade name, original title, device manufacturer, drug manufacturer, device trade name, keyword heading word, floating subheading word, candidate term word] \|  \|  \| \|  \|  \| \| 108. \| exp Diagnosis, Differential/ \|  \|  \| \|  \|  \| \| 109. \| Timely Diagnosis.mp. [mp=title, abstract, heading word, drug trade name, original title, device manufacturer, drug manufacturer, device trade name, keyword heading word, floating subheading word, candidate term word] \|  \|  \| \|  \|  \| \| 110. \| Prompt diagnosis.mp. [mp=title, abstract, heading word, drug trade name, original title, device manufacturer, drug manufacturer, device trade name, keyword heading word, floating subheading word, candidate term word] \|  \|  \| \|  \|  \| \| 111. \| Early diagnosis.mp. [mp=title, abstract, heading word, drug trade name, original title, device manufacturer, drug manufacturer, device trade name, keyword heading word, floating subheading word, candidate term word] \|  \|  \| \|  \|  \| \| 112. \| exp Diagnosis/ \|  \|  \| \|  \|  \| \| 113. \| exp Delayed Diagnosis/ \|  \|  \| \|  \|  \| \| 114. \| exp Early Diagnosis/ \|  \|  \| \|  \|  \| \| 115. \| exp Missed Diagnosis/ \|  \|  \| \|  \|  \| \| 116. \| exp Nursing Diagnosis/ \|  \|  \| \|  \|  \| \| 117. \| Delayed diagnosis.mp. [mp=title, abstract, heading word, drug trade name, original title, device manufacturer, drug manufacturer, device trade name, keyword heading word, floating subheading word, candidate term word] \|  \|  \| \|  \|  \| \| 118. \| Referral.mp. [mp=title, abstract, heading word, drug trade name, original title, device manufacturer, drug manufacturer, device trade name, keyword heading word, floating subheading word, candidate term word] \|  \|  \| \|  \|  \| \| 119. \| exp "Referral and Consultation"/ \|  \|  \| \|  \|  \| \| 120. \| exp Patient Care/ \|  \|  \| \|  \|  \| \| 121. \| Patient care.mp. [mp=title, abstract, heading word, drug trade name, original title, device manufacturer, drug manufacturer, device trade name, keyword heading word, floating subheading word, candidate term word] \|  \|  \| \|  \|  \| \| 122. \| exp "Delivery of Health Care"/ \|  \|  \| \|  \|  \| \| 123. \| Patient journey.mp. \|  \|  \| \|  \|  \| \| 124. \| Patient trajectory.mp. \|  \|  \| \|  \|  \| \| 125. \| Patient outcomes.mp. \|  \|  \| \|  \|  \| \| 126. \| Clinical outcomes.mp. \|  \|  \| \|  \|  \| \| 127. \| 104 or 105 or 106 or 107 or 108 or 109 or 110 or 111 or 112 or 113 or 114 or 115 or 116 or 117 or 118 or 119 or 120 or 121 or 122 or 123 or 124 or 125 or 126 \|  \|  \| \|  \|  \| \| 128. \| 42 and 84 and 103 and 127 \|  \|  \|  \|  \| |
