# Supplementary material for: A Systematic Review of the Effectiveness of Assessing Skin Changes for Chronic Venous Insufficiency in People With Dark Skin Tones and the Impact on the Patient Journey and Clinical Care
Source: Int J Vasc Med. 2026 Jun 24;2026:8034303. doi: 10.1155/ijvm/8034303 (PMC13291890; doi:10.1155/ijvm/8034303)
Supplement: Supplementary file 5 — Supporting Information 5 File S5: Risk of bias assessment for included studies by study type: (5a) cross‐sectional studies, (5b) cohort studies, (5c) case series and (5d) case report. [file IJVM-2026-8034303-s005.docx]

# Supplementary file 5 – Risk of bias scores

## *Table S5a: Risk of bias – Cross Sectional Study*

| Author (date) | 1) Were the criteria for inclusion in the sample clearly defined? | 2) Were the study subjects and the setting described in detail? | 3) Was the exposure measured in a valid and reliable way? | 4) Were objective, standard criteria used for measurement of the condition? | 5) Were confounding factors identified? | 6) Were strategies to deal with confounding factors stated? | 7) Were the outcomes measured in a valid and reliable way? | 8) Was appropriate statistical analysis used? | Reporting quality rating |
| --- | --- | --- | --- | --- | --- | --- | --- | --- | --- |
| Bai et al (2024) | Unclear | Yes | N / A | Yes | Yes | No | Unclear | Yes | Good |
| Criqui et al (2003) | Unclear | Yes | N / A | Unclear | Yes | Yes | Yes | Yes | Good |
| Danielsson et al (2002) | No | Yes | N / A | Unclear | Yes | No | Yes | Yes | Good |
| de Souza et al (2013) | Yes | Yes | N / A | Yes | Unclear | No | Yes | Yes | Good |
| Dua et al (2016) | Yes | Yes | N / A | Unclear | Yes | Unclear | Unclear | Yes | Good |
| Fronek et al. (2003) | Yes | Yes | N / A | Yes | Yes | Yes | Yes | Yes | Outstanding |
| Kanchanabat (2010) | Yes | Yes | N / A | Yes | Yes | Yes | Yes | Yes | Outstanding |
| Kanchanabat et al. (2017) | Yes | Yes | N / A | Unclear | Yes | No | Unclear | No | Poor |
| Kanchanabat & Srapanavatr (2018) | Yes | Yes | N / A | Yes | Yes | Unclear | Yes | No | Good |
| Kiguchi et al (2023) | Yes | No | N / A | Yes | Yes | Yes | Unclear | Yes | Good |
| Langer et al (2000) | Yes | Yes | N / A | Yes | Yes | Yes | Yes | Yes | Outstanding |
| Paul et al (2011) | Yes | Yes | N / A | Unclear | Yes | Yes | Yes | Yes | Excellent |
| Taengsakul (2023) | Yes | Yes | N / A | No | Yes | Yes | Yes | Yes | Excellent |
| Verma et al (2023) | Yes | Yes | N / A | Yes | Unclear | Unclear | Yes | Yes | Excellent |

(Yes responses on risk of bias scoring for all relevant questions 100% = Outstanding, 75%-99% = Excellent, 50%-74% = Good, Poor <49%)

## *Table S5b: Risk of bias - Cohort Study*

| Author (date) | 1) Were the two (or more) groups similar and recruited from the same population? | 2) Were the exposures measured similarly to assign people to both exposed and unexposed groups? | 3) Was the exposure measured in a valid and reliable way? | 4) Were confounding factors identified? | 5) Were strategies to deal with confounding factors stated? | 6) Were the groups/participants free of the outcome at the start of the study (or at the moment of exposure)? | 7) Were the outcomes measured in a valid and reliable way? | 8) Was the follow up time reported and sufficient to be long enough for outcomes to occur? | 9) Was follow up complete, and if not, were the reasons to loss to follow up described and explored? | 10) Were strategies to address incomplete follow up utilized? | 11) Was appropriate statistical analysis used? | Reporting quality rating |
| --- | --- | --- | --- | --- | --- | --- | --- | --- | --- | --- | --- | --- |
| Alsheekh et al (2017) | No | N / A | Unclear | Unclear | No | Yes | Yes | Yes | Unclear | Unclear | Yes | Poor |
| Cho et al (2022) | No | N / A | Unclear | Yes | Yes | Yes | Yes | Yes | No | No | Yes | Good |
| Lakhwani at al (2013) | Unclear | N / A | Yes | No | No | No | Unclear | Yes | Unclear | Unclear | Unclear | Poor |
| Pappas et al (2020) | Unclear | N / A | Unclear | No | No | No | Unclear | No | Yes | Yes* | Yes | Poor |
| Pinto Rodríguez et al., (2024) | Yes | N / A | Yes | Yes | Yes | Unclear | Unclear | Yes | No | No | Yes | Good |
| Zil-E-Ali et al. (2023) | Yes | N / A | Yes | Yes | No | Unclear | Unclear | Unclear | Yes | Yes* | Yes | Good |

(* = None lost to follow-up)

(Yes responses on risk of bias scoring for all relevant questions 100% = Outstanding, 75%-99% = Excellent, 50%-74% = Good, Poor <49%)

## *Table S5c: Risk of bias – Case Series*

| Author (date) | 1) Were there clear criteria for inclusion in the case series? | 2) Were there clear criteria for inclusion in the case series? | 3) Were valid methods used for identification of the condition for all  participants included in the case series? | 4) Did the case series have consecutive inclusion of participants? | 5) Did the case series have complete inclusion of participants? | 6) Was there clear reporting of the demographics of the participants in the study? | 7) Was there clear reporting of clinical information of the participants? | 8) Were the outcomes or follow-up results of cases clearly reported? | 9) Was there clear reporting of the presenting sites’/clinics’ demographic  information? | 10) Was statistical analysis appropriate? | Reporting quality rating |
| --- | --- | --- | --- | --- | --- | --- | --- | --- | --- | --- | --- |
| Taofan et al. (2023) | No | Yes | Yes | No | No | No | Yes | Yes | No | N/A | Poor |

(Yes responses on risk of bias scoring for all relevant questions 100% = Outstanding, 75%-99% = Excellent, 50%-74% = Good, Poor <49%)

## Table S5d: Risk of bias – Case report

| Author (date) | 1) Were patient’s demographic characteristics clearly described? | 2) Was the patient’s history clearly described and presented as a timeline? | 3) Was the current clinical condition of the patient on presentation clearly described? | 4) Were diagnostic tests or assessment methods and the results clearly described? | 5) Was the intervention(s) or treatment procedure(s) clearly described? | 6) Was the post-intervention clinical condition clearly described? | 7) Were adverse events (harms) or unanticipated events identified and described? | 8) Does the case report provide takeaway lessons? | Reporting quality rating |
| --- | --- | --- | --- | --- | --- | --- | --- | --- | --- |
| Balasubramanyam et al. (2018) | Yes | Yes | Yes | Yes | Yes | Unclear | No | Yes | Good |

(Yes responses on risk of bias scoring for all relevant questions 100% = Outstanding, 75%-99% = Excellent, 50%-74% = Good, Poor <49%)
