# Supplementary material for: A Systematic Review of the Effectiveness of Assessing Skin Changes for Chronic Venous Insufficiency in People With Dark Skin Tones and the Impact on the Patient Journey and Clinical Care
Source: Int J Vasc Med. 2026 Jun 24;2026:8034303. doi: 10.1155/ijvm/8034303 (PMC13291890; doi:10.1155/ijvm/8034303)
Supplement: Supplementary file 6 — Supporting Information 6 File S6: Sensitivity analysis showing both inclusion and exclusion of studies rated poor reporting quality. [file IJVM-2026-8034303-s006.docx]

**Supplementary file 6. – S*ensitivity analysis showing both inclusion and exclusion of studies rated “poor” reporting quality,***

***6a) Meta-analysis of proportions of people with C1 skin changes between people from Black and White ethnicity with and without inclusion of studies rated “poor”.***

| ***Data excluding studies rated “poor”*** | ***Data from all studies.*** |
| --- | --- |
| ***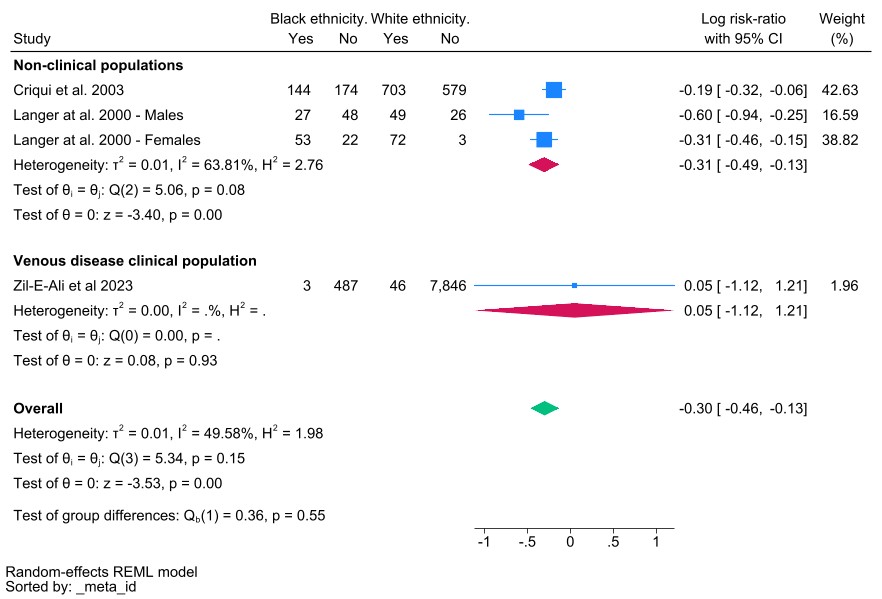***   \| Study \| Black ethnicity \| \| White ethnicity \| \| Log risk-ratio \| [95% conf. interval] \| \| % weight \| \| --- \| --- \| --- \| --- \| --- \| --- \| --- \| --- \| --- \| \|  \| Yes \| No \| Yes \| No \|  \|  \| \|  \| \| ***Non-Clinical populations*** \| \| \| \| \| \| \| \| \| \| Criqui et al. 2003 \| 144 \| 174 \| 703 \| 579 \| -0.191 \| -0.322 \| -0.061 \| 42.62 \| \| Langer at al. 2000 - Males \| 27 \| 48 \| 49 \| 26 \| -0.596 \| -0.940 \| -0.252 \| 18.10 \| \| Langer at al. 2000 - Females \| 53 \| 22 \| 72 \| 3 \| -0.306 \| -0.459 \| -0.153 \| 39.29 \| \| theta \|  \|  \|  \|  \| -0.310 \| -0.488 \| -0.131 \|  \| \| ***Venous disease clinical populations*** \| \| \| \| \| \| \| \| \| \| Zil-E-Ali et al. 2023 \| 3 \| 487 \| 46 \| 7,846 \| 0.049 \| -1.115 \| 1.214 \| 1.96 \| \| theta \|  \|  \|  \|  \| 0.05 \| -1.12 \| 1.21 \|  \| \| ***Overall*** \| \| \| \| \| \| \| \| \| \| theta \|  \|  \|  \|  \| -0.30 \| -0.46 \| -0.13 \|  \|   REML model  **Overall:**  Heterogeneity: I^2^ = 49.58%  Z = -3.53, p<0.01  95% prediction interval for theta: [-0.899, 0.302] *(Antilog = 0.41, 1.35)* | *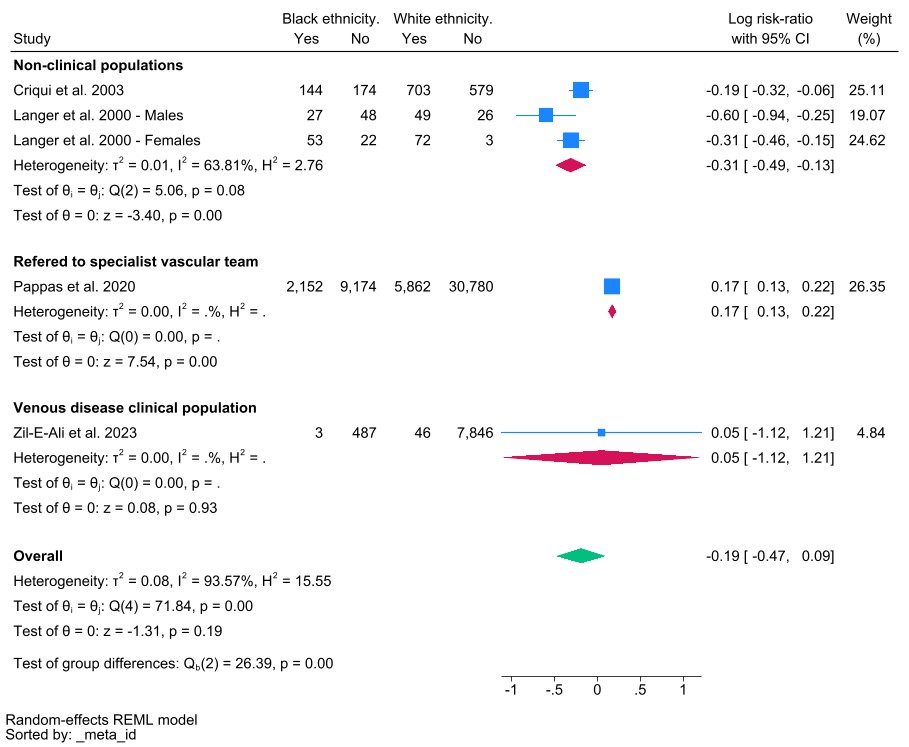*   \| Study \| Black ethnicity \| \| White ethnicity \| \| Log risk-ratio \| [95% conf. interval] \| \| % weight \| \| --- \| --- \| --- \| --- \| --- \| --- \| --- \| --- \| --- \| \|  \| Yes \| No \| Yes \| No \|  \|  \| \|  \| \| ***Non-Clinical populations*** \| \| \| \| \| \| \| \| \| \| Criqui et al. 2003 \| 144 \| 174 \| 703 \| 579 \| -0.191 \| -0.322 \| -0.061 \| 25.11 \| \| Langer at al. 2000 - Males \| 27 \| 48 \| 49 \| 26 \| -0.596 \| -0.940 \| -0.252 \| 19.07 \| \| Langer at al. 2000 - Females \| 53 \| 22 \| 72 \| 3 \| -0.306 \| -0.459 \| -0.153 \| 24.62 \| \| theta \|  \|  \|  \|  \| -0.310 \| -0.488 \| -0.131 \|  \| \| ***Referred to specialist vascular team*** \| \| \| \| \| \| \| \| \| \| Pappas et al. 2020 \| 2152 (est) \| 9174 (est) \| 5862 (est) \| 30,780 (est) \| 0.172 \| 0.127 \| 0.217 \| 26.35 \| \| theta \|  \|  \|  \|  \| 0.17 \| 0.13 \| 0.22 \|  \| \| ***Venous disease clinical populations*** \| \| \| \| \| \| \| \| \| \| Zil-E-Ali et al. 2023 \| 3 \| 487 \| 46 \| 7,846 \| 0.049 \| -1.115 \| 1.214 \| 4.84 \| \| theta \|  \|  \|  \|  \| 0.05 \| -1.12 \| 1.21 \|  \| \| ***Overall*** \| \| \| \| \| \| \| \| \| \| theta \|  \|  \|  \|  \| -0.19 \| -0.47 \| 0.09 \|  \|   *REML Model*  **Overall:**  Heterogeneity: I^2^ = 93.57%  Z = -1.31, p=0.19  95% prediction interval for theta: [-1.194, 0.815] *(Antilog = 0.30, 2.26)* |

Note: This was reported as Telangiectasis (Spider veins) by Criqui 2003 & Langer 2000. This was reported as CEAP C1 by Pappas 2020 & Zil-E-Ali 2023 (where highest classification on CEAP is reported).

***6b) Meta-analysis of proportions of people with C2 skin changes between people from Black and White ethnicity with and without inclusion of studies rated “poor”.***

| ***Data excluding studies rated “poor”*** | ***Data from all studies.*** |
| --- | --- |
| ***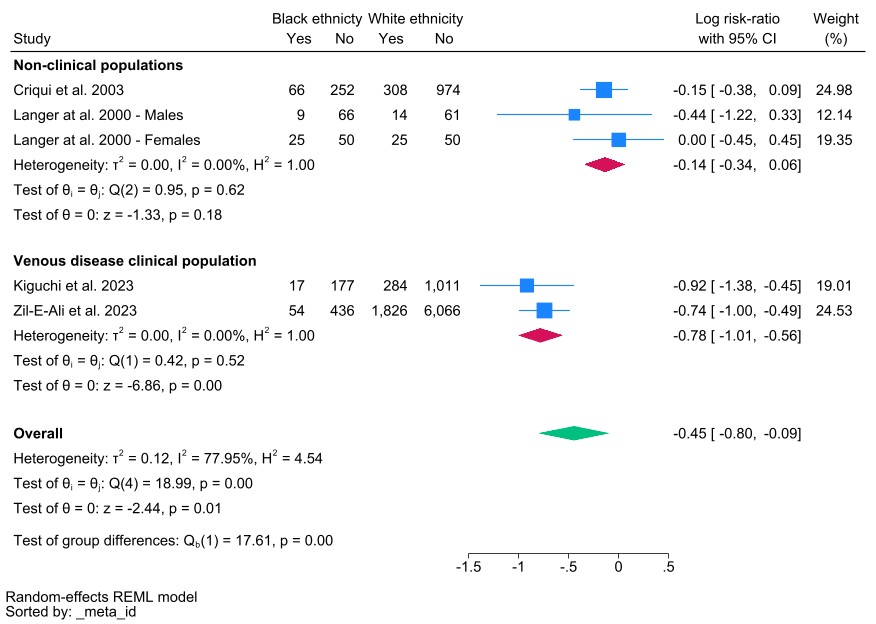***   \| Study \| Black ethnicity \| \| White ethnicity \| \| Log risk-ratio \| [95% conf. interval] \| \| % weight \| \| --- \| --- \| --- \| --- \| --- \| --- \| --- \| --- \| --- \| \|  \| Yes \| No \| Yes \| No \|  \|  \| \|  \| \| ***Non-Clinical populations*** \| \| \| \| \| \| \| \| \| \| Criqui et al. 2003 \| 66 \| 252 \| 308 \| 974 \| -0.15 \| -0.38 \| 0.09 \| 24.98 \| \| Langer at al. 2000 - Males \| 9 \| 66 \| 14 \| 61 \| -0.44 \| -1.22 \| 0.33 \| 12.14 \| \| Langer at al. 2000 - Females \| 25 \| 50 \| 25 \| 50 \| 0.00 \| -0.45 \| 0.45 \| 19.35 \| \| theta \|  \|  \|  \|  \| -0.14 \| -0.34 \| 0.06 \|  \| \| ***Venous disease clinical populations*** \| \| \| \| \| \| \| \| \| \| Kiguchi et al. 2023 \| 17 \| 177 \| 284 \| 1,011 \| -0.917 \| -1.383 \| -0.452 \| 19.01 \| \| Zil-E-Ali et al. 2023 \| 54 \| 436 \| 1,826 \| 6,066 \| -0.742 \| -0.996 \| -0.487 \| 24.53 \| \| theta \|  \|  \|  \|  \| -0.78 \| -1.01 \| -0.56 \|  \| \| ***Overall*** \| \| \| \| \| \| \| \| \| \| theta \|  \|  \|  \|  \| -0.45 \| -0.80 \| -0.09 \|  \|   REML model  **Overall:**  Heterogeneity: I^2^ = 77.95%  Z = -2.44, p=0.01  95% prediction interval for theta: [-1.689, 0.796] *(Anti-log = 0.18, 2.22)* | *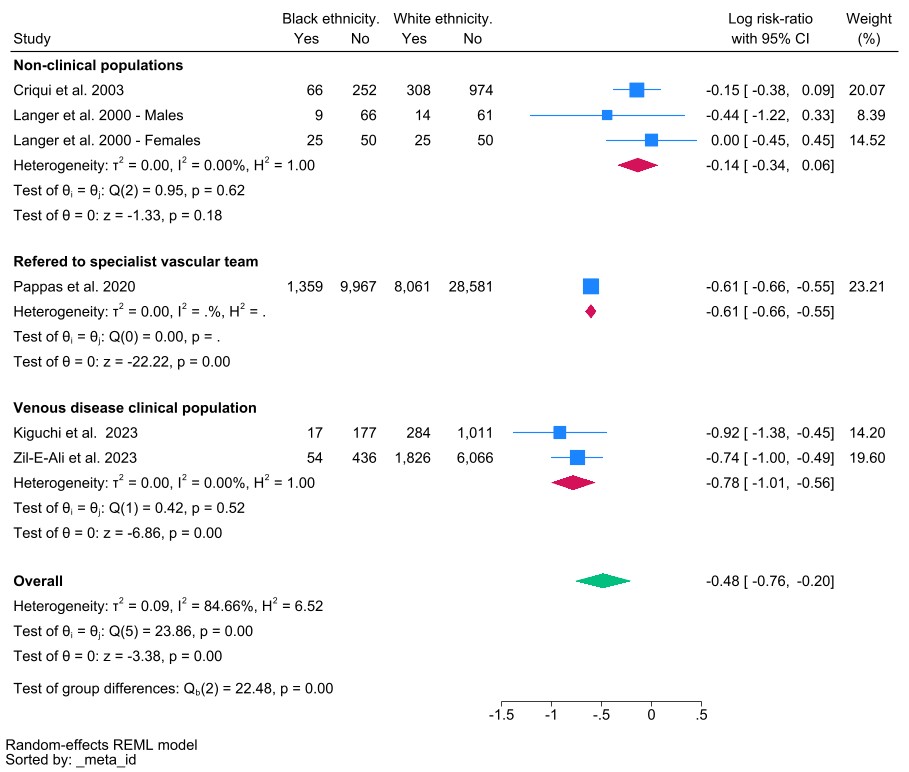*   \| Study \| Black ethnicity \| \| White  ethnicity \| \| Log risk-ratio \| [95% conf. interval] \| \| % weight \| \| --- \| --- \| --- \| --- \| --- \| --- \| --- \| --- \| --- \| \|  \| Yes \| No \| Yes \| No \|  \|  \| \|  \| \| ***Non-Clinical populations*** \| \| \| \| \| \| \| \| \| \| Criqui et al. 2003 \| 66 \| 252 \| 308 \| 974 \| -0.15 \| -0.38 \| 0.09 \| 20.07 \| \| Langer at al. 2000 - Males \| 9 \| 66 \| 14 \| 61 \| -0.44 \| -1.22 \| 0.33 \| 8.39 \| \| Langer at al. 2000 - Females \| 25 \| 50 \| 25 \| 50 \| 0.00 \| -0.45 \| 0.45 \| 14.52 \| \| theta \|  \|  \|  \|  \| -0.14 \| -0.34 \| 0.06 \|  \| \| ***Referred to specialist vascular team*** \| \| \| \| \| \| \| \| \| \| Pappas et al. 2020 \| 1,359 (est) \| 9,967 (est) \| 8,061 (est) \| 28,581 (est) \| -0.61 \| -0.66 \| -0.55 \| 23.21 \| \| theta \|  \|  \|  \|  \|  \|  \|  \|  \| \| ***Venous disease clinical populations*** \| \| \| \| \| \| \| \| \| \| Kiguchi et al. 2023 \| 17 \| 177 \| 284 \| 1,011 \| -0.92 \| -1.38 \| -0.45 \| 14.20 \| \| Zil-E-Ali et al. 2023 \| 54 \| 436 \| 1,826 \| 6,066 \| -0.74 \| -1.00 \| -0.49 \| 19.60 \| \| theta \|  \|  \|  \|  \| -0.78 \| -1.01 \| -0.56 \|  \| \| ***Overall*** \| \| \| \| \| \| \| \| \| \| theta \|  \|  \|  \|  \| -0.48 \| -0.76 \| -0.20 \|  \|   *REML Model*  **Overall:**  Heterogeneity: I^2^ = 84.66%  Z = -3.38, p<0.01  95% prediction interval for theta: [-1.393, 0.427] *(Anti-log = 0.29, 1.53)* |

Note: This was reported as Varicose veins by Criqui 2003 & Langer 2000. This was reported as CEAP C2 by Pappas 2020, Kiguchi 2023 & Zil-E-Ali 2023 (where highest classification on CEAP is reported)

***6c) Meta-analysis of proportions of people with C3 skin changes between people from Black and White ethnicity with and without inclusion of studies rated “poor”.***

| ***Data excluding studies rated “poor”*** | ***Data from all studies.*** |
| --- | --- |
| ***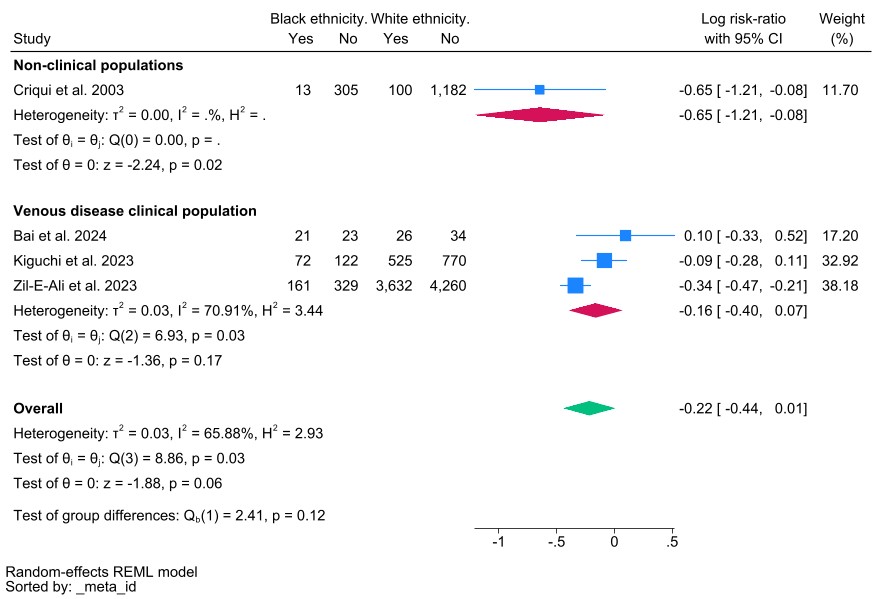***   \| Study \| Black ethnicity \| \| White ethnicity \| \| Log risk-ratio \| [95% conf. interval] \| \| % weight \| \| --- \| --- \| --- \| --- \| --- \| --- \| --- \| --- \| --- \| \|  \| Yes \| No \| Yes \| No \|  \|  \| \|  \| \| ***Non-Clinical populations*** \| \| \| \| \| \| \| \| \| \| Criqui et al. 2003 \| 13 \| 305 \| 100 \| 1,182 \| -0.646 \| -1.211 \| -0.081 \| 11.70 \| \| theta \|  \|  \|  \|  \| -0.65 \| -1.21 \| -0.08 \|  \| \| ***Venous disease clinical populations*** \| \| \| \| \| \| \| \| \| \| Bai et al. 2024 \| 21 \| 23 \| 26 \| 34 \| 0.097 \| -0.327 \| 0.520 \| 17.20 \| \| Kiguchi et al. 2023 \| 72 \| 122 \| 525 \| 770 \| -0.088 \| -0.283 \| 0.106 \| 32.92 \| \| Zil-E-Ali et al. 2023 \| 161 \| 329 \| 3,632 \| 4.260 \| -0.337 \| -0.466 \| -0.208 \| 38.18 \| \| theta \|  \|  \|  \|  \| -0.16 \| -0.40 \| 0.07 \|  \| \| ***Overall*** \| \| \| \| \| \| \| \| \| \| theta \|  \|  \|  \|  \| -0.217 \| -0.442 \| 0.009 \|  \|   REML model  **Overall:**  Heterogeneity: I^2^ = 65.88%  Z = -1.88, p=0.06  95% prediction interval for theta: [-1.116, 0.683] | *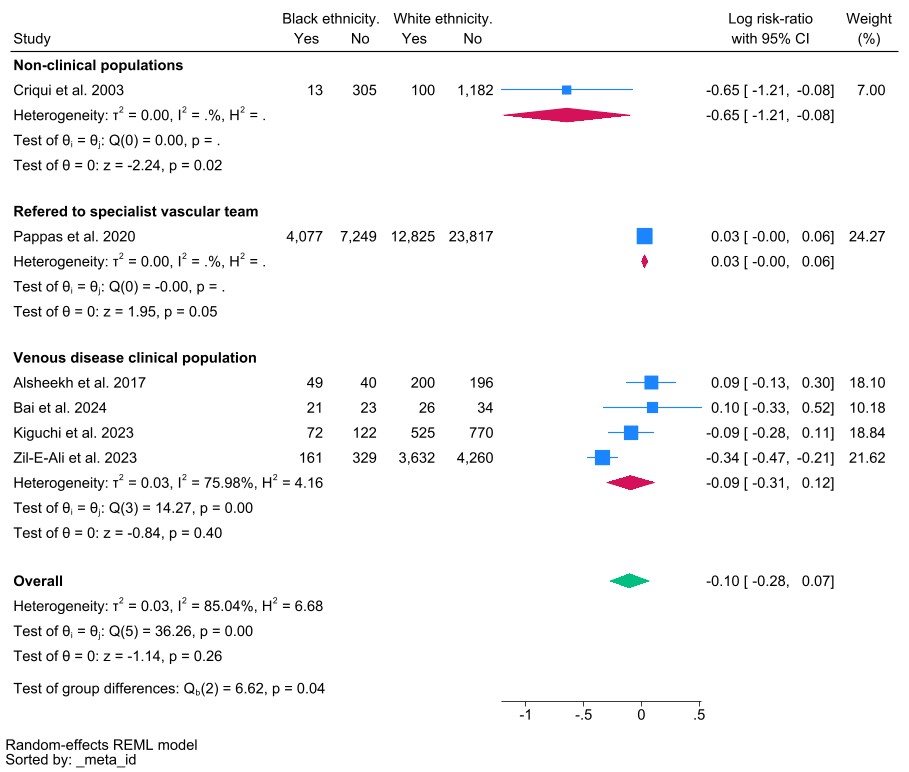*   \| Study \| Black ethnicity \| \| White  ethnicity \| \| Log risk-ratio \| [95% conf. interval] \| \| % weight \| \| --- \| --- \| --- \| --- \| --- \| --- \| --- \| --- \| --- \| \|  \| Yes \| No \| Yes \| No \|  \|  \| \|  \| \| ***Non-Clinical populations*** \| \| \| \| \| \| \| \| \| \| Criqui et al. 2003 \| 13 \| 305 \| 100 \| 1,182 \| -0.646 \| -1.211 \| -0.081 \| 7.00 \| \| theta \|  \|  \|  \|  \| -0.646 \| -1.211 \| -0.081 \|  \| \| ***Referred to specialist vascular team*** \| \| \| \| \| \| \| \| \| \| Pappas et al. 2020 \| 4,077 (est) \| 7,249 (est) \| 12,825 (est) \| 23,817 (est) \| 0.028 \| -0.000 \| 0.056 \| 24.27 \| \| theta \|  \|  \|  \|  \| 0.028 \| -0.000 \| 0.056 \|  \| \| ***Venous disease clinical populations*** \| \| \| \| \| \| \| \| \| \| Alsheekh et al. 2017 \| 49 \| 40 \| 200 \| 196 \| 0.086 \| -0.125 \| 0.298 \| 18.10 \| \| Bai et al. 2024 \| 21 \| 23 \| 26 \| 34 \| 0.097 \| -0.327 \| 0.520 \| 10.18 \| \| Kiguchi et al. 2023 \| 72 \| 122 \| 525 \| 770 \| -0.088 \| -0.283 \| 0.106 \| 18.84 \| \| Zil-E-Ali et al. 2023 \| 161 \| 329 \| 3,632 \| 4.260 \| -0.337 \| -0.466 \| -0.208 \| 21.62 \| \| theta \|  \|  \|  \|  \| -0.093 \| -0.308 \| 0.123 \|  \| \| ***Overall*** \| \| \| \| \| \| \| \| \| \| theta \|  \|  \|  \|  \| -0.102 \| -0.279 \| 0.074 \|  \|   *REML Model*  **Overall:**  Heterogeneity: I^2^ = 85.04%  Z = -1.14, p = 0.26  95% prediction interval for theta: [-0.668, 0.463] |

Note: This was reported as oedema by Criqui 2003. This was reported as CEAP C3 by Pappas 2020, Alsheekh 2017, Bai 2024, Kiguchi 2023 & Zil-E-Ali 2023 (where highest classification on CEAP is reported)

***6d) Meta-analysis of proportions of people with C4 skin changes between people from Black and White ethnicity with and without inclusion of studies rated “poor”.***

| ***Data excluding studies rated “poor”*** | ***Data from all studies.*** |
| --- | --- |
| ***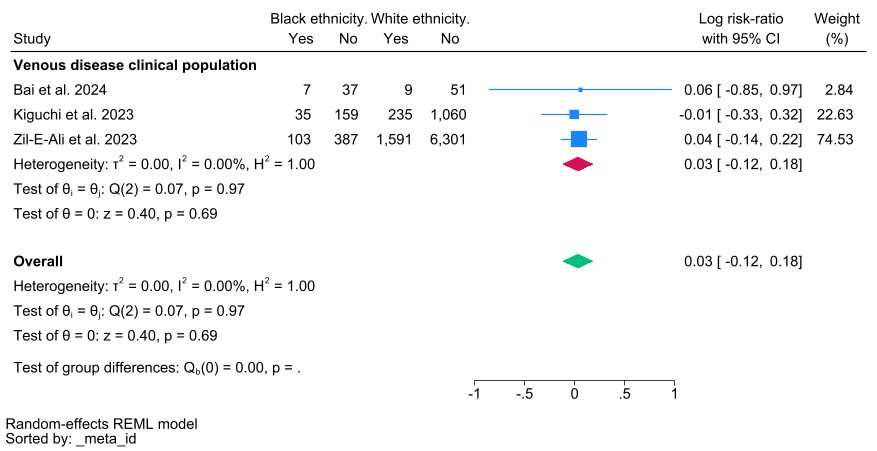***   \| Study \| Black ethnicity \| \| White ethnicity \| \| Log risk-ratio \| [95% conf. interval] \| \| % weight \| \| --- \| --- \| --- \| --- \| --- \| --- \| --- \| --- \| --- \| \|  \| Yes \| No \| Yes \| No \|  \|  \| \|  \| \| ***Venous disease clinical populations*** \| \| \| \| \| \| \| \| \| \| Bai et al. 2024 \| 7 \| 37 \| 9 \| 51 \| 0.059 \| -0.849 \| 0.967 \|  \| \| Kiguchi et al. 2023 \| 35 \| 159 \| 235 \| 1,060 \| -0.006 \| -0.327 \| 0.316 \|  \| \| Zil-E-Ali et al. 2023 \| 103 \| 387 \| 1,591 \| 6,301 \| 0.042 \| -0.135 \| 0.219 \|  \| \| theta \|  \|  \|  \|  \| 0.032 \| -0.121 \| 0.184 \|  \| \| ***Overall*** \| \| \| \| \| \| \| \| \| \| theta \|  \|  \|  \|  \| 0.032 \| -0.121 \| 0.184 \|  \|   REML model  **Overall:**  Heterogeneity: I^2^ = 0.00%  Z = 0.40, p=0.69  95% prediction interval for theta: [-0.960, 1.023] | *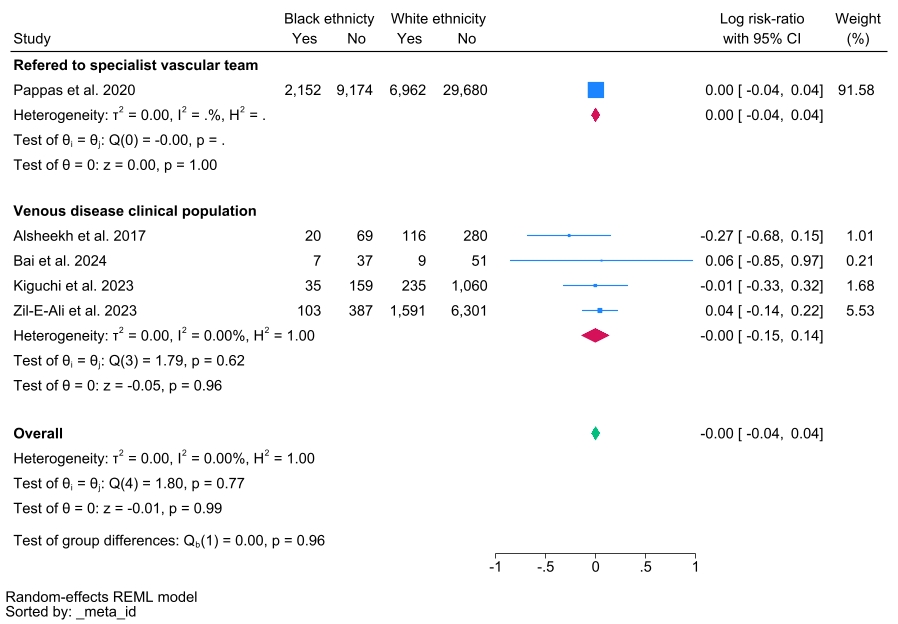*   \| Study \| Black ethnicity \| \| White  ethnicity \| \| Log risk-ratio \| [95% conf. interval] \| \| % weight \| \| --- \| --- \| --- \| --- \| --- \| --- \| --- \| --- \| --- \| \|  \| Yes \| No \| Yes \| No \|  \|  \| \|  \| \| ***Referred to specialist vascular team*** \| \| \| \| \| \| \| \| \| \| Pappas et al. 2020 \| 2,152 (est) \| 9,174 (est) \| 6,962 (est) \| 29,680 (est) \| 0.000 \| -0.043 \| 0.044 \| 91.58 \| \| theta \|  \|  \|  \|  \| 0.00 \| -0.04 \| 0.04 \|  \| \| ***Venous disease clinical populations*** \| \| \| \| \| \| \| \| \| \| Alsheekh et al. 2017 \| 20 \| 69 \| 116 \| 280 \| -0.265 \| -0.680 \| 0.150 \| 1.01 \| \| Bai et al. 2024 \| 7 \| 37 \| 9 \| 51 \| 0.059 \| -0.849 \| 0.967 \| 0.21 \| \| Kiguchi et al. 2023 \| 35 \| 159 \| 235 \| 1,060 \| -0.006 \| -0.327 \| 0.316 \| 1.68 \| \| Zil-E-Ali et al. 2023 \| 103 \| 387 \| 1,591 \| 6,301 \| 0.042 \| -0.135 \| 0.219 \| 5.53 \| \| theta \|  \|  \|  \|  \| -0.00 \| -0.15 \| 0.14 \|  \| \| ***Overall*** \| \| \| \| \| \| \| \| \| \| theta \|  \|  \|  \|  \| -0.00 \| -0.04 \| 0.04 \|  \|   *REML Model*  **Overall:**  Heterogeneity: I^2^ = 0.00%  Z = -0.01, p = 0.99  95% prediction interval for theta: [-0.068, 0.067] |

Note: This was reported as CEAP C4 by Pappas 2020, Alsheekh 2017, Bai 2024, Kiguchi 2023 & Zil-E-Ali 2023 (where highest classification on CEAP is reported).

***6e) Meta-analysis of proportions of people with C5 & C6 between people from Black and White ethnicity with and without inclusion of studies rated “poor”.***

| ***Data excluding studies rated “poor”*** | ***Data from all studies.*** |
| --- | --- |
| ***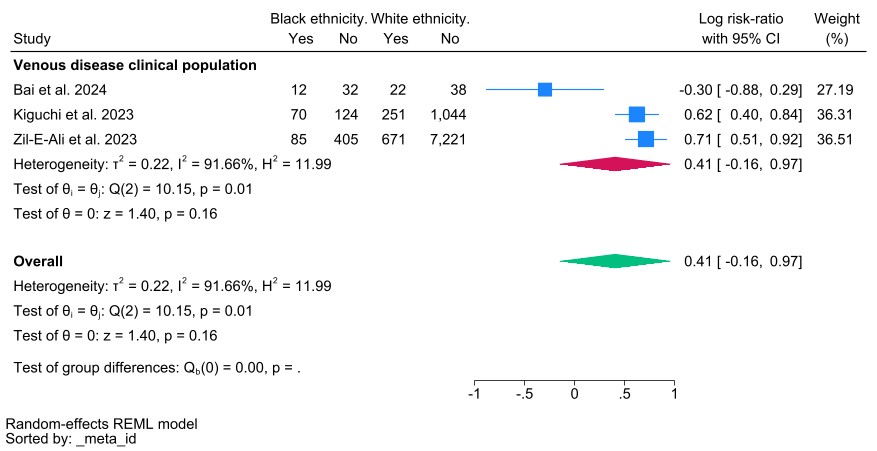***   \| Study \| Black ethnicity \| \| White ethnicity \| \| Log risk-ratio \| [95% conf. interval] \| \| % weight \| \| --- \| --- \| --- \| --- \| --- \| --- \| --- \| --- \| --- \| \|  \| Yes \| No \| Yes \| No \|  \|  \| \|  \| \| ***Venous disease clinical populations*** \| \| \| \| \| \| \| \| \| \| Bai et al. 2024 \| 12 \| 32 \| 22 \| 38 \| -0.296 \| -0.882 \| 0.290 \| 27.19 \| \| Kiguchi et al. 2023 \| 70 \| 124 \| 251 \| 1,044 \| 0.621 \| 0.404 \| 0.839 \| 36.31 \| \| Zil-E-Ali et al. 2023 \| 85 \| 405 \| 671 \| 7,221 \| 0.713 \| 0.507 \| 0.919 \| 36.51 \| \| theta \|  \|  \|  \|  \| 0.405 \| -0.161 \| 0.971 \|  \| \| ***Overall*** \| \| \| \| \| \| \| \| \| \| theta \|  \|  \|  \|  \| 0.405 \| -0.161 \| 0.971 \|  \|   REML model  **Overall:**  Heterogeneity: I^2^ = 91.66%  Z = 1.40, p=0.16  95% prediction interval for theta: [-6.563, 7.374] | *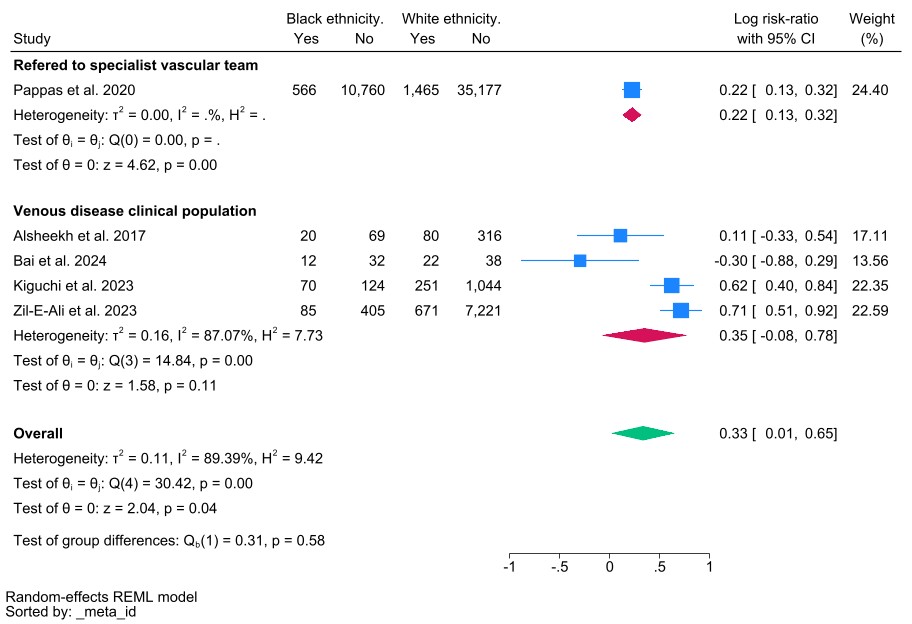*   \| Study \| Black ethnicity \| \| White  ethnicity \| \| Log risk-ratio \| [95% conf. interval] \| \| % weight \| \| --- \| --- \| --- \| --- \| --- \| --- \| --- \| --- \| --- \| \|  \| Yes \| No \| Yes \| No \|  \|  \| \|  \| \| ***Referred to specialist vascular team*** \| \| \| \| \| \| \| \| \| \| Pappas et al. 2020 \| 566 (est) \| 10,760 (est) \| 1,465 (est) \| 35,177 (est) \| 0.223 \| 0.128 \| 0.318 \| 24.40 \| \| theta \|  \|  \|  \|  \| 0.223 \| 0.128 \| 0.318 \|  \| \| ***Venous disease clinical populations*** \| \| \| \| \| \| \| \| \| \| Alsheekh et al. 2017 \| 20 \| 69 \| 80 \| 316 \| 0.106 \| -0.326 \| 0.539 \| 17.11 \| \| Bai et al. 2024 \| 12 \| 32 \| 22 \| 38 \| -0.296 \| -0.882 \| 0.290 \| 13.56 \| \| Kiguchi et al. 2023 \| 70 \| 124 \| 251 \| 1,044 \| 0.621 \| 0.404 \| 0.839 \| 22.35 \| \| Zil-E-Ali et al. 2023 \| 85 \| 405 \| 671 \| 7,221 \| 0.713 \| 0.507 \| 0.919 \| 22.59 \| \| theta \|  \|  \|  \|  \| 0.348 \| -0.084 \| 0.779 \|  \| \| ***Overall*** \| \| \| \| \| \| \| \| \| \| theta \|  \|  \|  \|  \| 0.332 \| 0.013 \| 0.652 \|  \|   *REML Model*  **Overall:**  Heterogeneity: I^2^ = 89.39%  Z = 2.04, p = 0.04  95% prediction interval for theta: [-0.828, 1.493] *(Anti-log = 0.44, 4.45)* |

Note: This was the cumulative values of CEAP C5 & CEAP C6 (where highest classification on CEAP is reported).
